# Strand-displacement amplification reduces structure-induced PCR bias and preserves structurally diverse aptamers during SELEX

**DOI:** 10.1093/nar/gkag1186

**Published:** 2026-11-11

**Authors:** Hwayeon Jeong, Chang Beom Jeong, Jae Yun Moon, Daechan Park, Cheulhee Jung

**Affiliations:** Department of Biotechnology, College of Life Sciences and Biotechnology, Korea University, 145, Anam-ro, Seongbuk-gu, Seoul 02841, Republic of Korea; Department of Molecular Science and Technology, College of Advanced Bio-Convergence Engineering, Ajou University, Suwon 16499, Republic of Korea; Department of Molecular Science and Technology, College of Advanced Bio-Convergence Engineering, Ajou University, Suwon 16499, Republic of Korea; Department of Molecular Science and Technology, College of Advanced Bio-Convergence Engineering, Ajou University, Suwon 16499, Republic of Korea; Department of Biotechnology, College of Life Sciences and Biotechnology, Korea University, 145, Anam-ro, Seongbuk-gu, Seoul 02841, Republic of Korea

## Abstract

Stable secondary structures are important for aptamer folding and target recognition, but they can introduce structure-dependent amplification bias during Systematic Evolution of Ligands by Exponential Enrichment (SELEX), with G-quadruplexes (G4s) representing the most demanding case. Here, we introduce strand-displacement SELEX (SD-SELEX), which employs a polymerase with robust 5′→3′ strand-displacement activity to improve the amplification of structured templates. Comparative analyses showed that SD polymerase efficiently amplified hairpin- and G4-forming templates, whereas Taq polymerase exhibited structure-dependent amplification variability. In parallel thrombin SELEX experiments, SD-SELEX achieved 37-fold greater structural preservation and 65-fold higher batch-to-batch reproducibility than Taq-SELEX over 11 rounds. High-throughput sequencing further showed that SD-SELEX maintained G4 motif representation and progressively enriched the known 15-mer thrombin-binding aptamer through round 8, after which it was gradually replaced by newly identified G4 variants, whereas Taq-SELEX progressively depleted these sequences. SD-SELEX also identified novel G4 aptamers with affinities comparable to that of the reference aptamer, whereas representative Taq-SELEX candidates generally showed weaker binding. A parallel selection against VEGF-165 showed the same divergence, with SD-SELEX retaining structurally complex sequences and a previously reported functional motif that were progressively lost under Taq. Thus, SD-SELEX primarily improves aptamer discovery by reducing structure-induced amplification bias and preserving structurally diverse functional candidates, rather than by substantially increasing affinity alone.

## Graphical Abstract

**Graphical Abstract:**
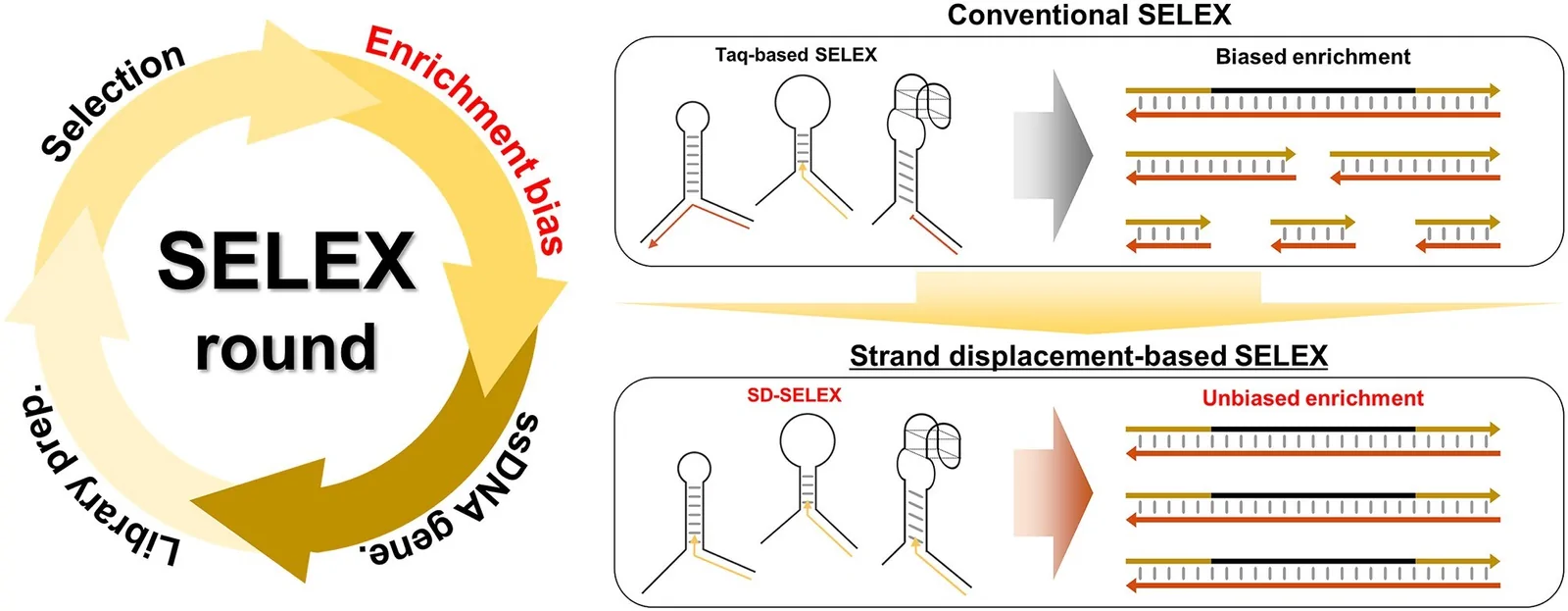

## Introduction

Aptamers are nucleic acid ligands that exhibit antibody-like affinity and specificity, while offering unique advantages such as ease of synthesis, low immunogenicity, and chemical and thermal stability [1]. These properties have enabled broad applications in diagnostics, therapeutics, and drug-delivery platforms, and the FDA approvals of Macugen (2004) and Izervay (2023) underscore their clinical potential [2, 3]. Nevertheless, the success rate of aptamer discovery via SELEX (Systematic Evolution of Ligands by Exponential Enrichment) remains below 30% [4], which has been recognized as a significant barrier to the clinical and industrial translation of aptamers.

Conventional SELEX protocols primarily rely on Taq polymerase [5–8]. However, Taq polymerase has been reported to undergo synthesis stalling and template switching (“jumping polymerase”) when encountering structured templates [9–12]. Stable secondary structures, including hairpins, stem–loops, G-quadruplexes (G4s), and other internally base-paired architectures, can therefore differentially affect amplification efficiency. Because PCR amplification is repeatedly performed throughout successive SELEX rounds, even modest structure-dependent differences in amplification efficiency may accumulate and progressively alter library composition. Consequently, structurally complex sequences may become underrepresented independently of their target-binding properties.

Secondary structure is a fundamental determinant of aptamer folding and molecular recognition. Functional aptamers can adopt diverse architectures, including hairpins, stem–loops, multi-stem structures, and G-quadruplexes (G4s), depending on their sequences and target interactions. Among these, G4 represents a particularly stable structural motif formed by stacked guanine tetrads through Hoogsteen interactions [13], and stable G4 formation in the template strand has been shown to impede DNA polymerase progression [14, 15]. A recent systematic analysis reported potential G4 structures in approximately one-quarter of DNA aptamers and one-sixth of RNA aptamers [16], indicating that G4 represents an important but only one subset of the broader structural landscape of aptamers. Because well-characterized G4 aptamers against thrombin are available, this target provides a tractable readout for testing whether structured candidates are preserved through repeated amplification, although the underlying question applies to structured sequences in general. Therefore, structure-dependent amplification bias during SELEX should be considered across diverse secondary structures rather than exclusively in the context of G4 formation.

To address PCR-associated problems in SELEX, compartmentalized strategies, such as emulsion PCR (emPCR) and droplet digital PCR (ddPCR) have been employed to reduce amplification competition and improve diversity preservation [17–20]. However, their reliance on specialized instrumentation and procedural complexity has limited their general utility, and they do not directly overcome the sensitivity of polymerases to nucleic acid structure. Therefore, a more direct and streamlined solution is needed to mitigate structural bias.

In this study, we introduce strand-displacement-based SELEX (SD-SELEX), which employs a strand-displacement (SD) polymerase as the amplification engine. We first evaluated amplification performance using synthetic templates representing diverse secondary structures, including hairpins, stem–loops, and G4s. We then performed parallel thrombin SELEX experiments using SD and Taq polymerases and analyzed the resulting next generation sequencing (NGS) datasets to determine how amplification chemistry affects the preservation and evolution of sequences with different predicted structural characteristics. To further evaluate the general applicability of SD-SELEX beyond the thrombin model, we also conducted an independent SELEX experiment against vascular endothelial growth factor (VEGF) as an additional protein target. In addition to this broader secondary-structure analysis, G4-containing sequences were examined as a representative class of stable structured aptamers because thrombin provides a well-established model system for G4-dependent aptamer recognition. Together, these experiments allowed us to evaluate structure-induced amplification bias at both the individual-template and population levels and to assess the applicability of SD-based amplification across distinct protein-target SELEX systems.

Collectively, this work demonstrates that amplification chemistry can influence the preservation of structurally diverse sequences during iterative SELEX. By reducing structure-induced amplification bias, SD-SELEX maintains a broader structural sequence space, including hairpin-, stem-loop-, and G4-forming candidates. G4 aptamers provide a representative functional case illustrating how such amplification bias can influence the evolutionary outcome of SELEX.

## Materials and methods

### General

All the DNA oligonucleotides with the sequences listed in Supplementary Table S1 were synthesized and characterized by Integrated DNA Technologies (Coralville, IA, USA). Modified oligonucleotides were then purified using the PAGE or HPLC method. Biotinylated *α*-thrombin and Dynabeads^™^ M-270 Streptavidin were purchased from Invitrogen (Thermo Fisher Scientific, Waltham, MA, USA). Biotinylated human VEGF165 protein and biotinylated human Mucin-1 protein (MUC1) were purchased from ACROBiosystems (Newark, DE, USA). SD HotStart DNA polymerase, 10× SD polymerase reaction buffer, and MgCl_2_ were purchased from Bioron GmbH (Römerberg, Germany). I-StarTaq DNA polymerase and 10× PCR buffer were purchased from Intron Biotechnology (Seongnam, Republic of Korea). Platinum^™^ SuperFi II DNA polymerase and 5× SuperFi II buffer were purchased from Invitrogen. MGIEasy Universal DNA Library Prep Set was purchased from MGI technology (Shenzhen, China). Qubit^™^ ssDNA assay kit was purchased from Invitrogen and DeNovix dsDNA high sensitivity assay kit was purchased from DeNovix Inc. (Wilmington, DE, USA). SYBR gold was purchased from Invitrogen. To prepare the selection buffer for SELEX, NaCl, Tris–HCl, and MgCl_2_ were purchased from Intron Biotechnology, and KCl, CaCl_2_, and Tween 20 were purchased from Sigma–Aldrich (Saint Louis, MO, USA). EDTA and Triton X-100 were purchased from Intron Biotechnology to prepare the reaction buffer and washing buffer for SELEX. Monarch PCR & DNA cleanup kit was purchased from New England Biolabs (Ipswich, MA, USA) to purify the oligonucleotides.

### Comparison of polymerase efficiency

To compare the amplification efficiency using optimized polymerases with SD polymerase (Bioron GmbH), i-StarTaq DNA polymerase (Intron Biotechnology), Platinum^™^ SuperFi II DNA Polymerase (Thermo Fisher Scientific), Q5^®^ Hot Start High-Fidelity DNA Polymerase (New England Biolabs), Phusion Hot Start II High-Fidelity PCR Master Mix (Thermo Fisher Scientific), and OneTaq^®^ DNA Polymerase (New England Biolabs), we performed PCR and qPCR analysis on templates of various structures (Supplementary Table S1).

PCR and real-time qPCR methods were employed to compare the amplification efficiency of these sequences. When using SD polymerase, the reaction involved a pre-denaturation step at 92°C for 2 min, cycles of denaturation at 92°C for 10 s, annealing at 64°C for 10 s, and extension at 68°C for 30 s, followed by a final extension at 68°C for 5 min. When Taq polymerase was used, the reaction involved a pre-denaturation step at 94°C for 2 min, cycles of denaturation at 94°C for 20 s, annealing at 65°C for 10 s, and extension at 72°C for 30 s, followed by a final extension at 72°C for 5 min. When using Platinum^™^ SuperFi II DNA Polymerase, the reaction involved a pre-denaturation step at 98°C for 30 s, cycles of denaturation at 98°C for 10 s, annealing at 60°C for 10 s, and extension at 72°C for 30 s, followed by a final extension at 72°C for 5 min. When Q5^®^ Hot Start High-Fidelity DNA Polymerase was used, the reaction involved a pre-denaturation step at 98°C for 30 s, cycles of denaturation at 94°C for 10 s, annealing at 72°C for 10 s, and extension at 72°C for 30 s, followed by a final extension at 72°C for 2 min. When Phusion Hot Start II High-Fidelity PCR Master Mix was used, the reaction involved a pre-denaturation step at 98°C for 30 s, cycles of denaturation at 98°C for 10 s, annealing at 65°C for 10 seconds, and extension at 72°C for 30 s, followed by a final extension at 72°C for 5 min. When OneTaq^®^ DNA Polymerase was used, the reaction involved a pre-denaturation step at 94°C for 30 s, cycles of denaturation at 94°C for 15 s, annealing at 60°C for 15 s, and extension at 68°C for 15 s, followed by a final extension at 68°C for 5 min. By-product synthesis was analyzed using 2% agarose gel after PCR, and real-time qPCR was conducted to analyze the amplification efficiency.

### Effect of KCl concentration on amplification efficiency

To evaluate the effect of KCl concentration on amplification efficiency, PCR and real-time qPCR analyses were performed using Taq DNA polymerase (New England Biolabs) and templates with different structural characteristics (Supplementary Table S1). Custom reaction buffers contained 10 mM Tris–HCl (pH 8.3), 1.5 mM MgCl₂, and varying concentrations of KCl. For reactions containing 10, 20, 30, 40, or 50 mM KCl, the NaCl concentration was adjusted to 40, 30, 20, 10, or 0 mM, respectively, to maintain a total KCl/NaCl concentration of 50 mM. An additional buffer containing 0 mM KCl and 50 mM NaCl was used for qPCR analysis.

PCR was performed with templates #3, #5, #7, and #9 using KCl concentrations ranging from 10 to 50 mM. The reaction involved a pre-denaturation step at 95°C for 30 s, followed by 25 cycles of denaturation at 95°C for 15 s, annealing at 60°C for 15 s, and extension at 68°C for 30 s, with a final extension at 68°C for 5 min. Amplification products were analyzed on 2% agarose gels. Real-time qPCR was additionally performed using the G4-containing template #3 and non-G4 template #1 at 0, 10, 30, and 50 mM KCl to assess whether the effect of KCl on amplification was structure dependent.

### PCR cycle regulation

PCR condition adjustments are required to minimize bias with comparing SD and Taq polymerase-based SELEX groups. Accordingly, we fine-tuned the PCR conditions to minimize the PCR bias arising from the diversity of aptamer libraries and the substantial template input. Similar experimental cases have been reported in previous studies [9, 21]. However, in these studies, various PCR conditions had to be performed for each SELEX round, leading to significant consumption of the eluent obtained post-selection. In contrast, our study minimized the volume of SELEX eluent used per round and employed qPCR to predict the optimal number of PCR cycles in advance, thereby implementing an efficient PCR cycle regulation method (Supplementary Fig. S1).

The selected library quantity of eluent from each round of SELEX can vary. Therefore, an analysis was conducted to investigate the relationship between qPCR and PCR cycles using varying template amounts to demonstrate that the results of PCR cycle regulation are consistent regardless of the template used. We conducted real-time qPCR on 1 μl samples of aptamer libraries at concentrations of 10^5^, 10^7^, and 10^9^ molecules/μl and assessed the occurrence of bias based on the amplification patterns for each PCR cycle, as observed through native gel electrophoresis (Supplementary Fig. S1B and C). We could choose PCR cycle conditions that eliminated any potential biases related to the quantity of input aptamer library. We then converted these selected conditions into PCR cycles for 25 μl of aptamer libraries at a concentration of 10^9^ molecules/μl, which were then utilized for enrichment during actual SELEX. This conversion was facilitated through a real-time qPCR standard curve that accounted for the quantity of aptamer libraries. Consequently, we executed PCR at PCR cycles for a 25 μl volume of aptamer libraries at a concentration of 10^9^ molecules/μl and within the neighboring cycle range (−1 cycle/+1 cycle), followed by confirmation through native gel electrophoresis (Supplementary Fig. S1D). We adjusted them based on real-time qPCR Ct values and selected unbiased PCR cycle conditions for each SELEX round. This method was applied to the experimental groups of SD polymerase and Taq polymerase (Supplementary Fig. S1E). In the control experiments, we determined PCR cycles based on the point of saturation amplification as determined by real-time qPCR results, thus facilitating enrichment within the SELEX rounds.

### SELEX procedure

We prepared the SELEX protocol for this study by referring to previously reported procedures (Supplementary Table S2). The SELEX process comprises 11 rounds. We have documented the reaction ratio of proteins and libraries for each round in Supplementary Table S3. The first-round reaction of the SELEX processes is presented in detail as follows.

#### Selection

First, we introduced 14.6 pmole of biotinylated thrombin to bind the protein to magnetic beads. We allowed it to react for 1 h with gentle rotational agitation within a total volume of 100 μl of PBS. After the reaction was completed, the thrombin-bead complex underwent another three washes, this time utilizing 1 ml of selection buffer (composed of 100 mM NaCl, 20 mM Tris–HCl, 2 mM MgCl_2_, 5 mM KCl, 1 mM CaCl_2_, 0.02% Tween 20, and pH 7.4). To initiate the selection reaction for proteins, we employed a library having the following sequence: 5′-CCA CTT CTC CTT CCA TCC TAA ACT CTC TCT CTC NNN NNN NNN NNN NNN NNN NNN NGA GAG AGA GAG AGT AGT TTG GAG GGT TGT CTG-3′. This library encompasses a total of 4^22^ random sequences, with each unique sequence having a copy number of 50, utilizing 1.46 nmole in the first-round reaction. Before commencing the selection using the library, we subjected it to denaturation at 95°C for 5 min and slowly cooling to 4°C (−0.1°C/s). We then added it to the thrombin-bead complex and subjected it to gentle rotational agitation within selection buffer for 2 h. Upon completing this reaction, the complex underwent five washes using selection buffer. Then, we added 50 μl of DNase/RNase-free water and carried out elution through a heat plate reaction at 95°C for 5 min. Of this, 3 μl was used for the PCR cycle regulation process using real-time qPCR, 25 μl was used for PCR for the next round, and the remainder was used for NGS library preparation.

#### Enrichment

To evaluate the evolution of selected unique sequences, we categorized the samples into three groups: Taq polymerase, SD polymerase, and a control group (Taq polymerase without PCR cycle regulation), and conducted PCR under the following conditions. We fine-tuned the PCR cycles for each round through PCR cycle regulation for the Taq polymerase and SD polymerase groups (Supplementary Fig. S1). In contrast, the control group determined the PCR cycle for each round based on the saturation Ct values obtained from real-time qPCR results. Initially, we performed real-time qPCR and PCR for the Taq polymerase group under the conditions of the cycling reaction, which comprised a pre-denaturation step at 94°C for 2 min, denaturation at 94°C for 20 s, annealing at 52°C for 10 s, extension at 72°C for 30 s, followed by a final extension at 72°C for 5 min, adjusting the PCR cycles for each round according to the PCR cycle regulation method. Likewise, the SD polymerase group underwent PCR and real-time qPCR under the conditions of the cycling reaction involving a pre-denaturation step at 92°C for 2 min, denaturation at 92°C for 10 s, annealing at 57°C for 10 s, and extension at 68°C for 30 s, followed by a final extension at 68°C for 5 min, mirroring the Taq group, and controlled the PCR cycles for each round following the PCR cycle regulation method. In contrast, the control group adhered to the PCR and real-time qPCR conditions of the Taq polymerase group but decided the PCR cycle for each round solely based on the saturation Ct values obtained from real-time qPCR, without employing the PCR cycle regulation method. Details of the PCR cycle adjustments for each group are listed in Supplementary Table S4. Gel analysis of PCR products from each SELEX round is shown in Supplementary Fig. S2.

#### Single-stranded DNA generation

To generate ssDNA from PCR product, an ssDNA generation procedure was employed. First, 100 µg of streptavidin-coated magnetic beads were washed twice with 500 µl of reaction buffer (composed of 20 mM Tris, 1 M NaCl, 1 mM EDTA, 0.0005% Triton X-100, and pH 7.5). Subsequently, biotinylated double-stranded DNA (dsDNA) and the streptavidin-coated magnetic beads were combined and incubated at room temperature for 1 h in reaction buffer. Following the reaction, the supernatant was discarded, and the beads were washed twice with washing buffer (composed of 20 mM Tris, 300 mM NaCl, 5 mM KCl, 0.01% Triton X-100, and pH 7.5). Then, 0.15 N NaOH was added, and the mixture was incubated for 6 min. The supernatant containing the sense strand DNA (single stranded DNA, enriched aptamer pool) was collected and subjected to purification using the Monarch PCR & DNA cleanup kit (NEB). The purification method employed for ssDNA generation was the same as for the PCR purification process. The purified ssDNA quantified with Qubit^™^ ssDNA assay kit was used in the following round. The quantities of ssDNA used in each SELEX round are summarized in Supplementary Table S3.

### High-throughput sequencing

#### Library preparation

In this study, we address the challenges associated with the amplification of nucleic acids characterized by complex structures due to the properties of polymerase. Using polymerases with potential issues in amplifying nucleic acids of diverse structures may lead to complications in result analysis. Consequently, to mitigate such concerns in NGS library preparations, we uniformly applied the SD polymerase, confirmed for its capability to effectively amplify nucleic acids of most structures across all experimental groups. To apply SD polymerase during library preparation procedure, we prepared the NGS library manually. NGS library preparation for the MGI sequencing platform was performed for the 2nd, 5th, 8th, and 11th round eluents, which underwent appropriate amplification through PCR cycle regulation with a phosphorylated primer pair. The library preparation was conducted in accordance with the instructions provided in the MGIEasy Universal DNA library prep kit manual. Briefly, the amplified aptamer pool was modified at the 3′ end to introduce a dATP as a sticky end. Subsequently, the dTTP tailed adapter sequence was ligated to both ends of the DNA fragments. The resulting ligation product underwent amplification for 10 cycles, following the PCR conditions: the reaction involved a pre-denaturation step at 92°C for 2 min, cycles of denaturation at 92°C for 10 s, annealing at 60°C for 10 s, and extension at 68°C for 30 s, followed by a final extension at 68°C for 5 min. This amplification step was performed accordingly. The PCR product was subsequently submitted to the LAS (Seoul, Republic of Korea) for further processing, including the single-strand circularization process and subsequent sequencing.

The amplified libraries were sequenced using DNBSEQ-T7 (MGI) with a read length of 100 bp; 12.1 gigabase paired-end sequencing data were generated per sample on an average. Base quality was confirmed using FASTX-Toolkit (ver.0.0.13), and given that the average Phred quality score in all FASTQ files was 34.88 ± 0.14, the entire raw read sequence was used for filtering without trimming (%base > Q30 = 93.34%).

#### Data processing for extraction of N22 sequences

NGS libraries were paired-end sequenced and Reads 1 and 2 were separately processed to extract core N22 sequences as per the steps described in Supplementary Fig. S3A. The structure of the aptamer library contains fixed sequences defined as constant region and stem. Therefore, through serial pattern matching using such fixed sequences, a pool of random N22 sequences was recovered for each round. First, raw reads containing left and right constant region sequences were selected. Raw reads containing both constant regions were defined as matching reads. Considering the NGS error rate, a mismatch of one base was allowed in each constant region, resulting in matching rate of 90.16 ± 1.72% to the number of raw reads. As the aptamer was a single-stranded DNA and paired-end NGS libraries were not strand-specifically sequenced, matching reads in paired-end reads were reverse complemented when they encoded complementary DNA sequences of aptamers. Next, subsequences flanked between 8-mer sequences of left and right constant regions (5′-TCCTAAAC-3′ and 5′-GAGTAGTT-3′) were extracted as Flanking N-mers. Among them, 22-mer random sequences between two stem sequences, 5′-TCTCTCTCTC-3′ and 5′-GAGAGAGAGA-3′, were extracted. The frequency of N22 sequences was counted, and N22 sequences having a frequency >2 were used for further analysis.

#### Analysis of N22 sequences for aptamer candidate selection

All pairwise overlapping analysis was performed to confirm the reproducibility of the N22 sequences top ranked for frequency. Total sum of frequencies of the overlapping N22 sequences between rounds or batches were normalized by the read counts of the corresponding pair of comparison. Reproducible N22 was defined as the overlapping N22 sequences in the same round of different batches. To confirm the molecular evolution of N22 sequences, diversity indices were calculated using the vegan v2.6-4 R package [22]. Since estimation of alpha diversity can be biased depending on the size of the subsamples, N22 sequences were enumerated according to various cutoff for frequency: N22 sequence of which frequency more than mean, 3rd quartile (Q3), or within top 10%. To identify criteria for discovering high-affinity TBA sequences, consensus motif in the top-ranked reproducible N22 sequences were analyzed using MEME Suite (ver.5.5.4) [23]. To track changes in frequency rank of the motif-containing candidate sequences during SELEX, the rank of the sequence for each round was converted to percentile rank by dividing it by the total number of unique N22 sequences for the corresponding round.

#### Secondary-structure analysis

The structural landscape of the aptamer pool was evaluated through secondary-structure predictions using RNAfold (version 2.6.4) with DNA parameters and the G-quadruplex prediction option enabled [24]. The predicted structures, represented in dot-bracket notation, were categorized into two mutually exclusive groups based on their folding patterns. Simple structures exhibit a continuous stem-loop pattern in which flanking base-pairing encloses the random region. Complex structures contain additional secondary motifs within the random region that deviate from the canonical stem-loop architecture. The Complex group was further divided by the number of internal base pairs (2–9) within the random region.

### Binding affinity analysis

Real-time interaction analysis was performed by surface plasmon resonance (SPR) on an IMSPR-ProX system (ICLUEBIO, Seongnam, Republic of Korea). Thrombin was used as the target, and the 15-mer thrombin-binding aptamer (TBA, TGT_T) [25] was analysed as a reference alongside the SD-SELEX and Taq-SELEX candidate sequences (Supplementary Table S1). For each aptamer, sensorgrams were recorded across a series of concentrations and fitted with a 1:1 Langmuir binding model to determine the dissociation constant (K_D_). Each K_D_ value is reported as the mean ± SD of three independent measurements (*n* = 3).

Thrombin was diluted in 10 mM sodium acetate buffer at pH 5.0 and immobilized on a research-grade carboxylic (COOH) sensor chip (HC1000, ICLUEBIO) using EDC [1-ethyl-3-(3-dymethylaminopropyl)carbodiimide]/NHS (N-hydroxysuccinimide). The remaining succinimide esters on the sensor chip were blocked and de-activated using ethanolamine. A total of 4506 resonance units (RU) of immobilized proteins was obtained. Sensor preparation and interaction analyses were performed at 25°C in selection buffer (composed of 100 mM NaCl, 20 mM Tris–HCl, 2 mM MgCl_2_, 5 mM KCl, 1 mM CaCl_2_, 0.02% Tween 20, pH 7.4). The oligonucleotides were dissolved in the selection buffer at 2 μM, annealed (as described in the SELEX section), and injected over the surfaces at various concentrations. All solutions were sequentially injected over the sensor surface for 3 min at 30 μl/min with a 5 min dissociation time beginning with the lowest concentration from 12.5 to 200 nm or 62.5 to 1000 nm. After each run, the surface was regenerated using 5 mM aqueous NaOH for 15 sec at 30 μl/min. The RU was measured throughout all steps with iMSPR ProX (ICLUEBIO, Republic of Korea) using iMSPR software. The data were analyzed with TraceDrawer (ver.1.9.2, Uppsala, SWEDEN) by curve fitting using a 1:1 binding model.

### Parallel SELEX against VEGF-165

To test whether the amplification-bias effect is specific to thrombin, a parallel SELEX was carried out against VEGF-165 (ACROBiosystems) under the conditions used for thrombin, with SD or Taq polymerase as the only difference between the two selections. Eluents from rounds 2, 5, 8, and 11 were sequenced and processed through the pipeline described above. Retention of a previously reported VEGF-binding motif (5′-GGGGGTGGACGG-3′) was quantified by counting motif-containing N22 sequences in each round, both in the entire pool and within the top 10% of reads by frequency, for each polymerase. Two motif-containing sequences recovered from the SD-SELEX pool were synthesized, and their binding to VEGF-165 was examined by SPR under the conditions described above, except that the values from the three measurements are reported individually rather than as a mean.

### Circular dichroism

Circular dichroism (CD) spectra were recorded from 320 to 220 nm using a rectangular quartz cell with a 10 mm path-length at 25°C on a J-1100 circular dichroism spectrophotometer (JASCO Ltd., Japan). For the CD experiment, various aptamers (2 μM) was dissolved in 1× selection buffer (100 mM NaCl, 20 mM Tris–HCl, 2 mM MgCl_2_, 5 mM KCl, 1 mM CaCl_2_, 0.02% Tween 20, and pH 7.4). The reported spectrum represents the average of three scans at a scan rate of 100 nm/min with a bandwidth of 1 nm and standard sensitivity. The background data of the buffer solution was automatically subtracted from the CD data. The baseline correction was done and smoothened by Savitzky–Golay method.

## Results

### Superior amplification efficiency of SD polymerase for structured templates

One of the challenges in conventional SELEX lies in the PCR amplification bias introduced by Taq polymerase when processing structurally complex templates. In contrast, SD polymerase can efficiently amplify complex structured ssDNA templates owing to its strand-displacement capability (Fig. 1A). The SD polymerase used in this study possesses strong strand-displacement activity, enabling continuous DNA synthesis while displacing downstream duplex regions. Unlike conventional PCR polymerases such as Taq, which tend to stall at stable secondary structures, strand-displacement polymerases can traverse structured templates without the need for accessory helicase activity. According to the manufacturer’s specifications, the enzyme lacks 5′→3′ exonuclease activity and is optimized for structure-tolerant amplification (Supplementary Table S5). This biochemical property is expected to reduce amplification bias against highly structured sequences, such as G-quadruplex-forming motifs, compared to conventional polymerases that exhibit limited strand-displacement capability (Fig. 1A). To systematically evaluate this limitation and validate our proposed solution, we first compared the amplification capabilities of SD and Taq polymerases using a panel of synthetic oligonucleotides with varying structural complexities (Supplementary Table S1).

**Figure 1.**
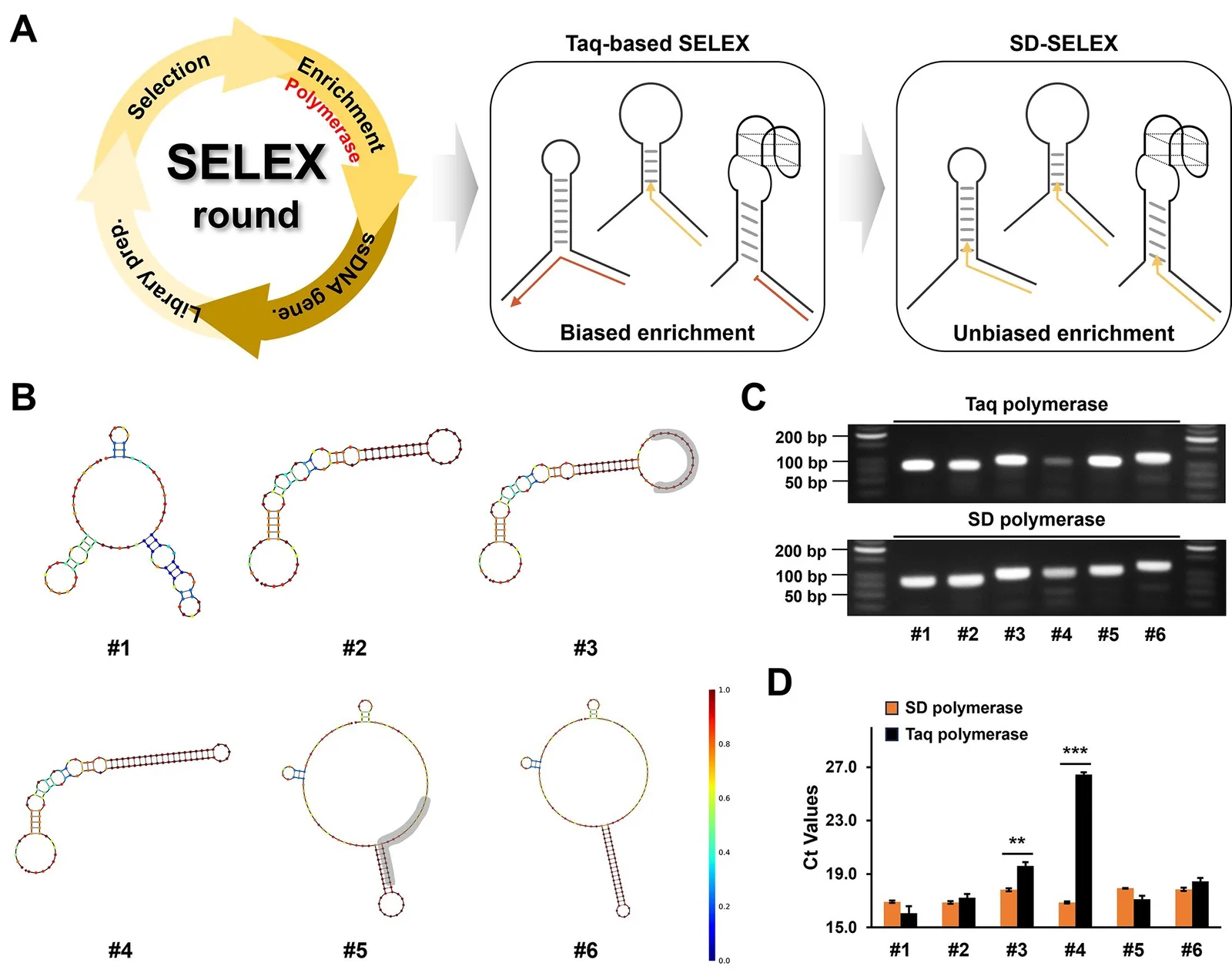
Comparison of differences caused by polymerases in the enrichment stage during the SELEX process. (**A**) Conventional enrichment method using Taq polymerase experiences amplification stoppage because of the diverse structures of templates or phenomena like “jumping polymerase,” resulting in biased amplification. In contrast, SD polymerase consistently amplifies most templates regardless of their structural diversity, minimizing variation between amplified templates in the aptamer library. We have prepared Templates #1 to #6, and their secondary structures correspond to those shown in panel (**B**) (predicted using NUPACK version 4.0); templates #3 and #5 additionally contain G-quadruplex-forming sequences, and the corresponding G-tracts are shaded in panel (B). Because the base-pairing prediction used here does not model G-quadruplexes, these segments are displayed as unpaired regions. Template sequences are listed in Supplementary Table S1. The amplification tests on the templates were conducted using SD polymerase and Taq polymerase. The results were compared using (**C**) 2% agarose gel electrophoresis and (**D**) real-time qPCR. Data are presented as the mean ± SD of three replicates (*n* = 3). Statistical significance was determined using a two-tailed paired Student’s t-test. **P < .01; ***P < .001.

We designed a series of artificial templates based on sequences from previous structural studies, incorporating diverse secondary structures including hairpins, stem–loops, and G-quadruplexes (Fig. 1B). Template #1 served as a control with no predicted secondary structure, while templates #2, #4, and #6 contained hairpin structures with high ΔG values due to GC-rich stem regions. Templates #8 and #9 featured lower ΔG hairpins with AT-rich stems, and templates #3, #5, #7, and #9 incorporated potential G-quadruplex-forming sequences in their variable regions. Templates #1–#6 are compared in Fig. 1, whereas templates #7–#9 were analysed separately and are presented in Supplementary Fig. S4.

Comparative analysis through agarose gel electrophoresis and real-time qPCR revealed differences between the two polymerases across six structurally diverse templates (#1–6) (Fig. 1C and D). Taq polymerase showed reduced amplification efficiency for structured templates, with lower band intensities and higher Ct values; the statistical significance of the Ct difference for each template is indicated in Fig. 1D (two-tailed paired Student’s t-test, *n* = 3). In contrast, SD polymerase maintained stable and efficient amplification across all template types, producing minimal by-products and consistent yields.

The most substantial difference was observed with template #4, which showed severely impaired amplification with Taq polymerase (Ct = 26.5) versus efficient processing by SD polymerase (Ct = 17.1). While SD polymerase maintained consistent amplification efficiency across all templates, detailed examination of gel electrophoresis revealed that template #4 presented increased complexity. Despite achieving low Ct values comparable to other templates, #4 showed moderately reduced band intensity and minor sub-target products in SD polymerase-based amplification compared to simpler templates. This suggests that highly complex structural templates may occasionally induce limited “polymerase jumping” events in SD polymerase systems, though significantly less than the extensive template switching observed with Taq polymerase [26]. Notably, SD polymerase achieved functional amplification under conditions where Taq polymerase showed substantial performance degradation, demonstrating SD polymerase’s superior tolerance for structured substrates. This structural bias becomes particularly problematic in SELEX, where multiple selection rounds and PCR cycles exponentially compound these efficiency differences, potentially excluding high-affinity aptamer candidates based on structural complexity rather than binding performance.

Templates #7–#9 were analysed separately under the same conditions (Supplementary Fig. S4). For these templates, agarose gel electrophoresis and qPCR showed no substantial difference between Taq and SD polymerase: only small differences were observed for templates #7 and #9, and template #8 was amplified comparably by both enzymes. The advantage of SD polymerase is therefore template-dependent and is most pronounced for templates in which a stable stem is combined with an additional structural element, as in templates #3 and #4. To ensure our findings were not limited to Taq polymerase, we evaluated several high-fidelity enzymes designed for challenging templates, including Platinum^™^ SuperFi II, Q5^®^ Hot Start, Phusion Hot Start II, and OneTaq^®^ polymerases (Supplementary Fig. S5).

To further determine whether these sequence-dependent amplification differences were attributable to K⁺-mediated stabilization of G4 structures, we examined templates #3, #5, #7, and #9 using NEB Taq DNA polymerase in a well-defined buffer system across a range of KCl concentrations, including a KCl-free condition. Endpoint PCR and qPCR analyses showed that KCl modulated amplification efficiency in a sequence-dependent manner (Supplementary Fig. S6). Templates #5, #7, and #9, which possess G4-forming potential, exhibited modest changes in amplification efficiency depending on the KCl concentration, consistent with the established role of K⁺ in stabilizing G4 structures and potentially impeding polymerase progression [27, 28, 29]. However, the magnitude and direction of these effects varied among the templates. Notably, template #3 remained inefficiently amplified under all KCl conditions, indicating that its poor amplification could not be explained solely by K⁺-dependent G4 stabilization. These findings suggest that buffer composition, including K⁺ concentration, can modulate the amplification of structured sequences but is not the sole determinant of sequence-dependent amplification bias. Rather, the intrinsic properties of the polymerase, together with broader sequence- and structure-specific features of the template, appear to have a more substantial influence on amplification performance under the conditions examined. This interpretation is further supported by the relatively uniform amplification achieved by SD polymerase across structurally diverse templates, in contrast to the greater sequence-dependent variability observed with Taq polymerase.

These results establish the mechanistic foundation for our SD-SELEX approach. Therefore, by replacing the amplification enzyme, we can directly address the structural bias that has historically limited aptamer discovery to simpler, more easily amplified sequences.

### Enhanced library preservation and reproducibility in SD-SELEX

Having established SD polymerase’s superior amplification efficiency for individual structured templates, we next evaluated whether this advantage translates to maintaining library complexity and structural preservation throughout iterative SELEX rounds. We conducted parallel SELEX experiments targeting thrombin using identical N22 random libraries and selection conditions, differing only in the choice of amplification polymerase.

To analyze library composition in each round, N22 random sequences were recovered from NGS reads by serial pattern matching using the constant and stem regions. The proportion of N22 sequences relative to total raw reads decreased as the SELEX rounds progressed, as shown in Supplementary Fig. S3B. This declining recovery rate reflects the accumulating structural complexity that challenges polymerase processivity during iterative SELEX rounds, with the extent of decline being markedly different between SD and Taq polymerase systems as shown below.

Complementary analysis of flanking region lengths quantified the extent of sequence truncation (Fig. 2A and B). Raw sequencing reads from SD-SELEX consistently displayed well-aligned, intact aptamer structures (Constant-Stem-N22), while Taq-SELEX produced increasingly fragmented and aberrant sequences as rounds progressed (Fig. 2A and Supplementary Fig. S7). Quantitative assessment of complete structural preservation showed that SD polymerase maintained significantly higher proportions of intact sequences: a 1.5-fold advantage in round 2 expanding to a remarkable 37-fold difference by round 11 (Fig. 2B).

**Figure 2.**
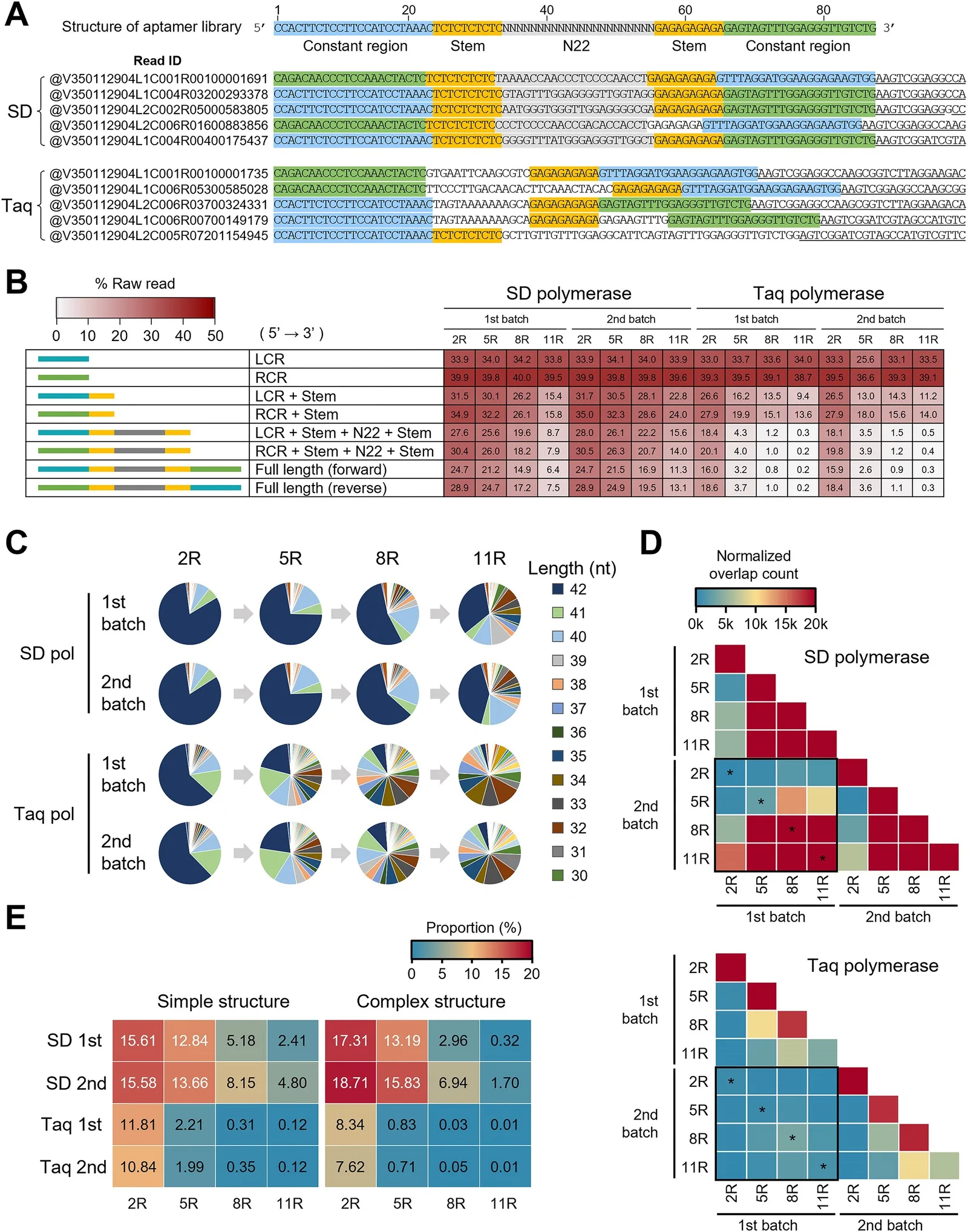
Strand-displacement activity of DNA polymerase enabled an intact amplification of aptamer sequences. (**A**) NGS reads from 8R product of the first batch were representatively visualized to confirm the structure of aptamer library. A read per 20 M reads was selected from merged paired-end reads. Underlined letters refer to sequencing adaptor. (**B**) Quantification of matching rate in raw reads containing constant and stem sequences in 5′–3′ order. (**C**) Proportion of the length of flanking N-mer. (**D**) Heat map showing all pairwise overlapping between top 10% ranked N22 sequences by frequency. The number of overlapped sequences were normalized by the total number of reads per million as per the following equation: [(Sum of frequencies of overlapped N22)/(Sum of total read of the corresponding pair of comparison)] × 1 million. Diagonal asterisks within the black-lined square indicate the overlapping N22 sequences for the same round between batches. (**E**) Preservation of secondary-structure content during SELEX. Proportion of recovered N22 reads predicted to form Simple (left) or Complex (right) secondary structures in SD- and Taq-SELEX at rounds 2, 5, 8, and 11 (two batches per condition). Structures were predicted with RNAfold (DNA parameters, with the G-quadruplex option enabled) and classified as described in the “Materials and Methods”. Abbreviations: LCR, left constant region; RCR, right constant region; N22, 22-nucleotide random region; 2R, 5R, 8R, and 11R, selection rounds 2, 5, 8, and 11, respectively; nt, nucleotides.

The degradation of library quality in Taq-SELEX correlated with increased frequency of “stem hopping” events (polymerase jumping), where the enzyme switches between templates at structured regions (Supplementary Fig. S8). This phenomenon not only reduces the representation of intended sequences but actively introduces artificial variants that can confound subsequent selection rounds. The preferential amplification of shorter products in PCR further exacerbates this issue, creating a progressive bias toward truncated, less structured sequences.

Analysis of flanking region lengths provided additional evidence of structural preservation differences (Fig. 2C). While SD-SELEX maintained the expected 42-nucleotide flanking regions as the majority population through round 11, Taq-SELEX showed progressive erosion, with intact sequences becoming a minority after round 2. By round 11, SD polymerase preserved nine-fold more complete structures compared to Taq polymerase.

Critically, the structural preservation advantages of SD polymerase translated into substantially improved experimental reproducibility (Fig. 2D). Batch-to-batch consistency, measured by overlap of the top 10% most frequent sequences, progressively improved throughout SD-SELEX rounds, while Taq-SELEX showed strong batch effects with limited inter-batch concordance. By round 11, SD-SELEX demonstrated 65-fold higher reproducibility than Taq-SELEX (Supplementary Table S6), indicating that structural biases not only reduce library quality but also compromise the reliability of aptamer selection outcomes.

Consistent with these sequence-level preservation patterns, secondary-structure analysis further revealed distinct structural evolution between the two SELEX systems (Fig. 2E). Predicted structures were classified as Simple or Complex according to the extent of secondary-structure formation within the N22 random region (Supplementary Fig. S9). SD-SELEX maintained a greater proportion of sequences with complex secondary structures throughout the selection rounds, whereas Taq-SELEX progressively shifted toward sequences with simpler predicted structures. These results further support that SD polymerase reduces structure-dependent amplification bias and preserves structurally diverse sequences during iterative SELEX.

To further examine the preservation of structurally complex sequences, the Complex group was subdivided according to the number of internal base pairs formed within the N22 random region (Supplementary Fig. S10). SD-SELEX showed a gradual reduction in sequences with increasing levels of internal pairing across selection rounds, whereas Taq-SELEX exhibited a markedly more rapid depletion of these sequences. This difference became increasingly apparent as the degree of internal base pairing increased.

These findings demonstrate that SD polymerase’s structural preservation capacity not only maintains library quality but ensures reproducible evolution of functionally superior aptamer candidates. In contrast, conventional Taq polymerase-based amplification progressively degrades structured libraries toward simple, easily amplified sequences regardless of their functional properties, fundamentally limiting the accessible aptamer sequence space.

### Preservation and evolution of G-quadruplex motifs in SD-SELEX

With library integrity preserved, we investigated whether SD-SELEX could maintain and evolve functionally relevant structured motifs that are typically lost in conventional protocols. Diversity analysis confirmed that both SD and Taq polymerase systems achieved effective sequence convergence by round 8, with diversity indices reaching comparable plateaus, indicating that the observed differences stem from which sequences are selected rather than the efficiency of the selection process itself (Fig. 3A).

**Figure 3.**
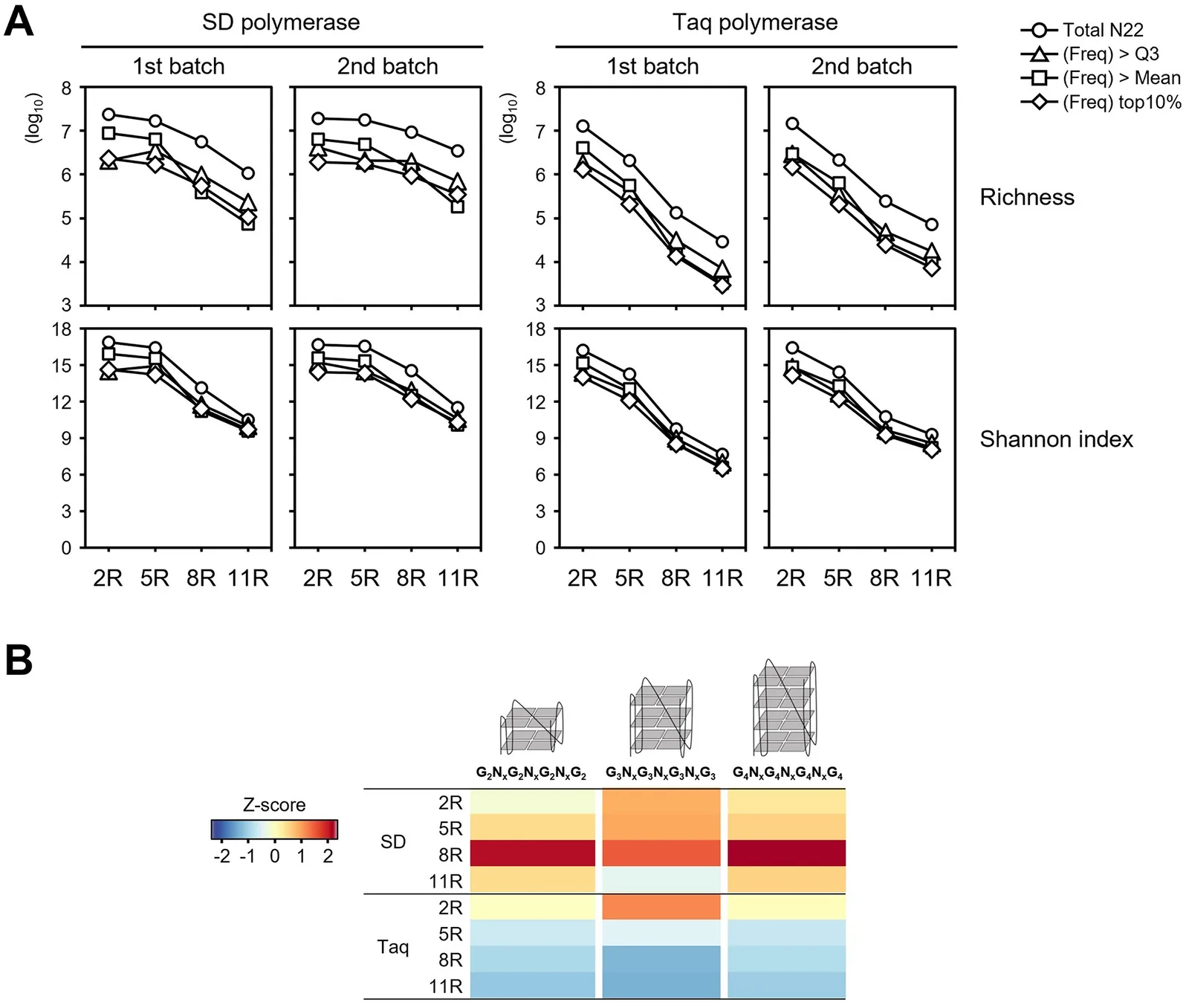
N22 sequences containing G-quadruplex motif were enriched using SD polymerase. (**A**) Diversity analysis of N22 sequences, plotted on a log_10_ scale. Three cutoffs were applied to select high-frequency N22 sequences from total sequences (°); N22 sequences whose frequencies were higher than 3rd quartile (△), mean (□), top 10% (◇) in the corresponding batch and round. (**B**) Heat map showing the proportion of N22 sequences containing G-quadruplex motifs. Potential G-quadruplex motifs were defined based on the number of guanine tetrads as follows: G-quadruplex with 2-tetrad (G_2_N_x_G_2_N_x_G_2_N_x_G_2_, x ≥ 1), 3-tetrad (G_3_N_x_G_3_N_x_G_3_N_x_G_3_, x ≥ 1), and 4-tetrad (G_4_N_x_G_4_N_x_G_4_N_x_G_4_, x ≥ 1). The number of N22 sequences containing potential G-quadruplex motifs was counted from the total N22 sequence. Then, the number of sequences was divided by the total number of raw reads to calculate the proportion, and the proportions were normalized as Z-Score within G-quadruplex motif type.

Consensus motif analysis of the top 10% most frequent sequences showed that both polymerase systems converged on similar GGTTGG-containing motifs (Supplementary Fig. S11), indicating that the most abundant sequences are not qualitatively different between the two selections. The systems differed instead in how much of the population carried such motifs. In SD-SELEX, the proportion of sequences carrying two-tetrad G-quadruplex motifs, the class that includes the GGTTGG-loop-GGTTGG arrangement, increased across rounds (Fig. 3B). In contrast, Taq-SELEX showed G-rich sequences after round 2 but did not maintain them, the pool instead converging on simpler, linear sequences more amenable to standard PCR amplification (Fig. 3B).

Quantitative tracking of potential G-quadruplex motifs throughout the selection process revealed the extent of structural bias in conventional SELEX (Fig. 3B and Supplementary Fig. S12). SD-SELEX showed steady enrichment of G4-forming sequences through round 8, consistent with positive selection for thrombin-binding function. Conversely, Taq-SELEX exhibited dramatic depletion of G4 motifs after round 2, despite thrombin’s well-established preference for G-quadruplex aptamers. Analysis of sequences that disappeared after early rounds confirmed that G4-containing sequences were disproportionately lost in Taq-SELEX (∼2-fold higher dropout rate compared to SD-SELEX; Supplementary Fig. S13), strongly implicating amplification bias rather than functional selection pressure.

To validate this interpretation, we specifically monitored the 15-nucleotide thrombin-binding aptamer (TBA15, 5′-GGTTGGTGTGGTTGG-3′), a well-characterized high-affinity G-quadruplex aptamer (Supplementary Fig. S14). SD-SELEX showed progressive enrichment of sequences containing this motif through round 8, while Taq-SELEX failed to enrich TBA15-containing sequences despite identical selection conditions. Intriguingly, within the SD-SELEX top performers, TBA15-containing sequences gradually decreased in relative frequency, suggesting that competing G-quadruplex variants with superior binding properties were being discovered and outcompeting the known reference sequence.

These results demonstrate that, in the present thrombin selection system, G4-containing sequences show markedly different evolutionary trajectories depending on the amplification polymerase. Together with the broader secondary-structure analysis described above, the G4 results provide a representative functional example of how structure-dependent amplification can influence sequence retention and enrichment during iterative SELEX.

### Discovery of novel high-affinity G-quadruplex thrombin aptamers

SD-SELEX preserved G-quadruplex motifs throughout selection, enabling discovery of novel high-affinity aptamers. Analysis of N22 sequences revealed that 15-mer (GGTTGG-NNN-GGTTGG) and 14-mer (GGTTGG-NN-GGTTGG) architectures were the most abundant consensus motifs (Supplementary Fig. S15). We therefore extracted top-ranked sequences containing these loop configurations for detailed characterization. Profiling of loop sequences within the GGTTGG-loop-GGTTGG motif identified six dominant variants, including the known TBA15 sequence and five previously uncharacterized architectures (Fig. 4A). All five novel variants reached higher frequencies than TBA15 by round 8, indicating that they were enriched at least as efficiently as the known aptamer under these selection conditions.

**Figure 4.**
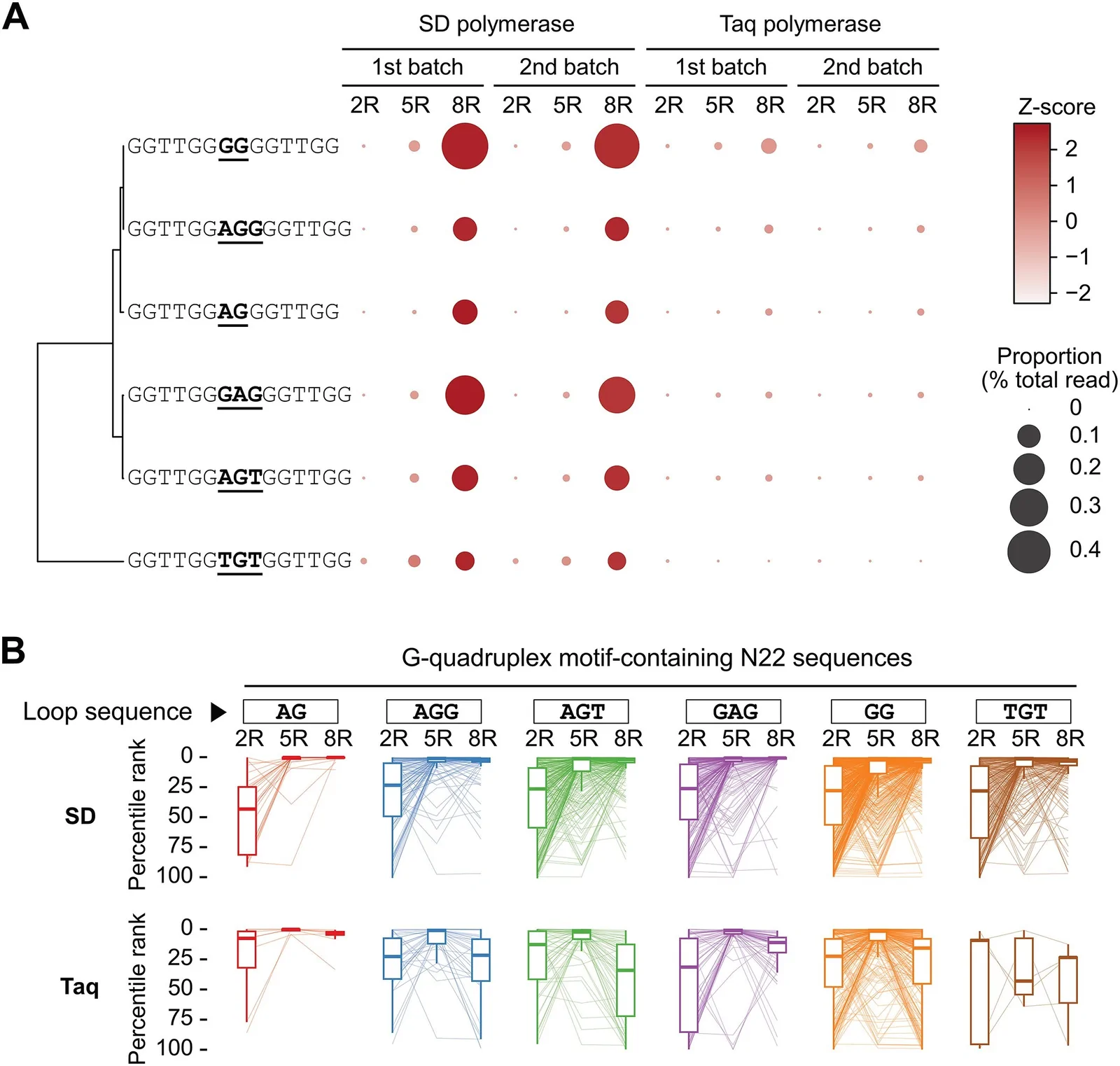
High-affinity TBA candidates were identified based on evolution of N22 sequence containing G-quadruplex motif (GGTTGG-loop-GGTTGG). (**A**) The number of motif-containing N22 sequences was calculated as a proportion to the number of raw reads (represented as a size of circles). Difference in sequence proportion between SD and Taq polymerases was normalized as Z-Score. Underlined bold letters within G-quadruplex motif refer to loop sequences. Rows were ordered using hierarchical clustering based on the sequence proportion matrix. (**B**) Evolution of G-quadruplex motif-containing N22 sequences was tracked based on rank percentiles by frequency. Percentile rank of the sequence for each round was calculated as following equation: [(Frequency rank of the motif-containing N22 sequence in each round)/(Total number of unique N22 sequences of the corresponding round)] × 100. Each line of the plots represents a respective N22 sequence containing the indicated G-quadruplex motif. The boxplot shows the rank distribution of G-quadruplex motif-containing N22 sequences for each round.

Evolutionary trajectory analysis confirmed progressive enrichment of these variants throughout SD-SELEX rounds, with frequency rankings steadily improving from random distribution to top-tier positions (Fig. 4B and Supplementary Fig. S16). In contrast, parallel Taq-SELEX experiments showed stochastic fluctuations without coherent enrichment patterns, demonstrating that structured motif evolution requires compatible amplification chemistry.

Positional analysis within the N22 variable regions revealed that SD-SELEX favored centered or slightly offset G-quadruplex placement (Supplementary Fig. S17), likely optimizing target accessibility while maintaining structural stability. Taq-SELEX showed highly dispersed positioning patterns for the few sequences that survived initial rounds, indicating that amplification bias overwhelms functional selection pressure.

Surface plasmon resonance analysis was used to compare the aptamers recovered from the two selections (Table 1 and Supplementary Fig. S18). Candidates were selected based on frequency ranking, batch-to-batch reproducibility, and motif representation. TGT_T (corresponding to TBA15) served as a positive control with K_D_ = 196 ± 75 nm. The strongest SD-SELEX candidates, GG_8F_R and AGT_8F_R, bound with K_D_ values of 75 ± 11 nm and 96 ± 17 nm, respectively, which is comparable to, or at most three-fold better than, the reference aptamer; these differences did not reach statistical significance. The two Taq-SELEX candidates examined bound more weakly (K_D_ = 494 and 814 nm; Supplementary Fig. S19A), and the K_D_ values of the SD-SELEX and Taq-SELEX candidates did not overlap (Mann–Whitney U test, U = 0, *P* = .019), although the number of Taq-SELEX candidates available for comparison was small. To assess specificity, both the SD-SELEX candidates and the three Taq-SELEX candidates were tested against the unrelated proteins MUC1 and VEGF165 and showed only background-level binding (Supplementary Figs S20 and S21), so the two selections did not differ in specificity.

**Table 1.** K_D_ analysis of selected aptamer candidates

Name	k_on_ (×10⁴ M⁻¹ s⁻¹)	k_off_ (×10⁻³ s⁻¹)	K_D_ (×10⁻^7^ M)
TGT_T	5.20 ± 2.68	8.98 ± 1.26	1.96 ± 0.75
TGT_8F	5.43 ± 1.32	8.14 ± 0.53	1.56 ± 0.38
AG_8F	2.36 ± 0.10	9.39 ± 0.01	3.98 ± 0.17
AG_8F_R	3.41 ± 1.10	9.88 ± 1.41	3.07 ± 0.85
AGG_8F	4.22 ± 2.57	9.30 ± 0.67	2.70 ± 1.21
AGG_8F2	5.61 ± 0.59	10.31 ± 0.78	1.86 ± 0.30
AGG_8F_R	2.93 ± 0.83	6.42 ± 0.33	2.30 ± 0.77
AGT_8F	6.99 ± 2.56	10.35 ± 0.73	1.64 ± 0.65
AGT_8F_R	10.01 ± 2.46	9.38 ± 1.02	0.96 ± 0.17
GAG_8F	6.08 ± 2.03	5.78 ± 0.65	1.01 ± 0.28
GAG_5F	3.53 ± 1.03	7.64 ± 0.65	2.24 ± 0.47
GAG_8F_R	3.55 ± 2.26	8.10 ± 3.43	2.78 ± 1.17
GG_8F	3.82 ± 1.07	5.68 ± 0.40	1.56 ± 0.38
GG_8F_R	8.13 ± 1.90	5.99 ± 0.66	0.75 ± 0.11

*Data are presented as the mean ± SD of three independent measurements (*n* = 3). K_D_ values were calculated individually from k_off_/k_on_ for each measurement.

Circular dichroism spectroscopy revealed the structural diversity achieved through SD-SELEX (Supplementary Fig. S22). Selected aptamers adopted various topologies including parallel, antiparallel, and mixed-type G-quadruplex conformations, often combined with stem-loop elements from the hairpin backbone. This structural diversity, maintained by unbiased amplification, provides multiple architectural solutions for target recognition while preserving the quadruplex core responsible for thrombin binding.

These findings demonstrate that the preservation of structured sequence populations during SD-SELEX can facilitate the recovery and evolution of functionally relevant G4 variants in the thrombin selection system. Together with the broader secondary-structure analysis, these results support the ability of SD-SELEX to maintain a more structurally diverse sequence space during aptamer selection.

### Reduced amplification bias on a second target, VEGF-165

To test whether the reduction of amplification bias by SD polymerase is specific to thrombin, we performed a parallel SELEX against VEGF-165, a target for which both G4 and non-G4 aptamers have been reported [30, 31, 32]. Both selections were sequenced at rounds 2, 5, 8, and 11 and analyzed with the same pipeline.

The two arms diverged as they had for thrombin. SD-SELEX retained the designed full-length library architecture across rounds, whereas Taq-SELEX lost it from round 2; by round 11 the full-length fraction was 89-fold higher under SD (Fig. 5A). N22 recovery declined gradually under SD but sharply under Taq (Supplementary Fig. S23).

**Figure 5.**
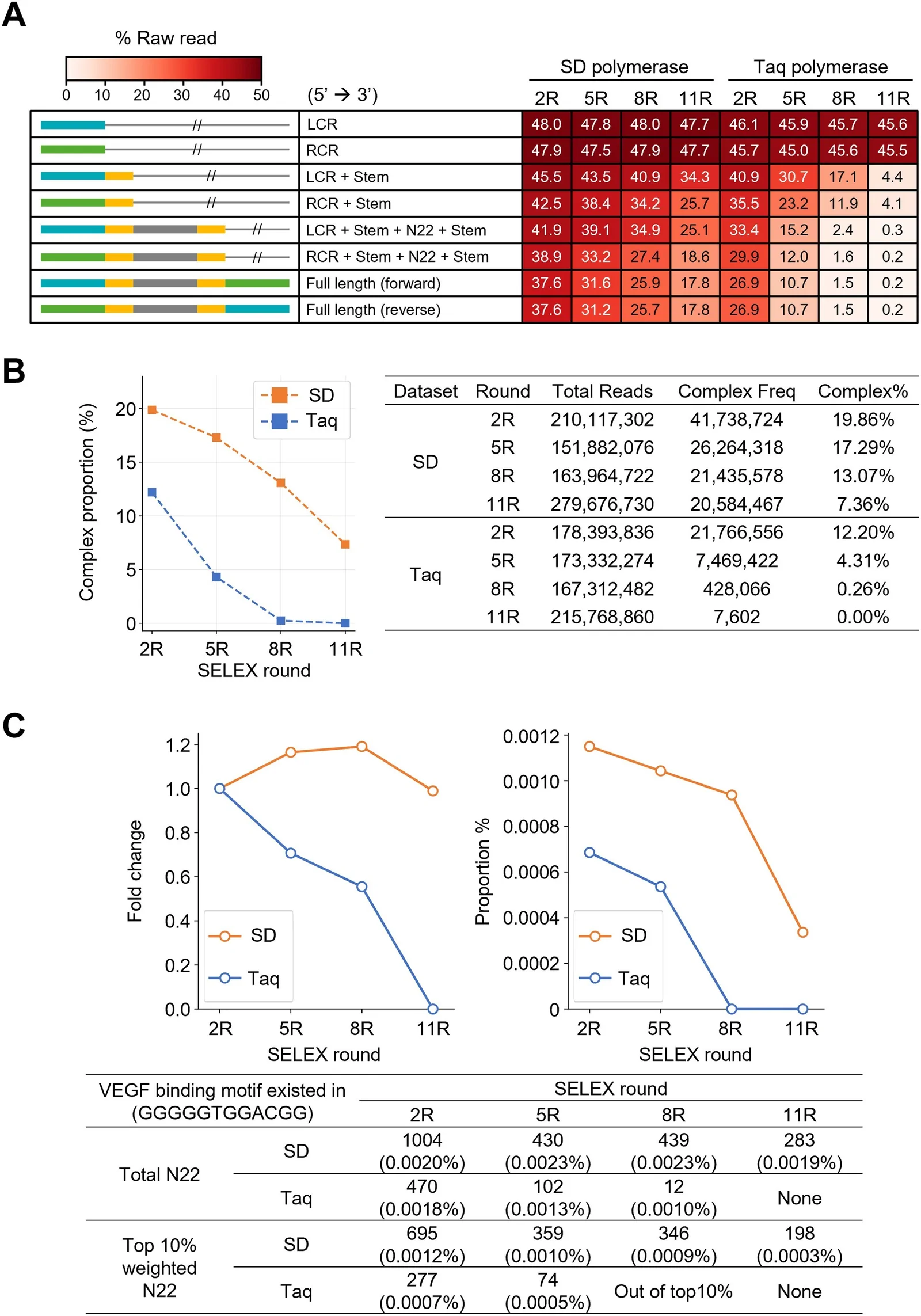
Reduced amplification bias on a second target, VEGF-165. (**A**) Retention of the designed library architecture: proportion of raw reads (%) containing each library component (constant regions, stems, N22, full-length) at rounds 2, 5, 8, and 11 for SD- and Taq-SELEX; the full-length fraction at round 11 is 89-fold higher under SD (17.8% versus 0.2%). (**B**) Proportion of reads forming complex secondary structures across rounds, with the underlying read counts (table); at round 11, the complex fraction is 7.36% under SD and 0.0035% under Taq (∼2090-fold). (**C**) Retention dynamics of the VEGF-binding motif across selection rounds. Counts, proportions, and depth-normalized fold changes (relative to Round 2) of N22 sequences containing the previously reported VEGF-binding motif (GGGGGTGGACGG) in the full pool and top 10% of reads. “None” indicates that no motif-containing sequence was detected, and “Out of top10%” represents that motif-containing sequences were present in the pool but absent from the top 10% of reads. The motif is retained under SD conditions through Round 11, whereas it is lost under Taq conditions.

Secondary-structure content followed the same pattern. G-quadruplex-containing reads were retained under SD and depleted under Taq after round 2 (Supplementary Fig. S24), and reads forming complex structures were maintained far better under SD, exceeding Taq by roughly three orders of magnitude at round 11 (7.4% versus 0.004%; Fig. 5B).

A previously reported VEGF-binding motif (GGGGGTGGACGG) [31] was retained in the SD pool through round 11 and remained within the top 10% of reads, but was progressively lost under Taq and undetectable by round 11 (Fig. 5C). Two motif-containing sequences recovered from the SD pool bound VEGF-165 in a concentration-dependent manner by SPR (Supplementary Fig. S25). Responses were small, and the values obtained from three independent measurements varied by up to 15-fold, so they are reported for each replicate rather than as a mean dissociation constant. A known functional motif, independent of G-quadruplex formation, was therefore preserved by SD-SELEX and lost by Taq-SELEX on a second target.

## Discussion

This study experimentally demonstrates that strand-displacement SELEX (SD-SELEX) significantly improves the stability of preservation and evolution of structurally complex aptamers, including G-quadruplexes (G4s), across selection rounds. We quantitatively confirmed structure-dependent dropout under Taq polymerase-based amplification. We showed that replacing the amplification engine with a SD polymerase, characterized by vigorous 5′–3′ strand-displacement activity and the absence of 5′–3′ exonuclease activity, thereby “pushing” downstream strands without cleavage, allowed high-affinity G4-bearing candidates to be stably retained and evolved. These results indicate that structural compatibility during amplification can act as a bottleneck in SELEX alongside intrinsic target-binding affinity. Their relative contributions cannot be determined from the present data, and the design used here may accentuate structural effects: the library is comparatively small (N22, ∼10^13^ sequences), the target is one for which G-quadruplex binders are already known, and the fixed flanking sequences impose a stem. What the comparison does establish is the extent to which template structure impedes amplification and thereby distorts the composition of the surviving pool.

Analysis of aptamers discovered from conventional N40 random libraries reveals that a significant portion of the sequence space is typically occupied by stem regions rather than variable binding elements. The functional diversity that is actually accessible is therefore considerably lower than the theoretical complexity. In addition, an N40 region can encode ∼1.2 × 10^24^ sequences, whereas a selection typically starts from 10^14^ to 10^15^ molecules, so that only a very small fraction of the theoretical space is present in the starting pool. Our study addresses this inefficiency through a strategic pre-structured library design that fixes stem regions to 10 base pairs while dedicating the N22 variable region exclusively to target recognition (Supplementary Fig. S26). Previous studies comparing variable region lengths have validated the effectiveness of N20-sized sequences for aptamer discovery [33, 34].

This approach achieves structural complexity equivalent to conventional discoveries (10bp stem + 22bp variable + 10bp stem ≈ N42 equivalent) while maintaining manageable theoretical diversity of 4^22^ (≈1.76 × 10^13^) unique sequences. By aligning this complexity with the experimental input capacity (∼6–9 × 10^14^ molecules), each unique sequence was present at a comparable copy number (c₀ ≥ 50) in round 1. This was intended to control the starting conditions of the comparison: because the two selections differed only in the amplification enzyme, a uniform initial representation allows the losses observed over successive rounds to be attributed to amplification rather than to differences in the starting pool. The pre-structured hairpin backbone mimics the stem-loop architectures commonly observed in functional aptamers while ensuring that all sequence variability contributes directly to target binding rather than structural formation [35]. SPR analysis confirmed no nonspecific binding from the fixed stem sequences, validating that this design enhances evolutionary stability without compromising binding specificity (Supplementary Fig. S19B).

Within this broader framework of structure-induced amplification bias, G4 aptamers provide a useful representative case. Accordingly, our findings should not be interpreted as indicating that G4 structures represent the major structural class affected during SELEX. Rather, the thrombin model provides a well-characterized system in which the effect of amplification chemistry on the evolution of a specific stable structural motif can be directly examined.

In the present selections, SD-SELEX consistently recovered diverse G4 aptamers with affinities comparable to, or in some cases slightly better than, that of TBA-15. G4-containing sequences were progressively depleted during Taq-SELEX but maintained and enriched during SD-SELEX under otherwise identical selection conditions; because the two selections differed only in the amplification enzyme, this loss reflects structure-dependent amplification bias rather than differences in binding selection. This behavior was consistent with the broader trend observed for non-G4 structured sequences, particularly those containing extensive internal base pairing. Thus, the G4 results serve as a case study supporting the broader conclusion that amplification chemistry can reshape the structural composition of aptamer libraries during iterative SELEX.

Mechanistically, Taq polymerase exhibits limited strand-displacement capacity and tends to induce stalling or template switching (“jumping polymerase”), distorting sequence representation. In contrast, SD polymerase advances through structured regions by strand displacement without exonucleolytic cleavage of downstream strands, thereby increasing the likelihood of traversing structural barriers such as G4s, hairpins, or multi-loop motifs. As a result, cumulative structure-dependent bias across rounds is alleviated, enhancing the survival and recovery of structurally complex candidates.

We also examined whether this bias could be attributed to K⁺-dependent stabilization of G4 structures. KCl-gradient PCR and qPCR analyses showed that KCl concentration modestly affected the amplification efficiency of several G4-forming templates in a sequence-dependent manner, but these effects varied among templates and did not fully account for the observed amplification patterns: some templates remained inefficiently amplified regardless of KCl concentration, and comparable bias was observed for sequences predicted to form hairpins, stem–loops and other complex secondary structures. K⁺-dependent stabilization therefore contributes to amplification bias for certain templates but is not its sole determinant, and should be regarded as complementary to the properties of the polymerase itself.

In assessing the benefit of SD-SELEX, two comparisons should be distinguished. Relative to the reference aptamer TBA-15, the SD-SELEX candidates were comparable, differing by at most two- to three-fold, a difference that did not reach statistical significance. Relative to the candidates recovered by Taq-SELEX under identical selection conditions, the difference was larger, up to approximately one order of magnitude. Even so, the principal advantage of SD-SELEX is not affinity itself but the reduction of structure-induced amplification bias, which preserves a broader and structurally more diverse candidate pool and thereby increases the number of functional candidates available for screening.

Importantly, this pattern was not restricted to the thrombin selection. In parallel SELEX against VEGF-165, SD polymerase similarly preserved structurally complex sequences and a known functional motif that was depleted during Taq-based selection. The recurrence of this pattern with an independently selected target supports the interpretation that the observed structural bias is primarily associated with the amplification step rather than with target-specific properties.

Although SD-SELEX reduced the loss of structurally complex sequences in the present study, further improvements in polymerase fidelity and strand-displacement activity may help minimize amplification-driven structural bias and improve the robustness of the selection process.

Beyond iterative selection, the structure-dependent amplification bias characterized here may also be relevant to other PCR-based applications, such as library preparation for high-throughput sequencing. Although the bias is not compounded over successive rounds in these contexts, its magnitude at low cycle numbers is not negligible: a difference in structural preservation between SD and Taq polymerase was already apparent within the first two selection rounds (Fig. 2B and C), corresponding to ∼15–16 cumulative PCR cycles (Supplementary Table S4), a range comparable to that commonly used in sequencing library construction. For this reason, all sequencing libraries in the present study were prepared with SD polymerase regardless of the polymerase used during selection, so that the readout itself would not carry an additional structure-dependent distortion. More broadly, strand-displacing polymerases may help limit the underrepresentation of structured templates in PCR-based workflows, although the magnitude of this effect will depend on the template composition and amplification conditions of each specific application.

In conclusion, our results demonstrate that amplification chemistry is an important determinant of the structural composition of evolving SELEX libraries. SD polymerase reduced the preferential loss of structurally complex sequences observed during conventional Taq-based amplification, thereby preserving a broader range of structural diversity throughout iterative selection. By maintaining sequence classes that may otherwise be depleted during amplification, SD-SELEX may expand the accessible candidate space for downstream aptamer screening and development.

## Supplementary Material

gkag1186_Supplemental_File

## Data Availability

The raw sequence data reported in this paper have been deposited in the Korea Sequence Read Archive (KRA) in Korea Bioinformation Center, Korea Research Institute of Bioscience and Biotechnology (KAP242522) that are publicly accessible at https://kbds.re.kr/KRA.
