# Supplementary material for: Strand-displacement amplification reduces structure-induced PCR bias and preserves structurally diverse aptamers during SELEX
Source: Nucleic Acids Res. 2026 Nov 11;54(21):gkag1186. doi: 10.1093/nar/gkag1186 (PMC13752511; doi:10.1093/nar/gkag1186)
Supplement: Supplementary file 1 [file gkag1186_supplemental_file.pdf]

Supplementary information

Strand-displacement amplification reduces structure-induced PCR bias and preserves structurally diverse aptamers during SELEX

Hwayeon Jeong[1,†], Chang Beom Jeong[2,†], Jae Yun Moon[2], Daechan Park[2,*] and Cheulhee Jung[1,*]

[1]Department of Biotechnology, College of Life Sciences and Biotechnology, Korea University, 145, Anam-ro, Seongbuk-gu, Seoul 02841, Republic of Korea

[2]Department of Molecular Science and Technology, College of Advanced Bio-Convergence Engineering, Ajou University, Suwon 16499, Republic of Korea

*To whom correspondence should be addressed. Email: dpark@ajou.ac.kr or damo363@korea.ac.kr

†Hwayeon Jeong and Chang Beom Jeong contributed equally to this work.

Table of Contents

Supplementary Figure S1 4
Supplementary Figure S2 5
Supplementary Figure S3 6
Supplementary Figure S4 7
Supplementary Figure S5 8
Supplementary Figure S6 9
Supplementary Figure S7 10
Supplementary Figure S8 11
Supplementary Figure S9 12
Supplementary Figure S10 13
Supplementary Figure S11 14
Supplementary Figure S12 15
Supplementary Figure S13 16
Supplementary Figure S14 17
Supplementary Figure S15 18
Supplementary Figure S16 19
Supplementary Figure S17 20
Supplementary Figure S18 21
Supplementary Figure S19 22
Supplementary Figure S20 23
Supplementary Figure S21 24
Supplementary Figure S22 25
Supplementary Figure S23 26
Supplementary Figure S24 27
Supplementary Figure S25 28
Supplementary Figure S26 29
Supplementary Table S1 30
Supplementary Table S2 32
Supplementary Table S3 35
Supplementary Table S4 36

Supplementary Table S5 37
Supplementary Table S6..38

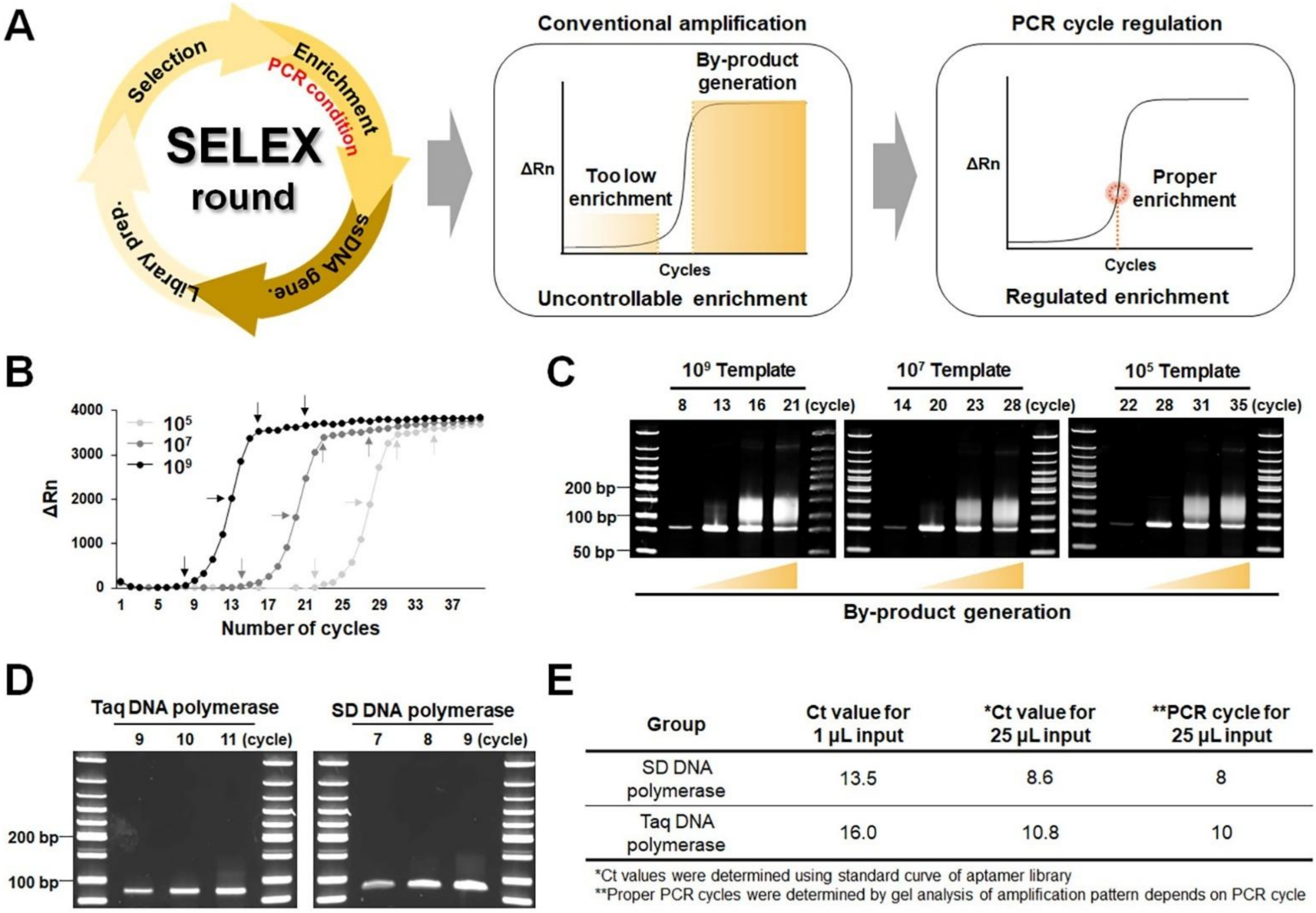

Group	Ct value for 1 μL input	*Ct value for 25 μL input	**PCR cycle for 25 μL input
SD DNA polymerase	13.5	8.6	8
Taq DNA polymerase	16.0	10.8	10

*Ct values were determined using standard curve of aptamer library
**Proper PCR cycles were determined by gel analysis of amplification pattern depends on PCR cycle

Supplementary Figure S1. Schematic illustration for PCR cycle regulation during the enrichment stage of the SELEX process. (**A**) During the amplification of the aptamer library in the SELEX process, excessive PCR cycles can cause the formation of by-products. However, too few cycles result in inefficient evolution of selected libraries, as there is no step for PCR cycle adjustment in the conventional process. (**B**) Ct values were obtained through real-time qPCR and (**C**) confirmed using native gel electrophoresis to determine the amount of by-products produced, varying the aptamer library and PCR cycles. Based on this, we tried to predict the appropriate PCR cycle based on the Ct values from real-time qPCR. In particular, to predict the PCR cycle for 25 μl of eluent using 1 μl of selection eluent, (**D**) we confirmed the suitable PCR cycle value for 25 μl of eluent based on the real-time qPCR Ct value for 1 μl, as shown by native gel electrophoresis. The actual PCR cycle values resulting from this analysis are presented in (**E**).

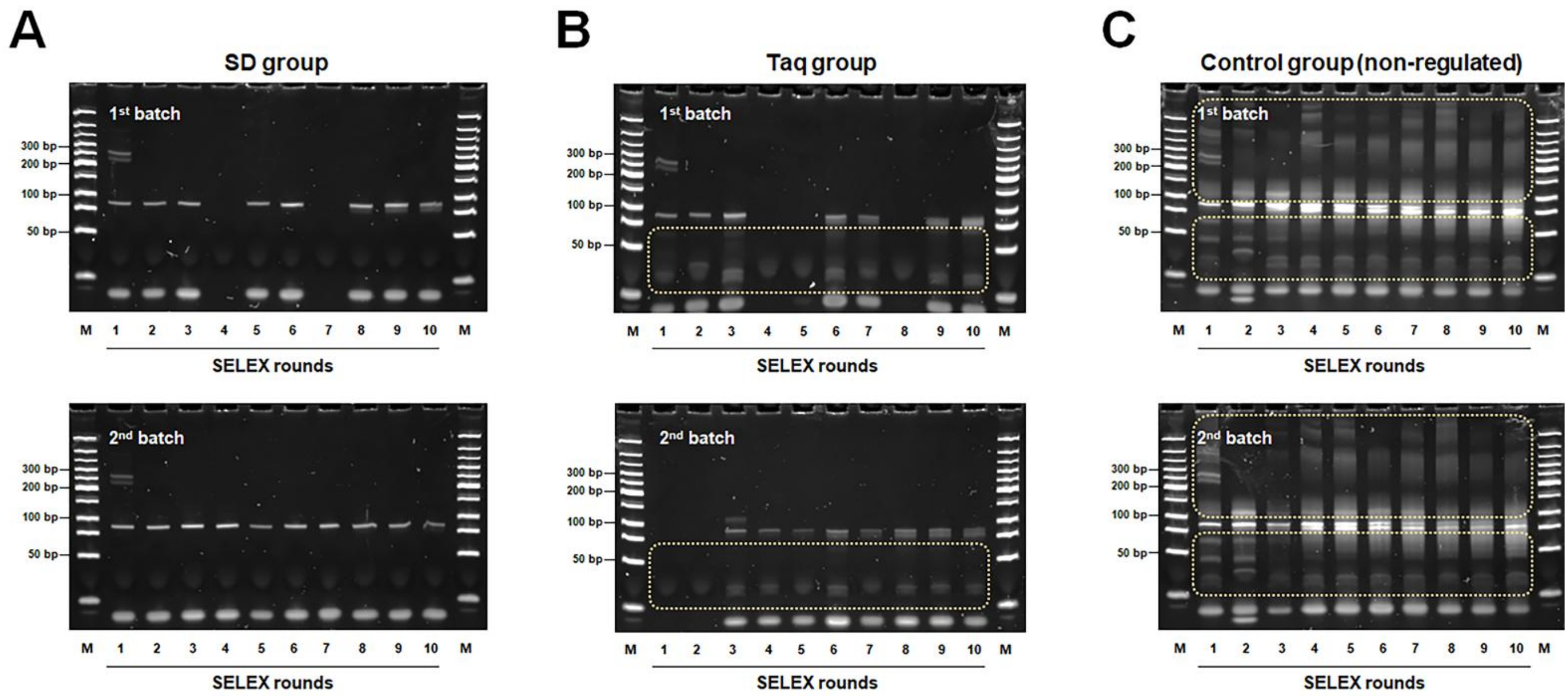

Supplementary Figure S2. Analysis of PCR products in SELEX rounds. The results of native gel analysis for PCR amplicons from each SELEX round are shown for the experimental groups of (**A**) SD polymerase (1st and 2nd batch), (**B**) Taq polymerase (1st and 2nd batch), and (**C**) the control (1st and 2nd batch). SD and Taq groups employed PCR cycle regulation throughout all rounds. In the control groups, Taq polymerase was used for SELEX without applying PCR cycle regulation. Some samples from the SD and Taq groups were lost, resulting in the absence of detectable bands.

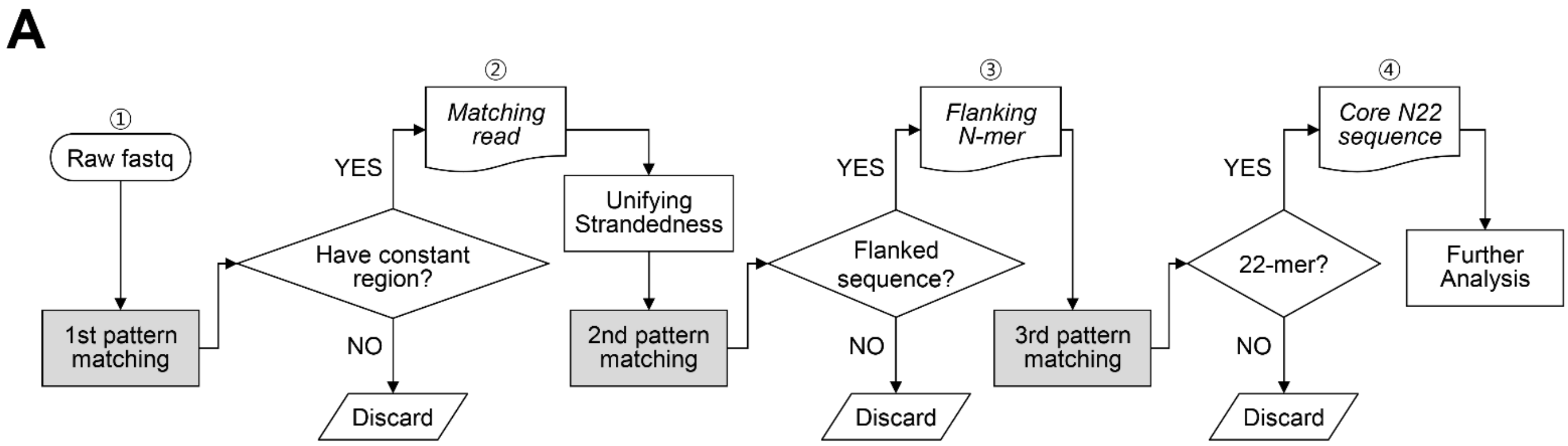

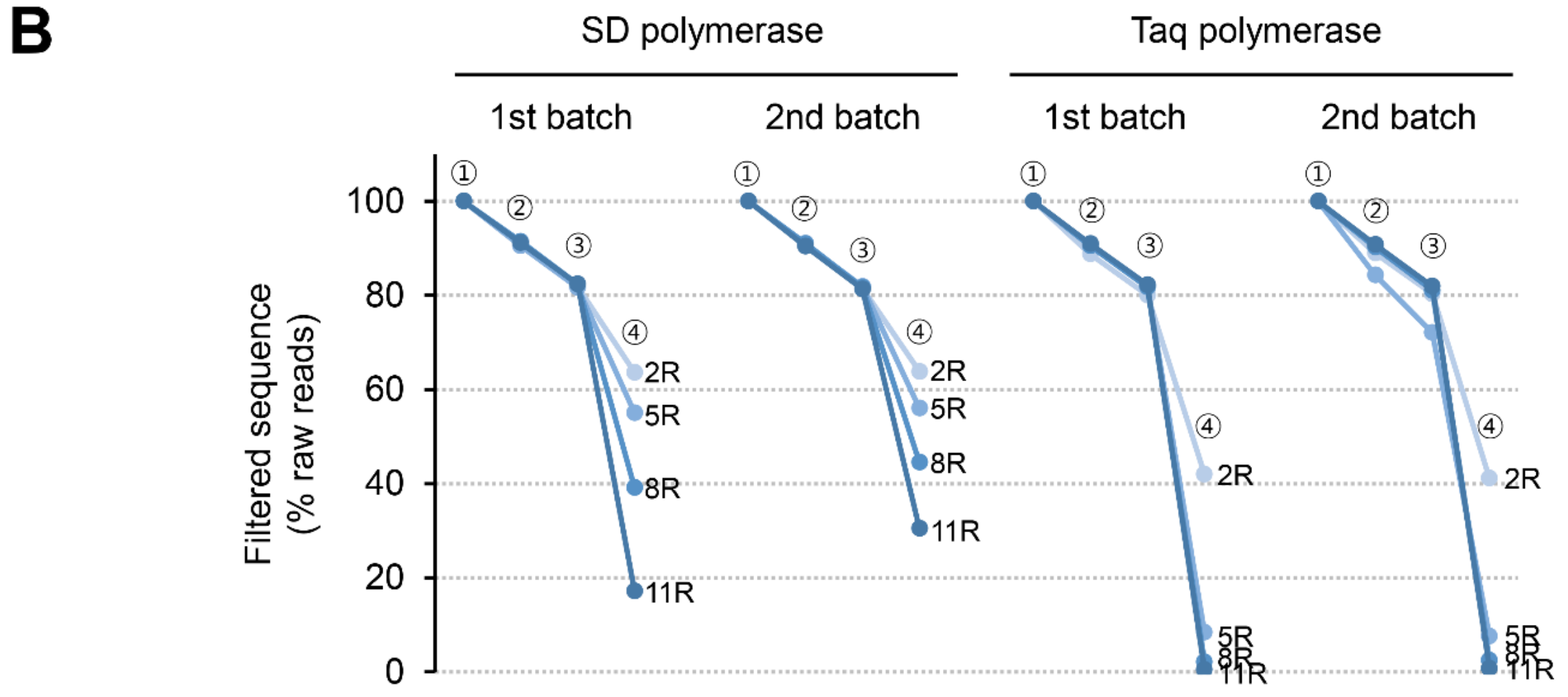

			① Raw reads	② Matching reads	③ Flanking N-mer	④ N22
SD	1st batch	2R	115,796,062	105,188,744	94,717,801	73,690,673
		5R	119,407,904	108,049,653	97,506,820	65,744,301
		8R	125,695,744	114,947,968	103,527,118	49,240,902
		11R	169,118,398	154,110,244	139,310,432	29,070,730
	2nd batch	2R	90,781,938	82,662,519	74,045,690	57,955,671
		5R	103,488,558	93,976,481	84,760,184	57,965,862
		8R	144,382,950	131,397,994	117,967,617	64,262,182
		11R	183,268,526	165,683,415	149,082,591	55,853,035
Taq	1st batch	2R	103,515,198	91,886,537	82,874,997	43,502,902
		5R	110,152,276	100,082,661	90,343,938	9,245,170
		8R	83,760,492	75,810,803	68,488,738	1,814,285
		11R	139,874,536	127,249,249	115,064,827	644,340
	2nd batch	2R	97,499,822	86,716,217	78,314,293	40,140,787
		5R	100,872,500	85,046,217	72,719,345	7,709,821
		8R	107,583,808	97,087,908	87,219,381	2,677,677
		11R	139,630,732	126,756,806	114,391,514	1,106,700

Supplementary Figure S3. Workflow for aptamer sequence processing. (**A**) Workflow for aptamer sequence processing. (**B**) Results for sequence filtering.

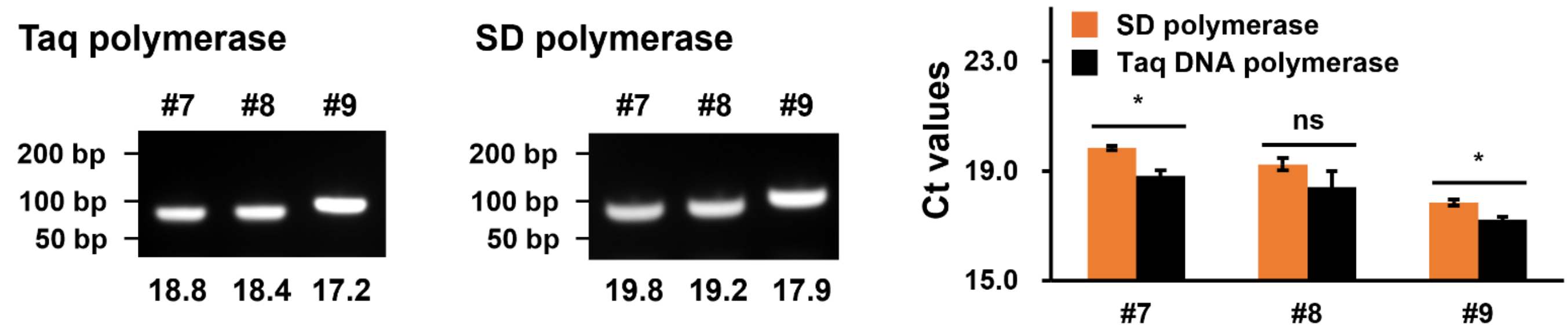

Supplementary Figure S4. Comparison of amplification efficiencies of SD and Taq DNA polymerases using various templates. The amplification efficiencies of SD and Taq DNA polymerases for templates #7, #8, and #9 were comparatively analyzed using 2% agarose gel electrophoresis and qPCR. Data are presented as the mean ± SD of three replicates (n = 3). Statistical significance was determined using a two-tailed paired Student's t-test. *P < 0.05; ns, not significant.

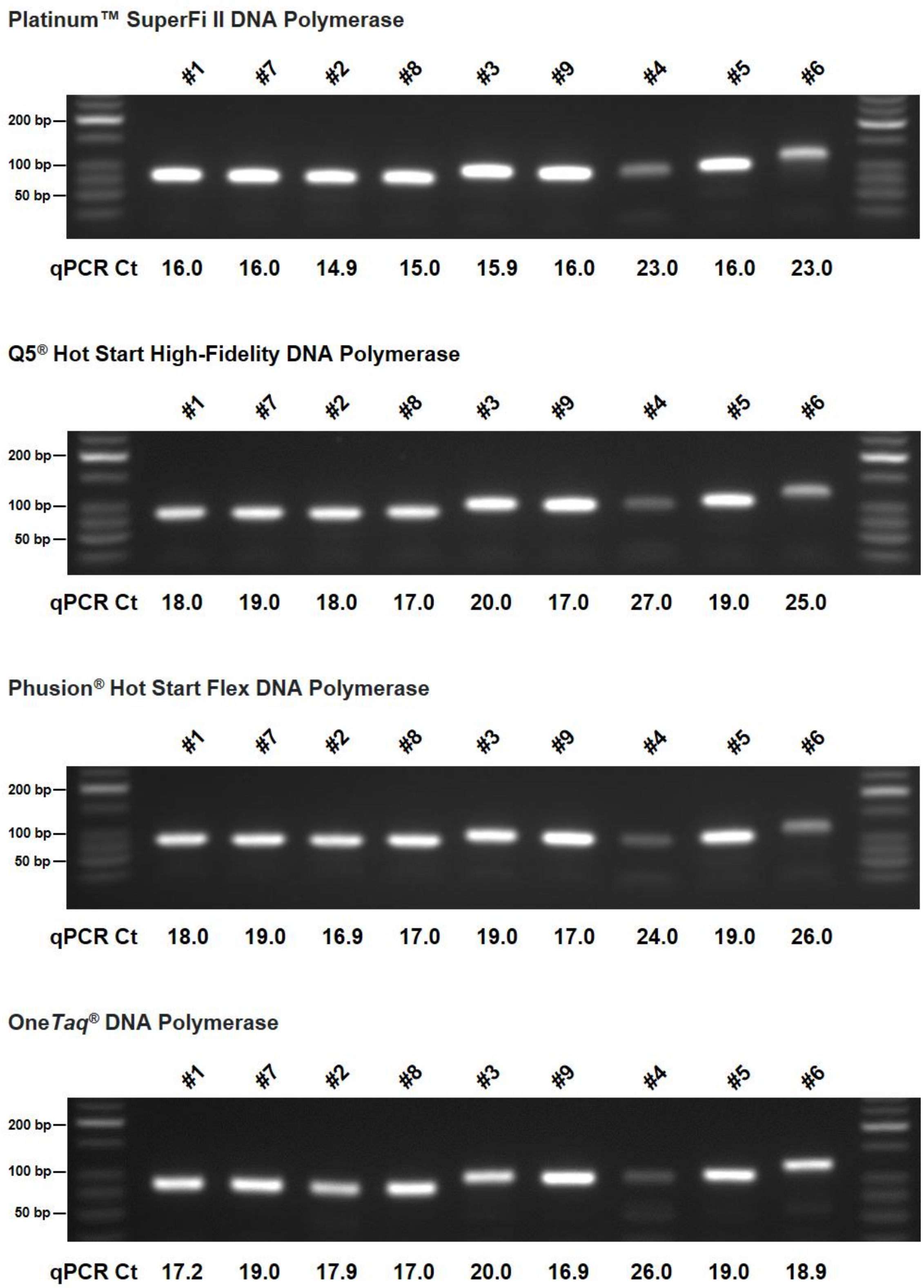

Supplementary Figure S5. Analysis of amplification efficiency of various DNA Polymerases. The amplification efficiency of Platinum™ SuperFi II DNA Polymerase, Q5® Hot Start High-Fidelity DNA Polymerase, Phusion Hot Start II High-Fidelity PCR Master Mix, and OneTaq® DNA Polymerase for templates of various structures was analyzed using Ct values and 2% agarose gel after real-time qPCR and PCR.

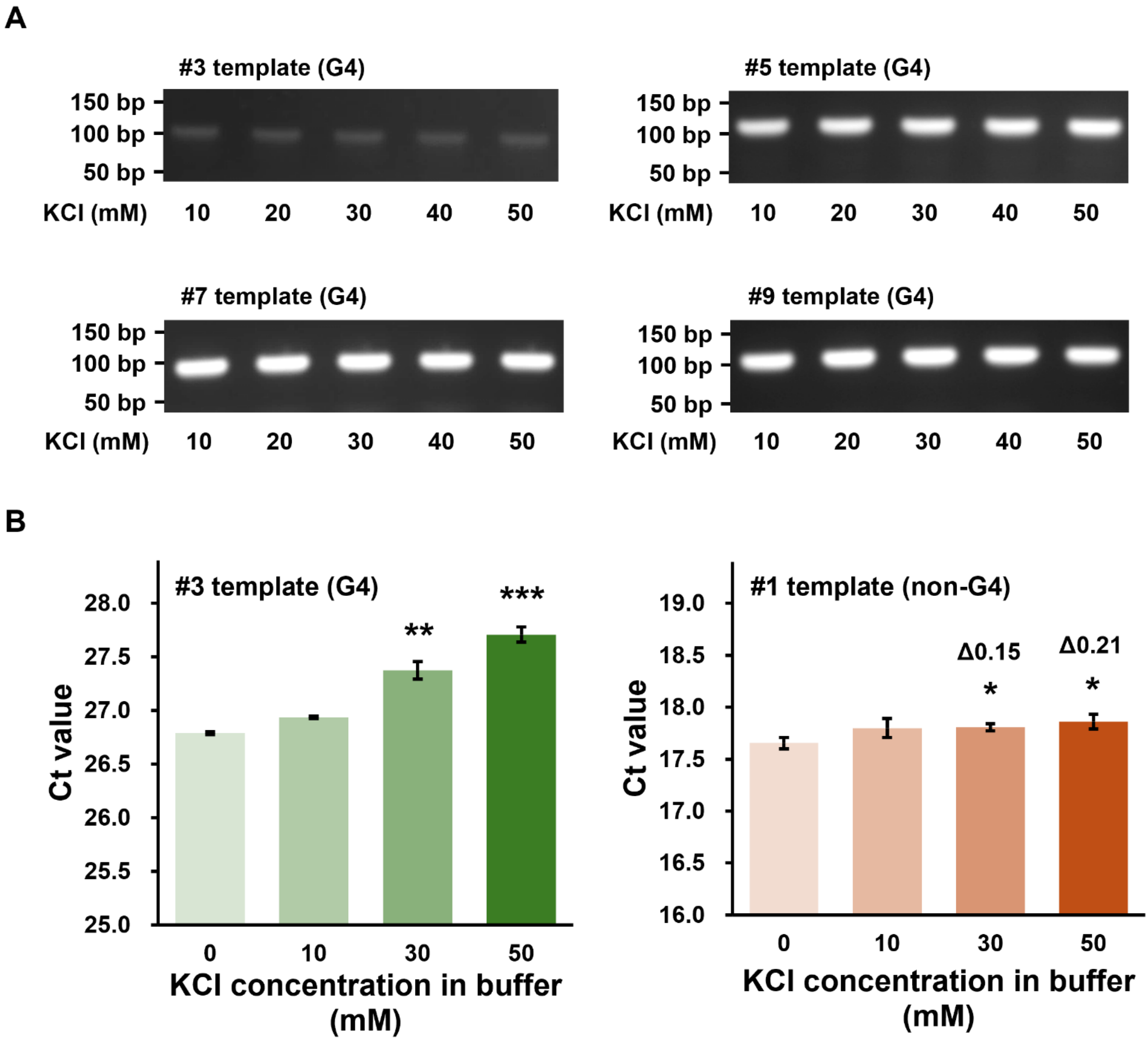

Supplementary Figure S6. Effect of KCl concentration on amplification by Taq DNA polymerase using various templates. (**A**) Templates #3, #5, #7, and #9, which form G-quadruplex (G4) structures, were amplified by Taq DNA polymerase in reaction buffers containing different concentrations of KCl, and the resulting gel-band intensities were quantified and compared. (**B**) The effects of KCl concentration on amplification of the G4-forming template #3 and the non-G4-forming template #1 by Taq DNA polymerase were compared using qPCR. Data are presented as the mean ± SD of three replicates and were compared with the 0 mM KCl condition (n = 3). Statistical significance was determined using a two-tailed paired Student's t-test. $^{*}P < 0.05$; $^{**}P < 0.01$; $^{***}P < 0.001$.

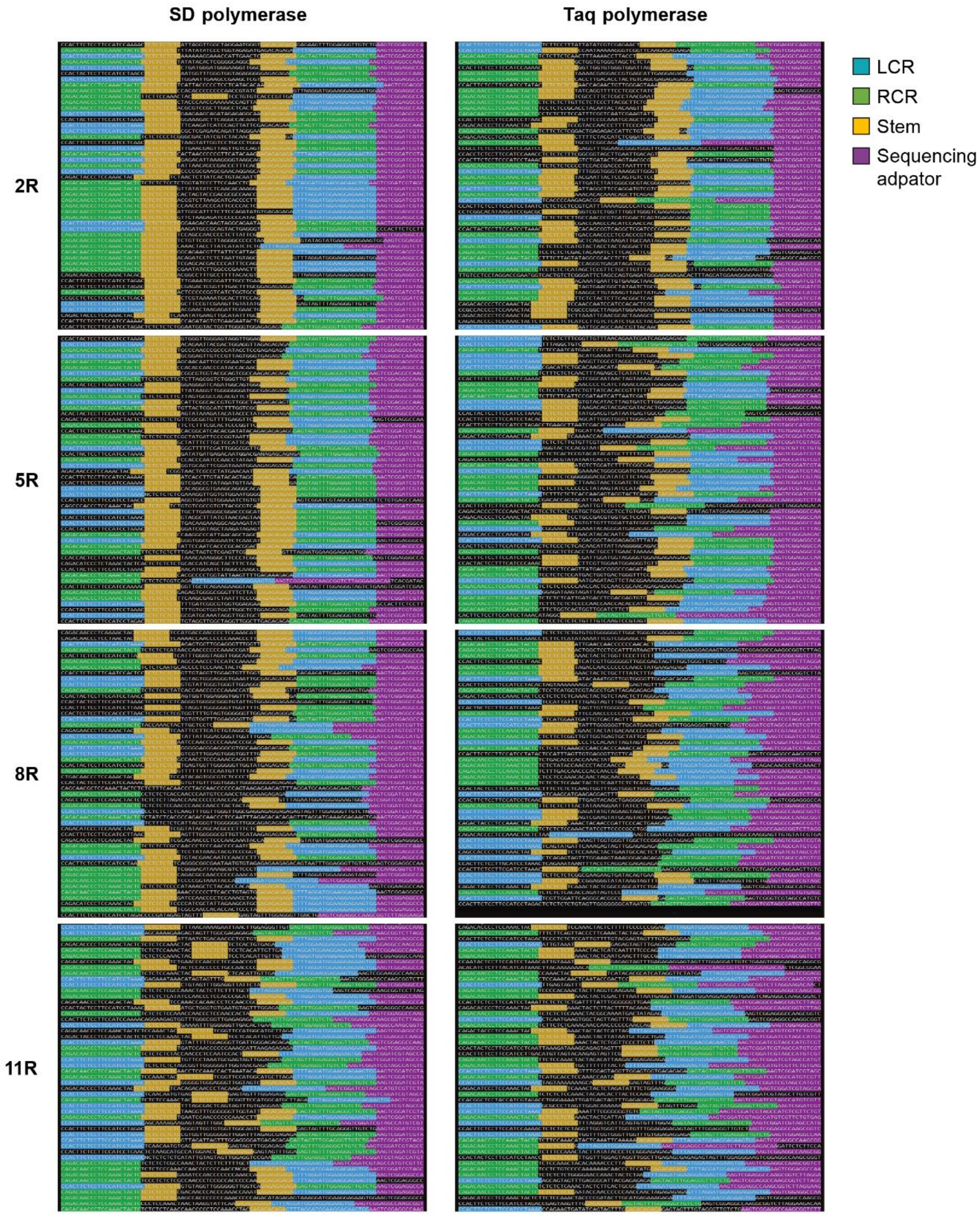

Supplementary Figure S7. Structure of the aptamer library in raw reads. Paired-end reads from both first and second batches were merged for visual inspection. Total 50 sequences were representatively shown as a first read extracted for every 4 M reads.

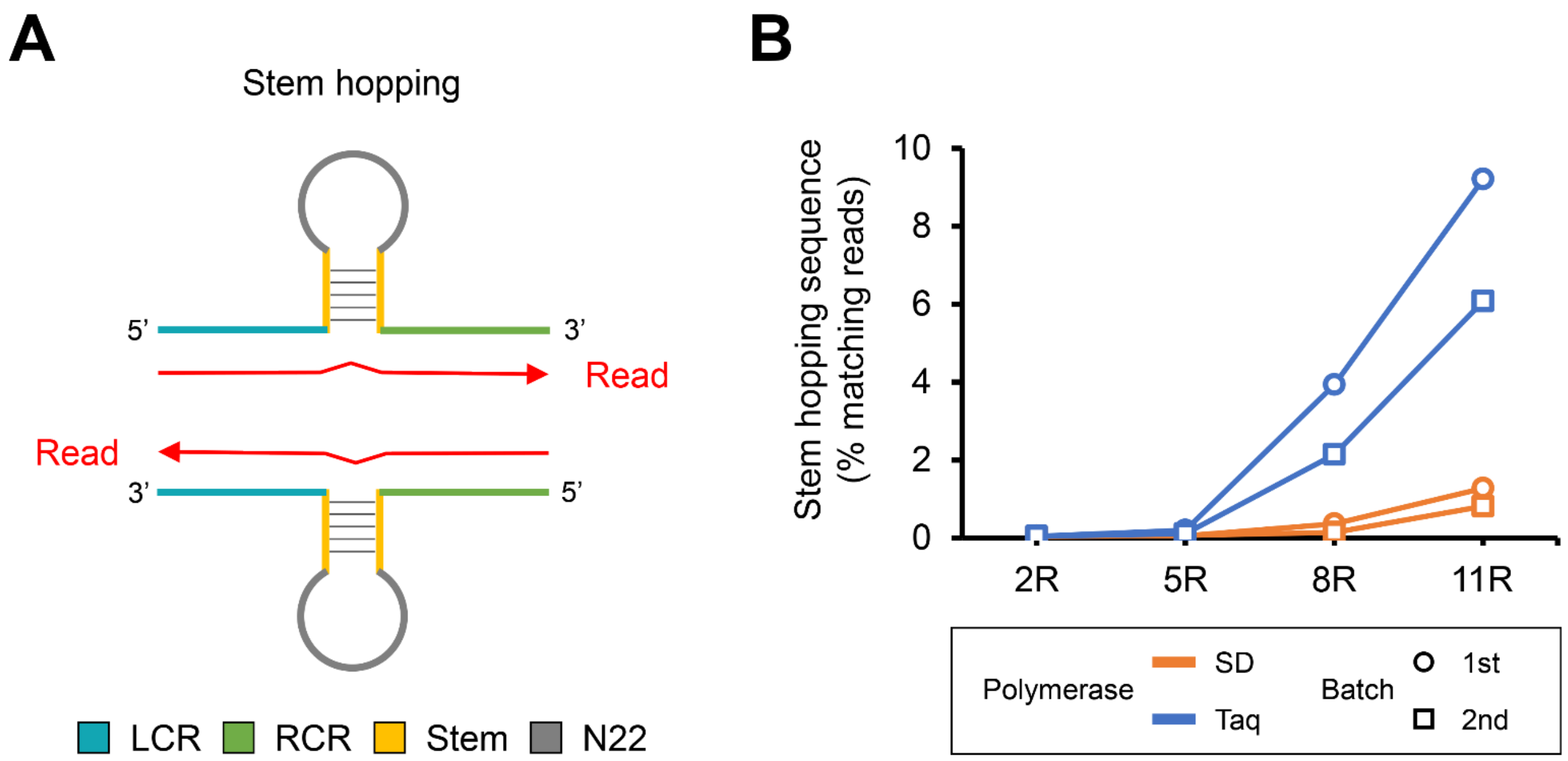

Supplementary Figure S8. Analysis of stem hopping phenomena. (**A**) Illustration describing stem hopping phenomena. (**B**) Proportion of the stem hopping sequences between SELEX using SD polymerase and Taq polymerase.

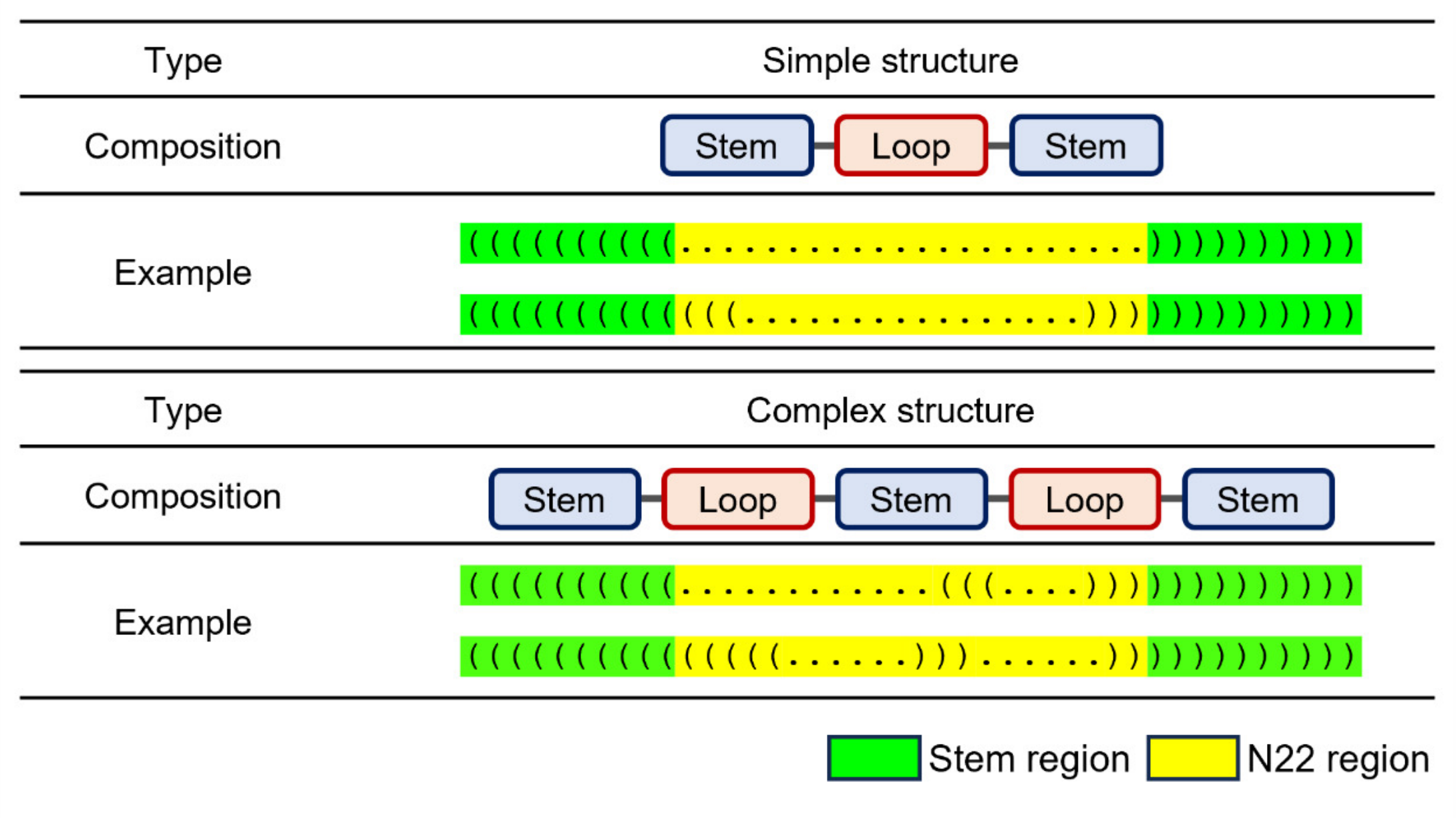

Supplementary Figure S9. Classification of predicted secondary structures into Simple and Complex groups. Representative structures in dot-bracket notation. Green shading marks the 10 bp stem region of the library and yellow shading marks the 22 bp random (N22) region. Simple structures form a single continuous stem-loop that encloses the random region; Complex structures contain additional base-paired motifs within the random region.

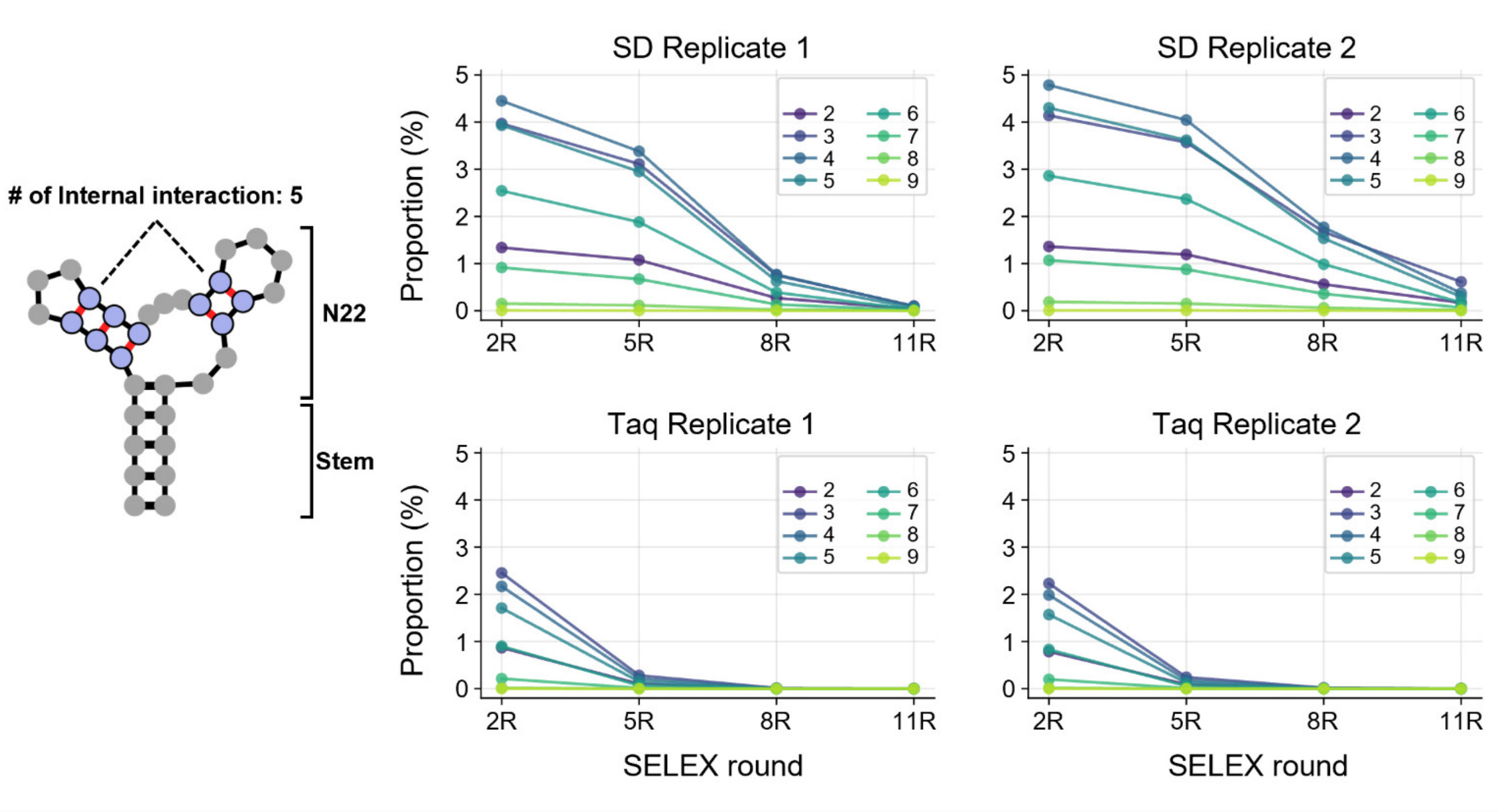

Supplementary Figure S10. Complex structures resolved by the number of internal base-paired interactions. The Complex group was separated by the number of base pairs (2 to 9) formed within the N22 random region and tracked across SELEX rounds for SD (top) and Taq (bottom) replicates. The schematic (left) shows a sequence with five internal interactions within the N22 region above the fixed stem.

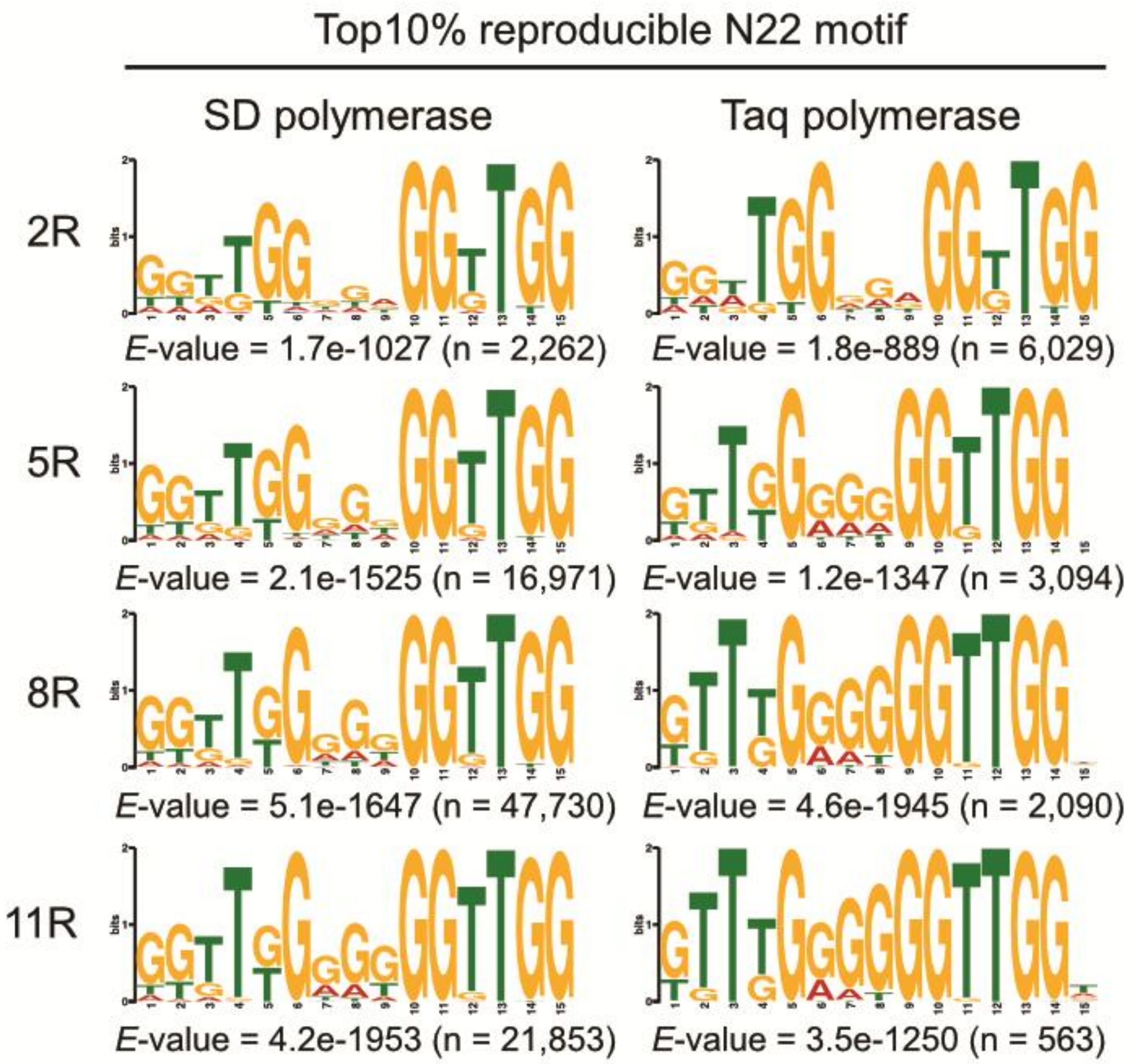

Supplementary Figure S11. Sequence motif analysis for the top-ranked reproducible N22 sequences. Consensus motif in N22 sequences were analyzed using MEME Suite (ver.5.5.4). E-value indicates statistical significance of the motif.

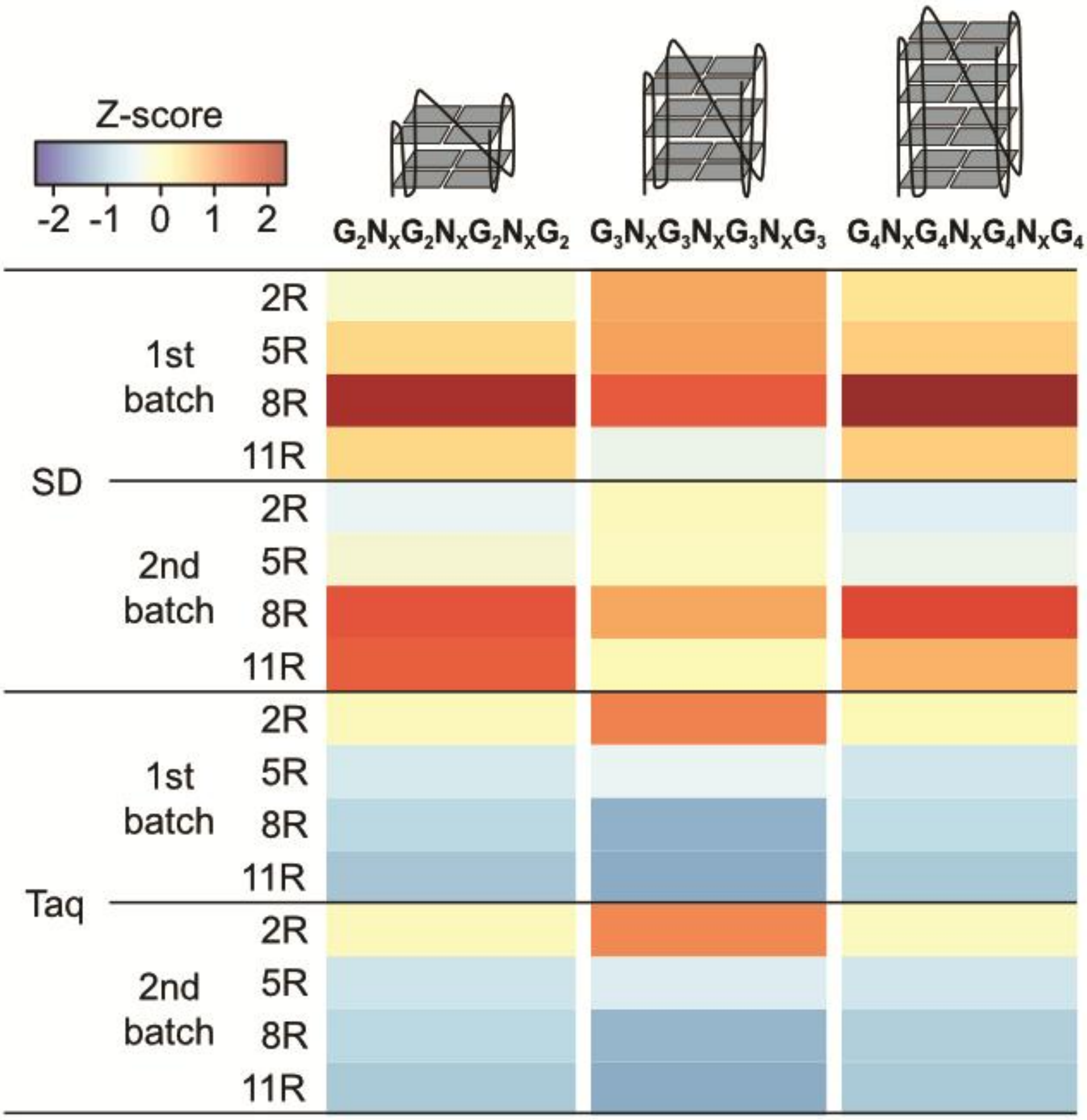

Supplementary Figure S12. Heat map showing the proportion of N22 sequences containing G-quadruplex motifs. Potential G-quadruplex motifs were defined based on the number of guanine tetrads as follows: G-quadruplex with 2-tetrad ($G_2N_xG_2N_xG_2N_xG_2$, x≥1), 3-tetrad ($G_3N_xG_3N_xG_3N_xG_3$, x≥1), and 4-tetrad ($G_4N_xG_4N_xG_4N_xG_4$, x≥1). The number of N22 sequences containing potential G-quadruplex motifs was counted from the total N22 sequence. Then, the number of sequences was divided by the total number of raw reads to calculate the proportion, and the proportions were normalized as Z-Score within G-quadruplex motif type.

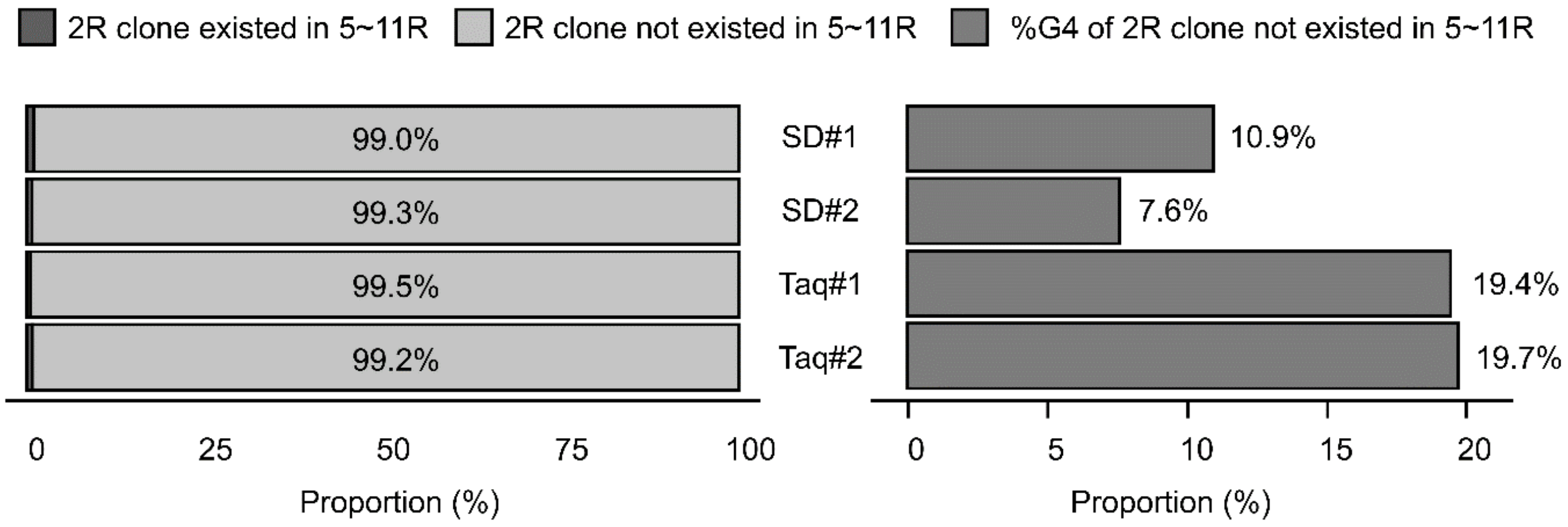

N22 sequence				G4-containing N22 sequence (%)			
2R	5R	8R	11R	SD#1	SD#2	Taq#1	Taq#2
+				10.9	7.6	19.4	19.7
+	+			54.0	35.1	72.2	71.9
+	+	+		88.4	75.0	83.4	83.0
+	+	+	+	65.3	48.6	26.7	67.5

Supplementary Figure S13. Proportion of N22 sequences containing potential 2 tetrad G-quadruplex (G4) by round progression.

A

15bp TBA sequence existed in			SELEX round 2R	5R	8R	11R
Total N22	SD	1st batch	23,023 (0.03%)	116,901 (0.18%)	214,935 (0.44%)	58,119 (0.20%)
		2nd batch	11,011 (0.02%)	37,639 (0.06%)	210,422 (0.33%)	185,203 (0.33%)
	Taq	1st batch	12,042 (0.03%)	6,241 (0.07%)	377 (0.02%)	35 (0.01%)
		2nd batch	9,731 (0.02%)	2,934 (0.04%)	456 (0.02%)	113 (0.01%)
Top10% weighted reproducible N22	SD	1st batch	4,133 (22.62%)	26,186 (7.72%)	78,566 (1.23%)	19,011 (0.23%)
		2nd batch	2,153 (14.35%)	10,708 (6.41%)	82,813 (1.21%)	51,652 (0.52%)
	Taq	1st batch	306 (0.64%)	14 (0.02%)	Out of top10%	Out of top10%
		2nd batch	244 (0.79%)	11 (0.04%)	Out of top10%	Out of top10%

B

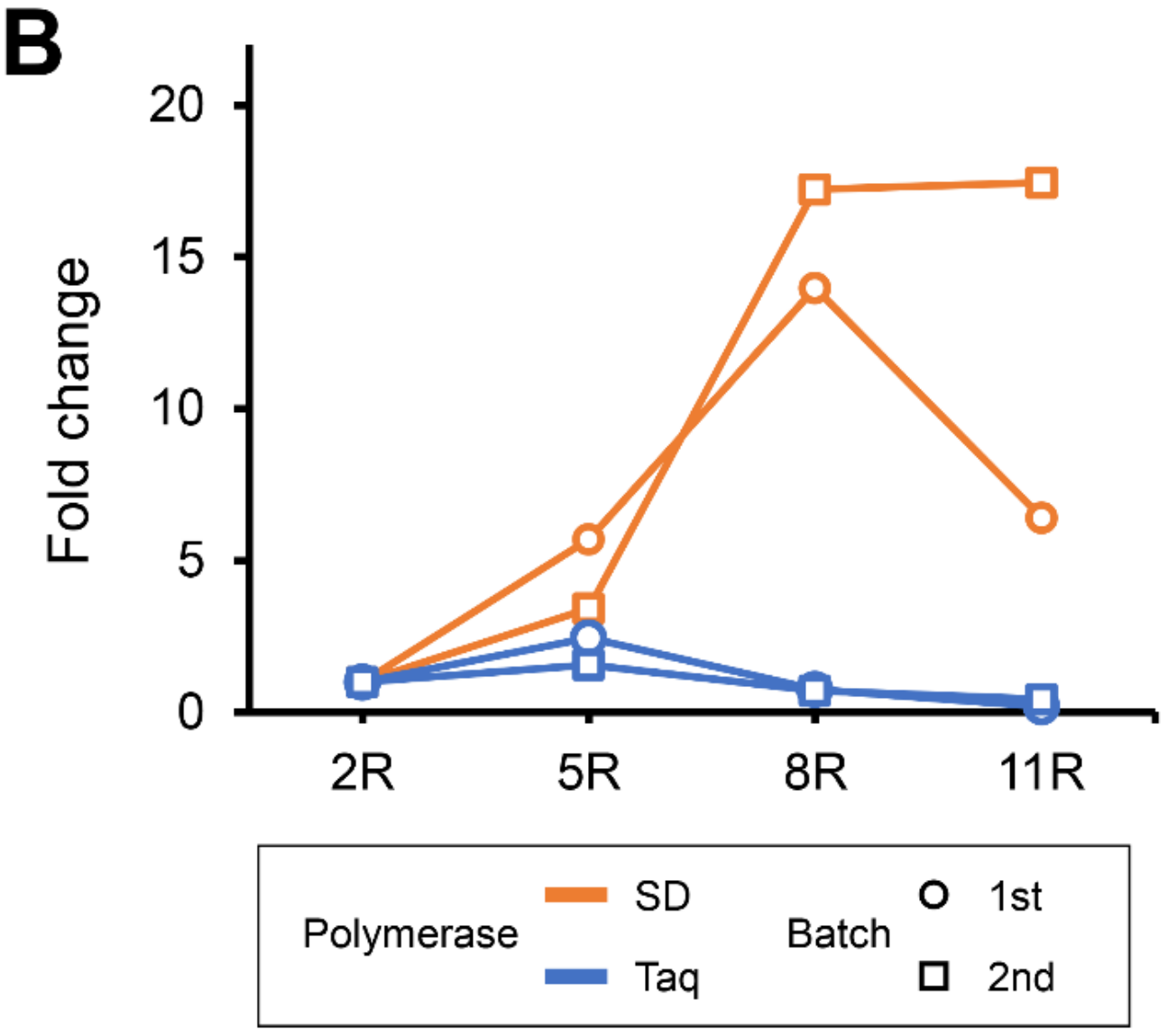

C

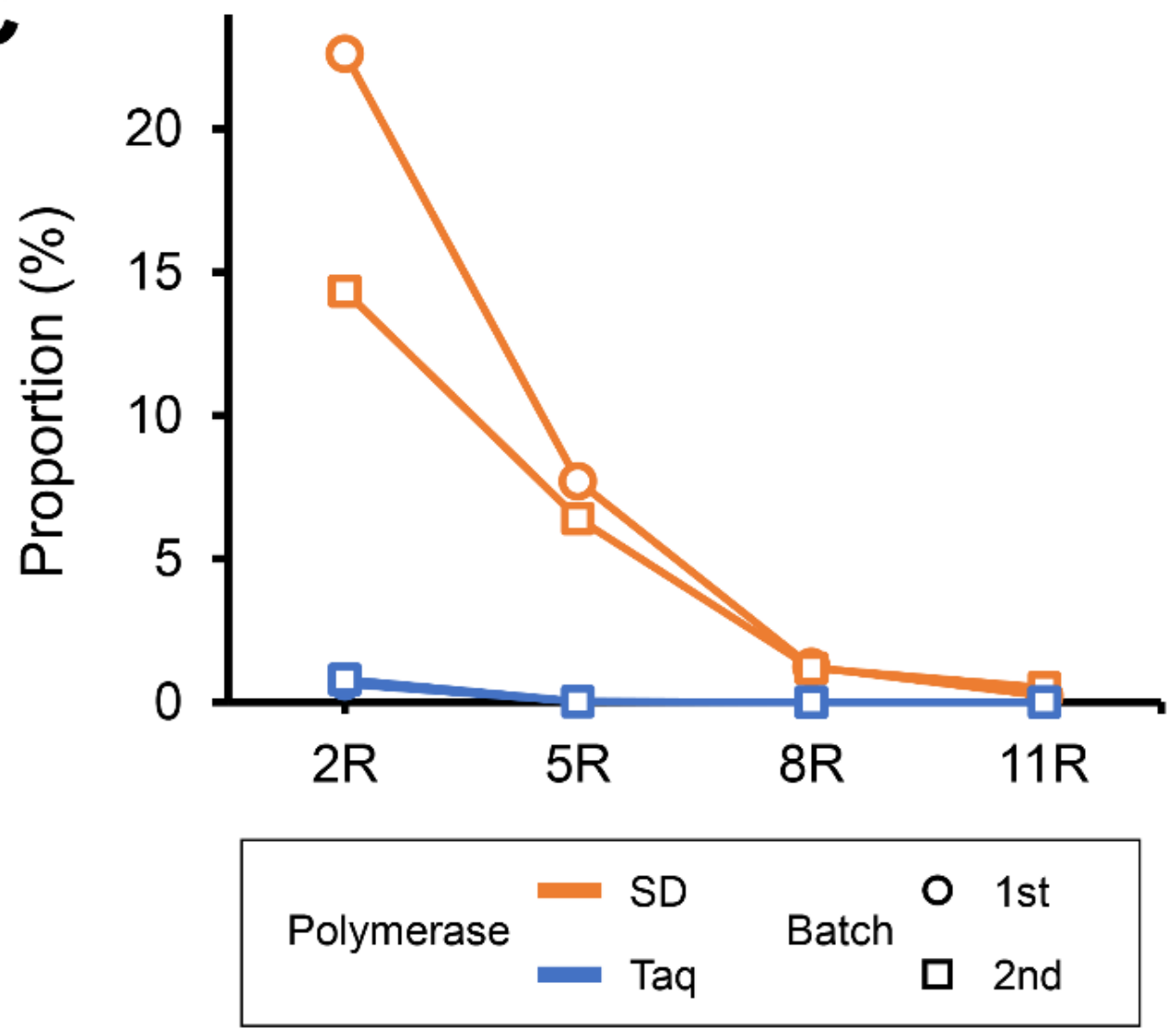

Supplementary Figure S14. Quantitative analysis of N22 sequence containing 15 bp TBA sequence. (**A**) Proportion of the sequences containing 15 bp TBA in total N22 and frequency top 10% reproducible N22. (**B**) Fold increase in the number of 15 bp TBA in total N22. (**C**) Decrease in the proportion of 15 bp TBA in the top ranked N22 sequences.

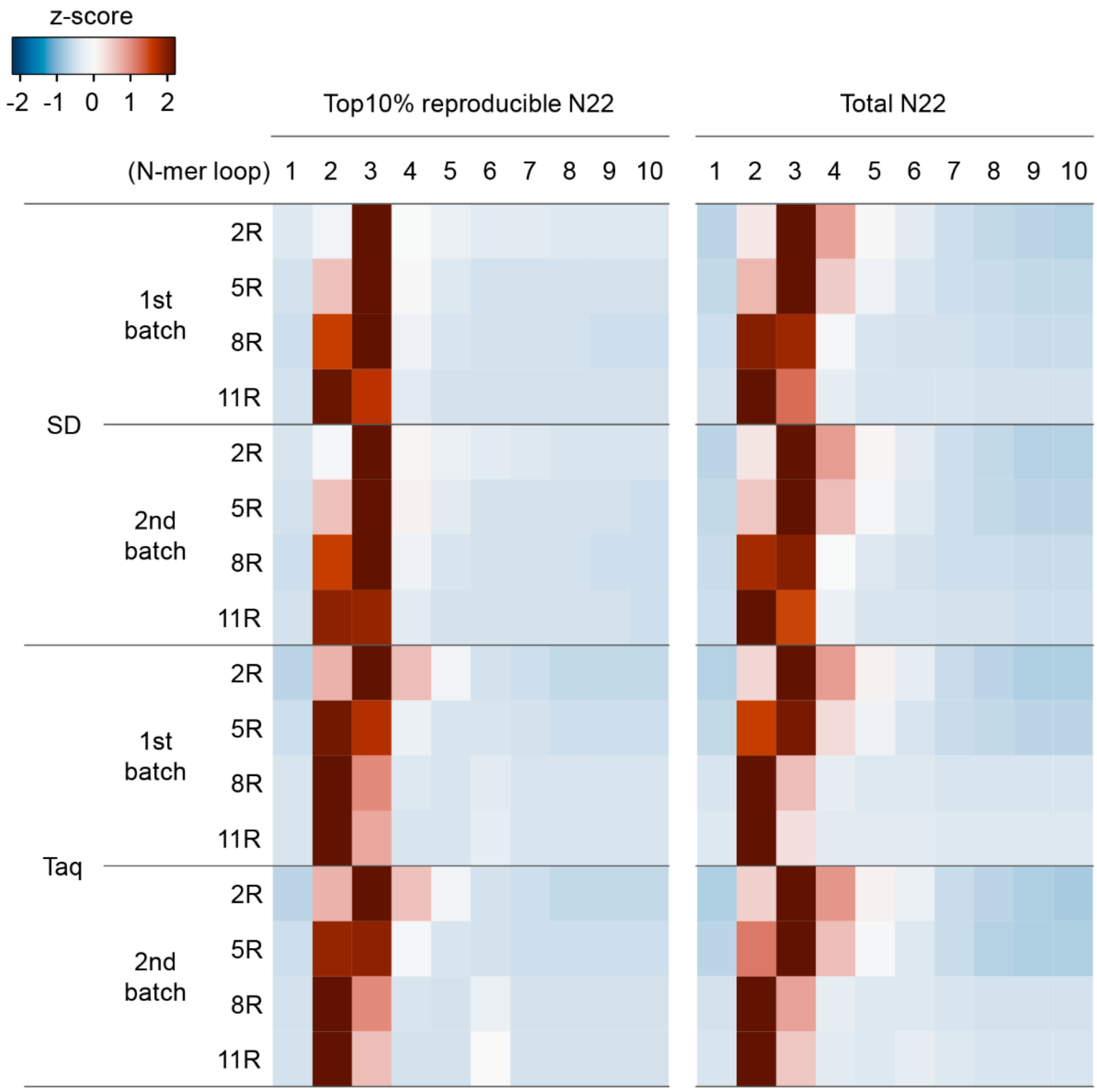

Supplementary Figure S15. Frequency of loop sequences within GGTTGG-(loop)-GGTTGG motif containing N22 sequence by length.

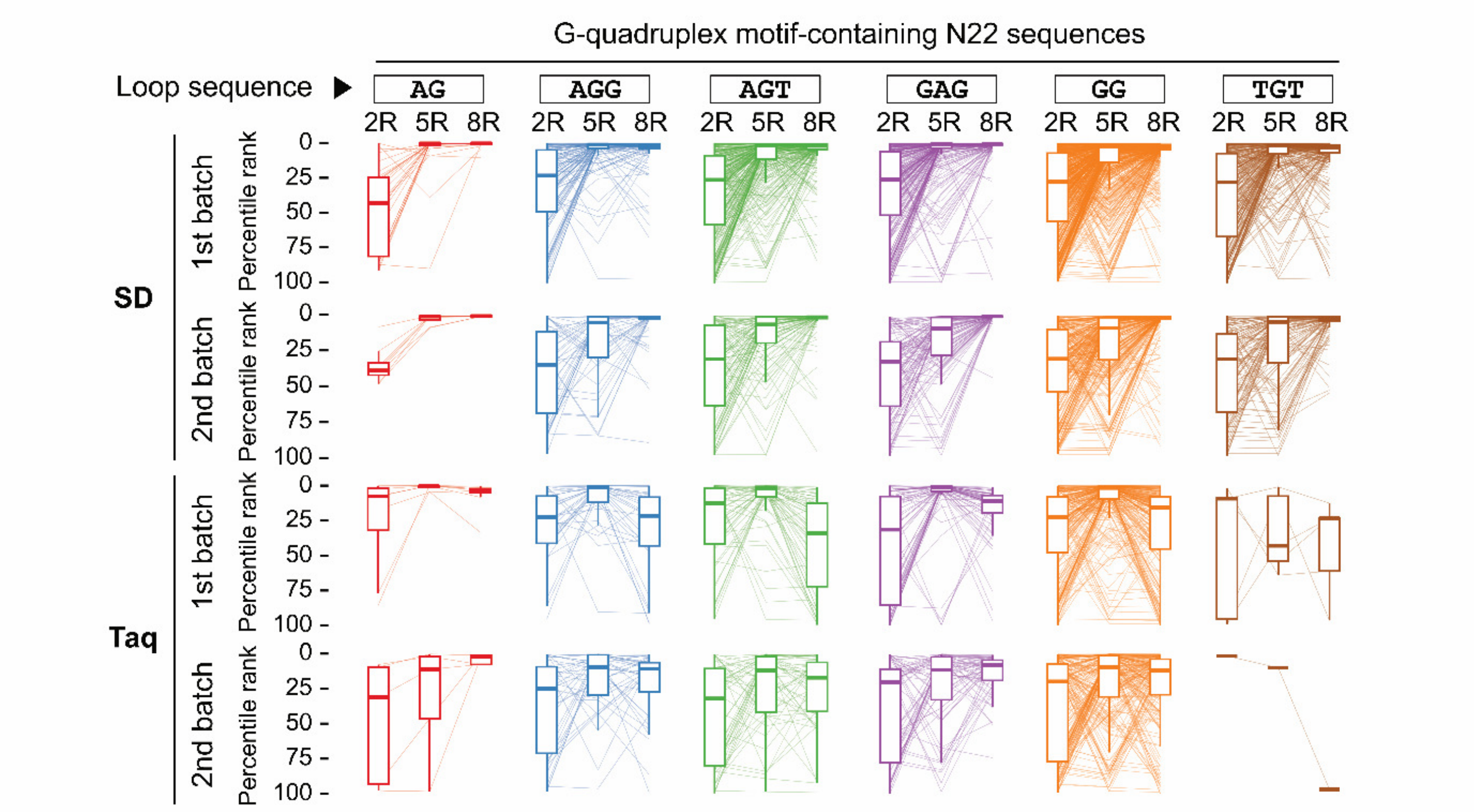

Supplementary Figure S16. Evolution of G-quadruplex motif-containing N22 sequences was tracked based on rank percentiles by frequency. Percentile rank of the sequence for each round was calculated as following equation: [(Frequency rank of the motif-containing N22 sequence in each round)/(Total number of unique N22 sequences of the corresponding round)] × 100. Each line of the plots represents a respective N22 sequence containing the indicated G-quadruplex motif. The boxplot shows the rank distribution of G-quadruplex motif-containing N22 sequences for each round.

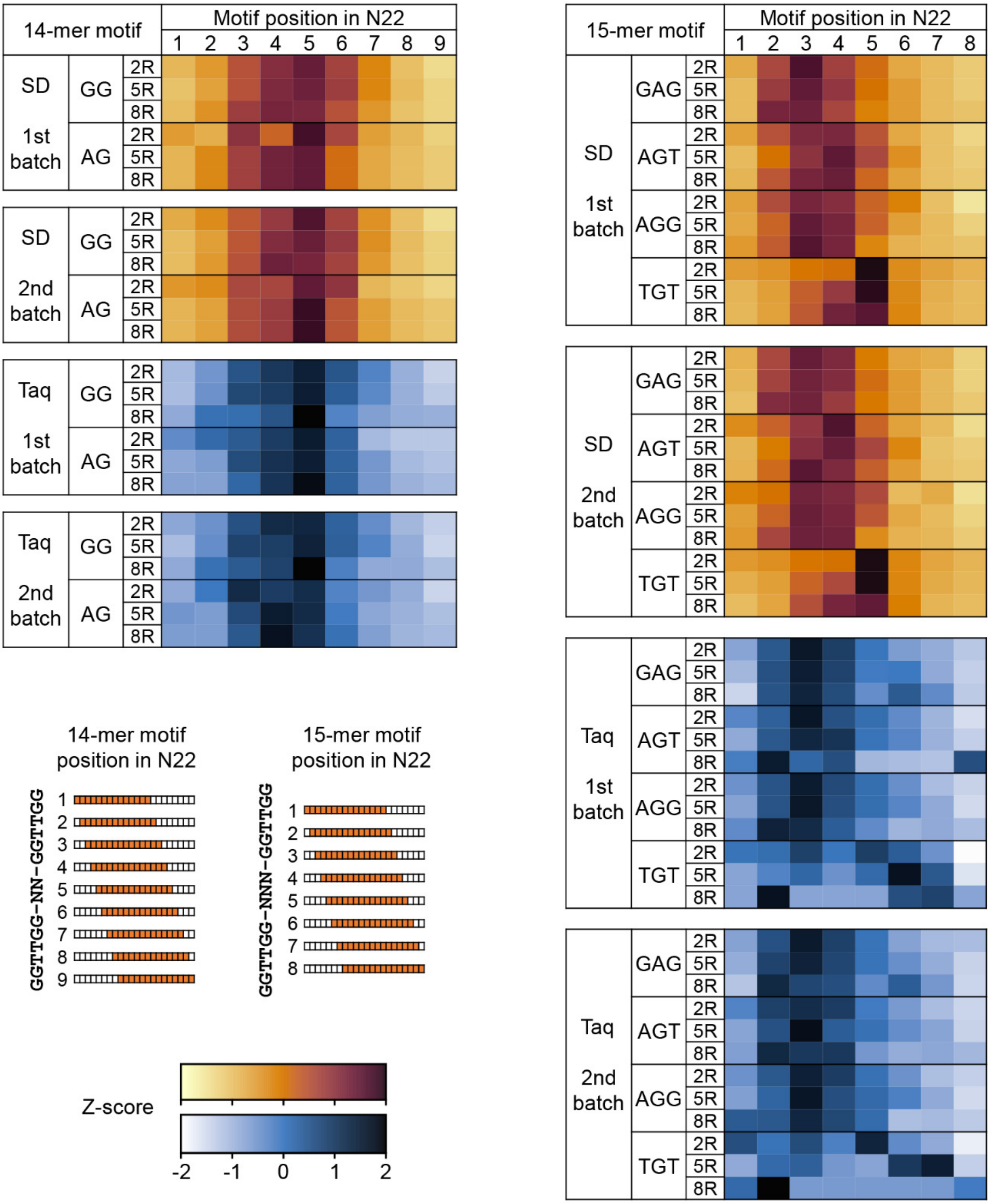

Supplementary Figure S17. Distribution of the G-quadruplex motif within the N22 sequence. Frequency top 10% N22 sequences for each round were analyzed. The number of possible positions for each motif within the N22 sequence is 9 for the 14-mer G-quadruplex and 8 for the 15-mer G-quadruplex. The proportion of motif in each position was calculated as follows and normalized as Z-Score: Proportion = (number of motifs present at each position) / (total number of N22s including the corresponding motif).

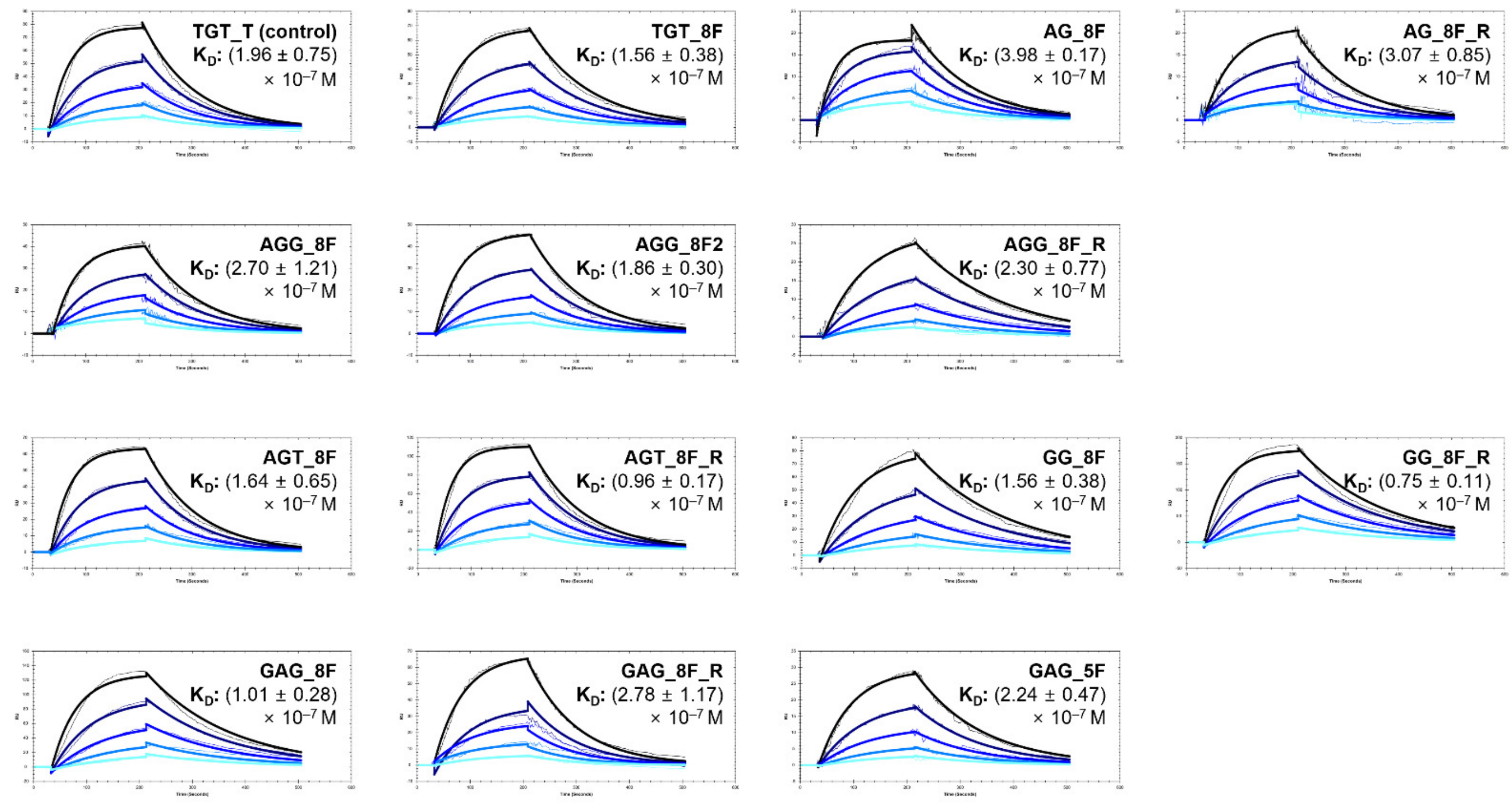

Supplementary Figure S18. Specific binding analysis of selected aptamer libraries. Binding analysis of the aptamers candidates from SD groups to thrombin by an SPR system at 25°C. The sequence name (NNN_number) comprises the sequence selected through the SELEX round corresponding to the loop in the motif (GGTTGG-loop-GGTTGG). Additionally, the F after the round represents the entire sequence, including the stem sequence. T denotes a truncated sequence containing only the motif. R indicates a sequence commonly identified in both the first and second batches. All sequences are selected based on the highest frequency ranking within each SELEX round. Each aptamer's dissociation constant (K_D) to targeted thrombin was determined. Data are presented as the mean ± SD of three independent measurements (n = 3). K_D values were calculated individually from k_{off}/k_{on} for each measurement.

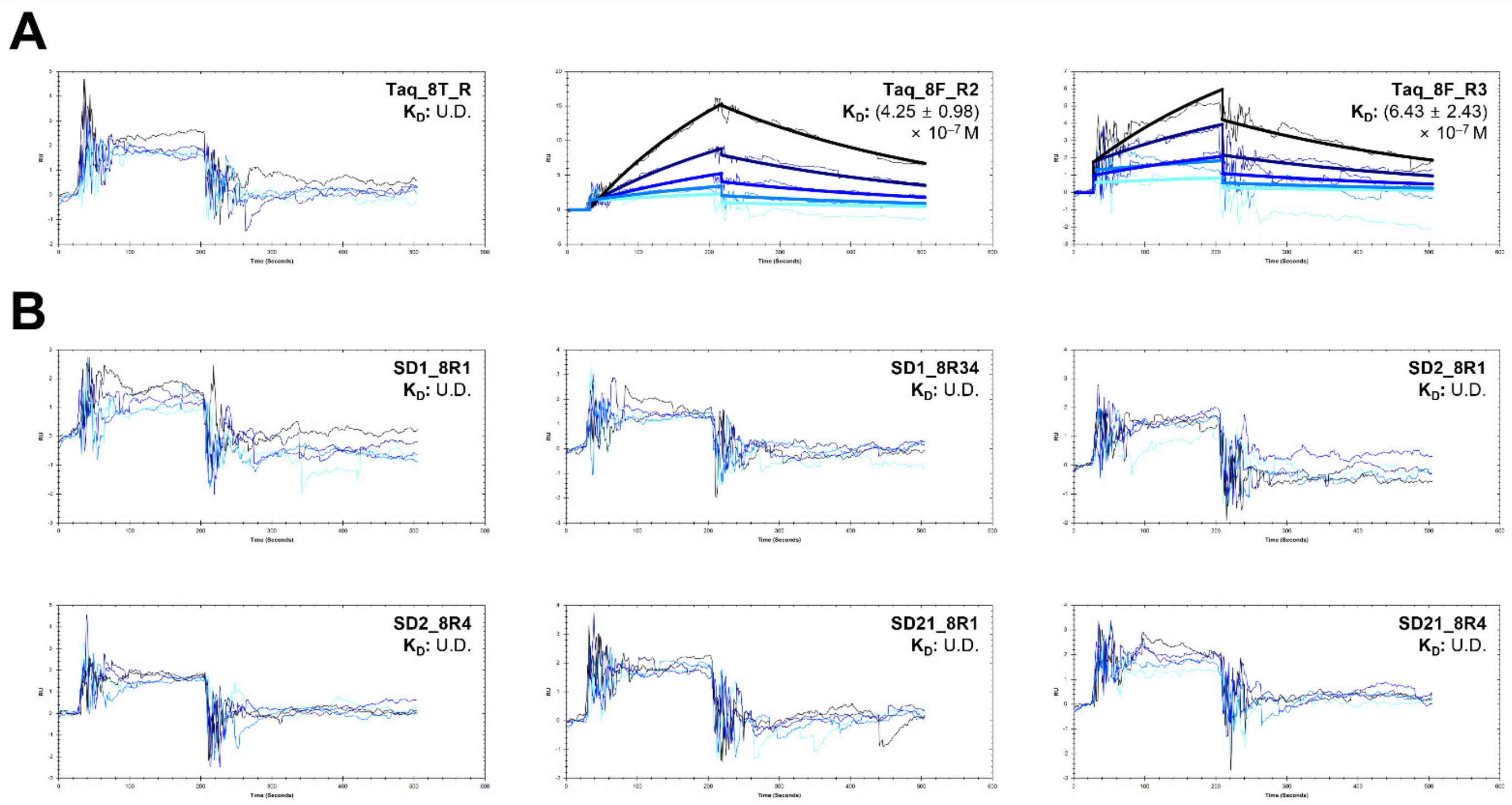

Supplementary Figure S19. Non-specific binding analysis of high-ranked aptamer libraries. Binding analysis of (**A**) the aptamers candidates from Taq groups to thrombin and (**B**) the non-G-quadruplex structured sequences from SD groups by a SPR system at 25°C. Each aptamer's dissociation constant (K_D) to targeted thrombin were determined. Data are presented as the mean ± SD of three independent measurements (n = 3). K_D values were calculated individually from k_{off}/k_{on} for each measurement.

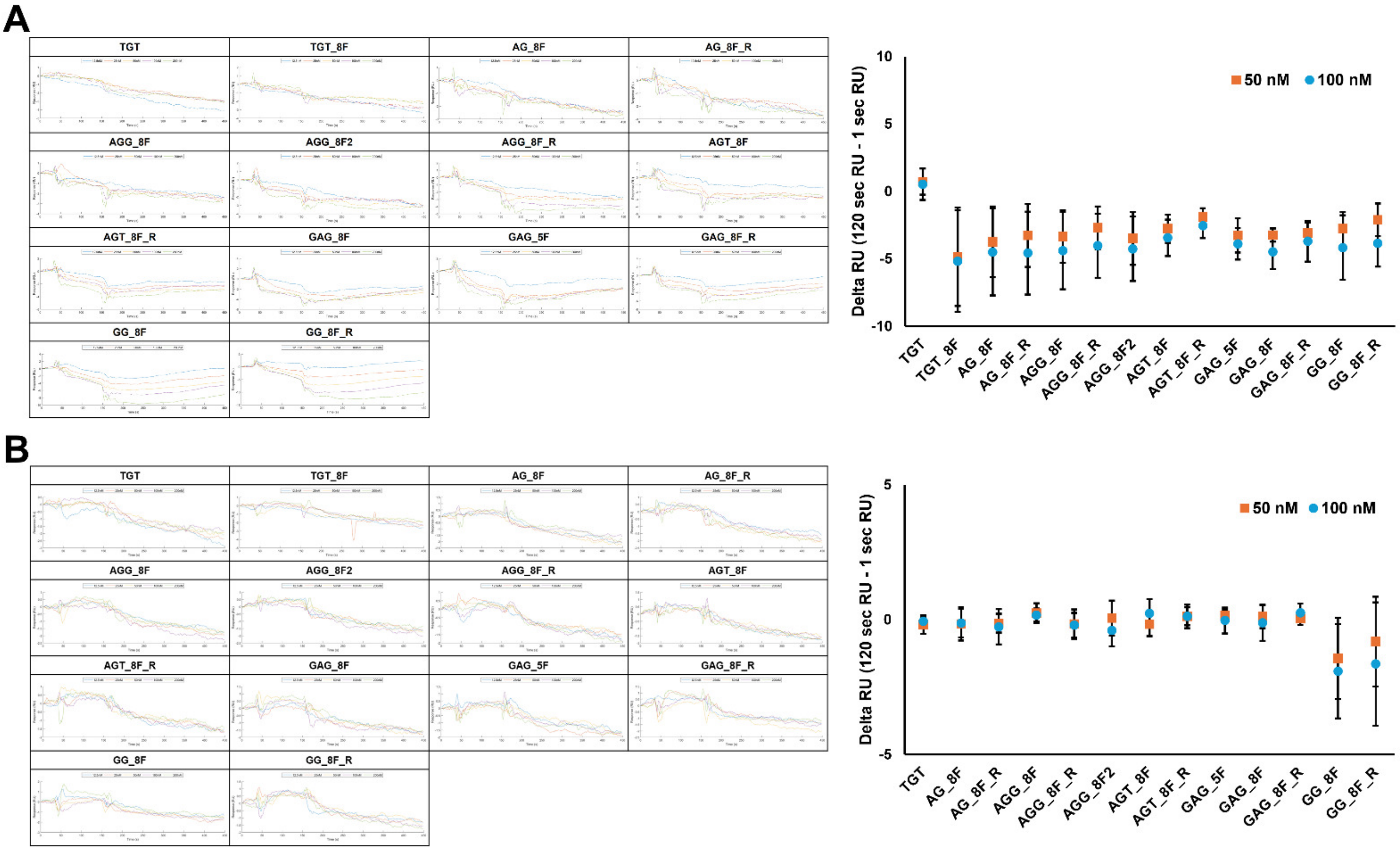

Supplementary Figure S20. Specificity analysis of thrombin aptamer candidates selected through SD-SELEX against non-target proteins. The specificity of the thrombin aptamer candidates was evaluated against the non-target proteins (**A**) VEGF165 and (**B**) MUC1. Concentration-dependent binding responses and the maximum change in response units (RU), calculated as the maximum association RU minus the baseline RU, were analyzed for each aptamer candidate. Data are presented as the mean ± SD of three independent experiments (n = 3).

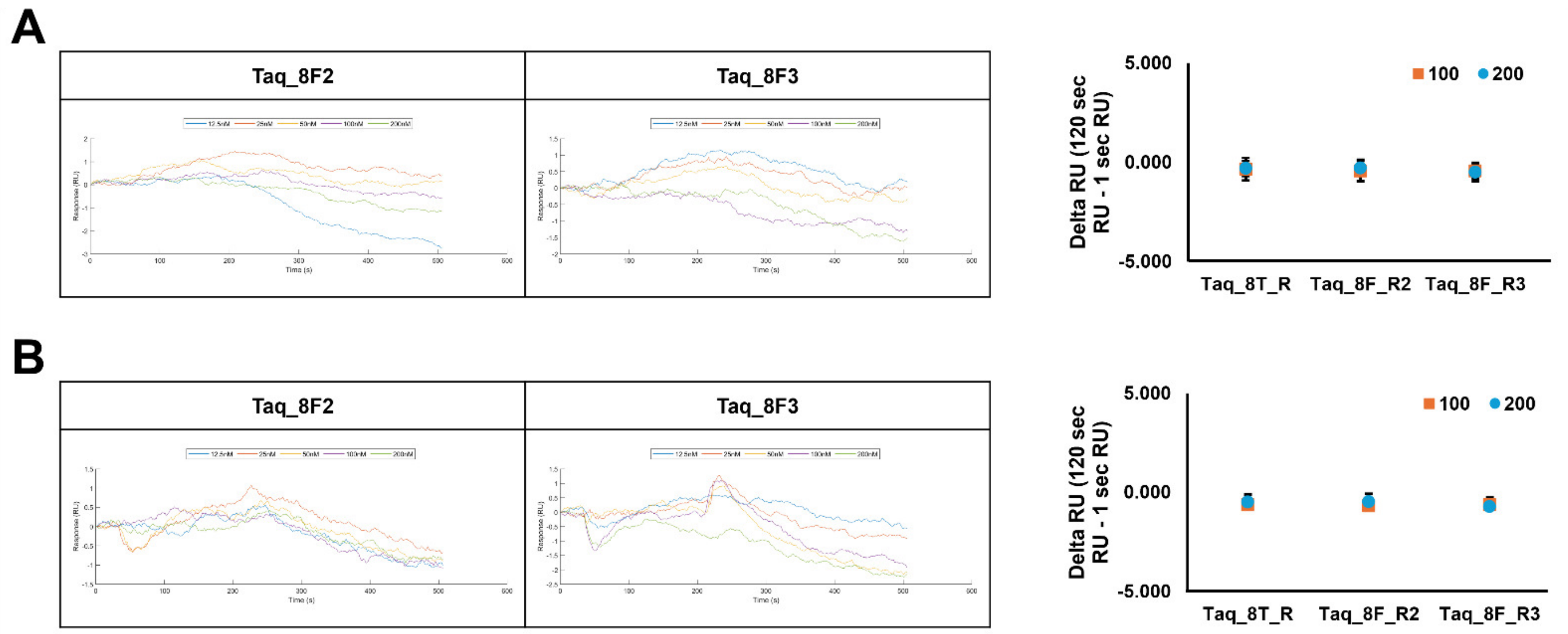

Supplementary Figure S21. Specificity analysis of thrombin aptamer candidates selected through Taq-SELEX against non-target proteins. The specificity of the thrombin aptamer candidates was evaluated against the non-target proteins (**A**) VEGF165 and (**B**) MUC1. Concentration-dependent binding responses and the maximum change in response units (RU), calculated as the maximum association RU minus the baseline RU, were analyzed for each aptamer candidate. Data are presented as the mean ± SD of three independent experiments (n = 3).

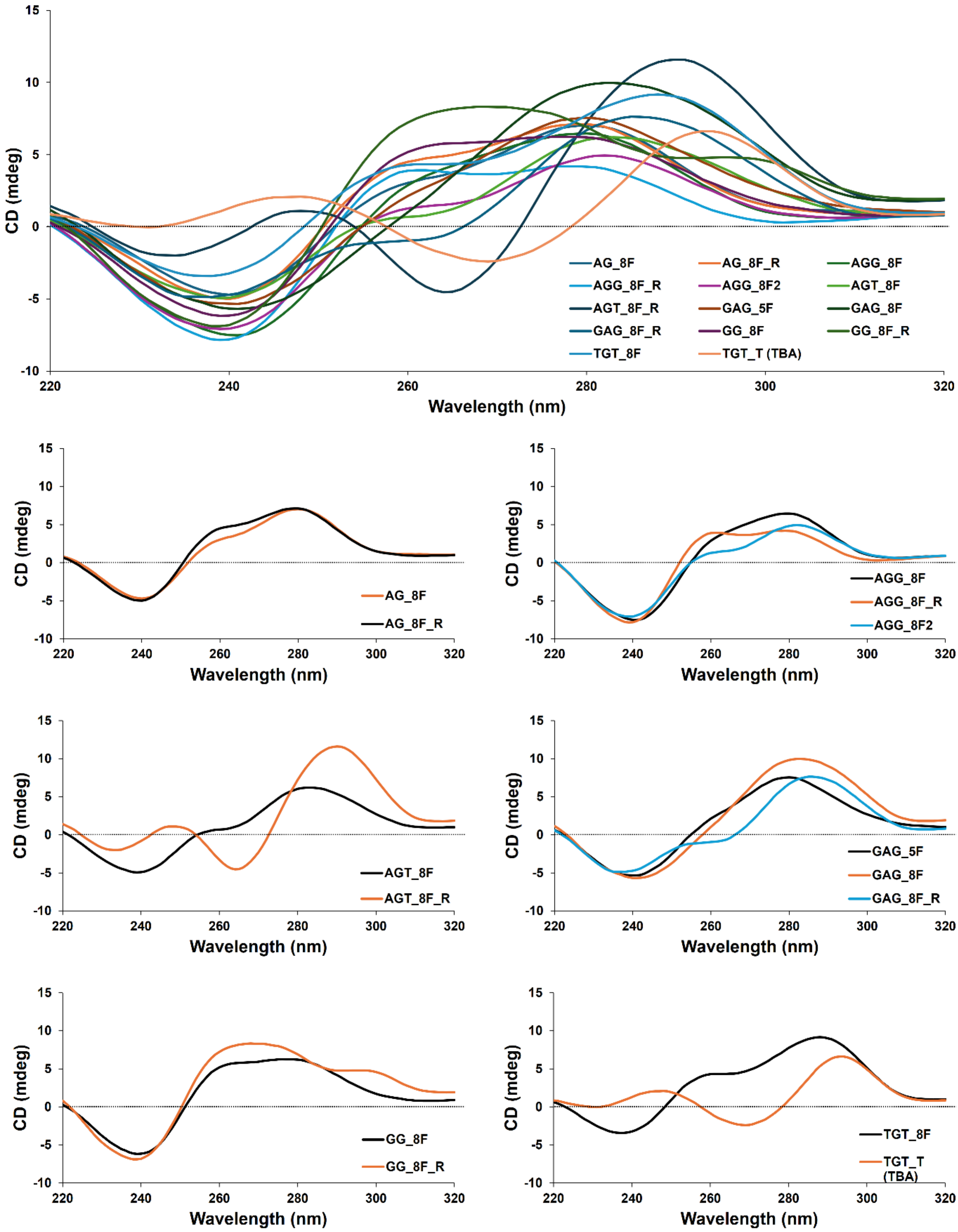

Supplementary Figure S22. CD spectra of selected thrombin aptamer from SD-SELEX.

A

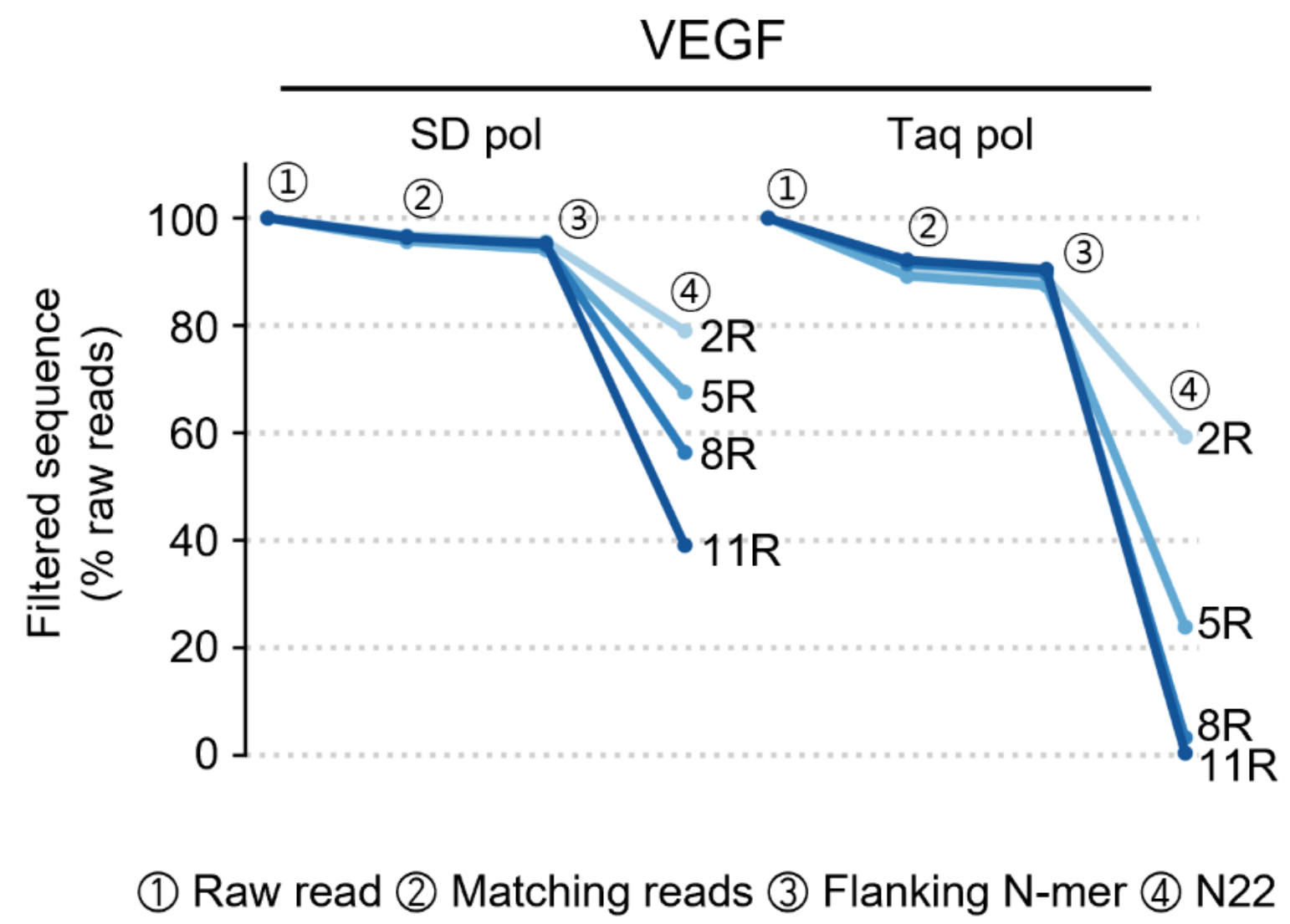

Sample	Raw reads	Matching reads	Flanking N-mer	N22
SD-2R	210,117,302	203,282,402	200,952,210	165,921,361
SD-5R	151,882,076	145,256,813	142,944,780	102,622,237
SD-8R	163,964,722	158,309,025	155,629,871	92,394,902
SD-11R	279,676,730	269,575,294	266,506,573	109,241,703
Taq-2R	178,393,836	161,855,714	158,557,140	105,723,854
Taq-5R	173,332,274	154,562,400	151,561,464	41,364,764
Taq-8R	167,312,482	153,106,127	150,082,988	5,474,273
Taq-11R	215,768,860	198,899,382	195,143,856	834,147

B

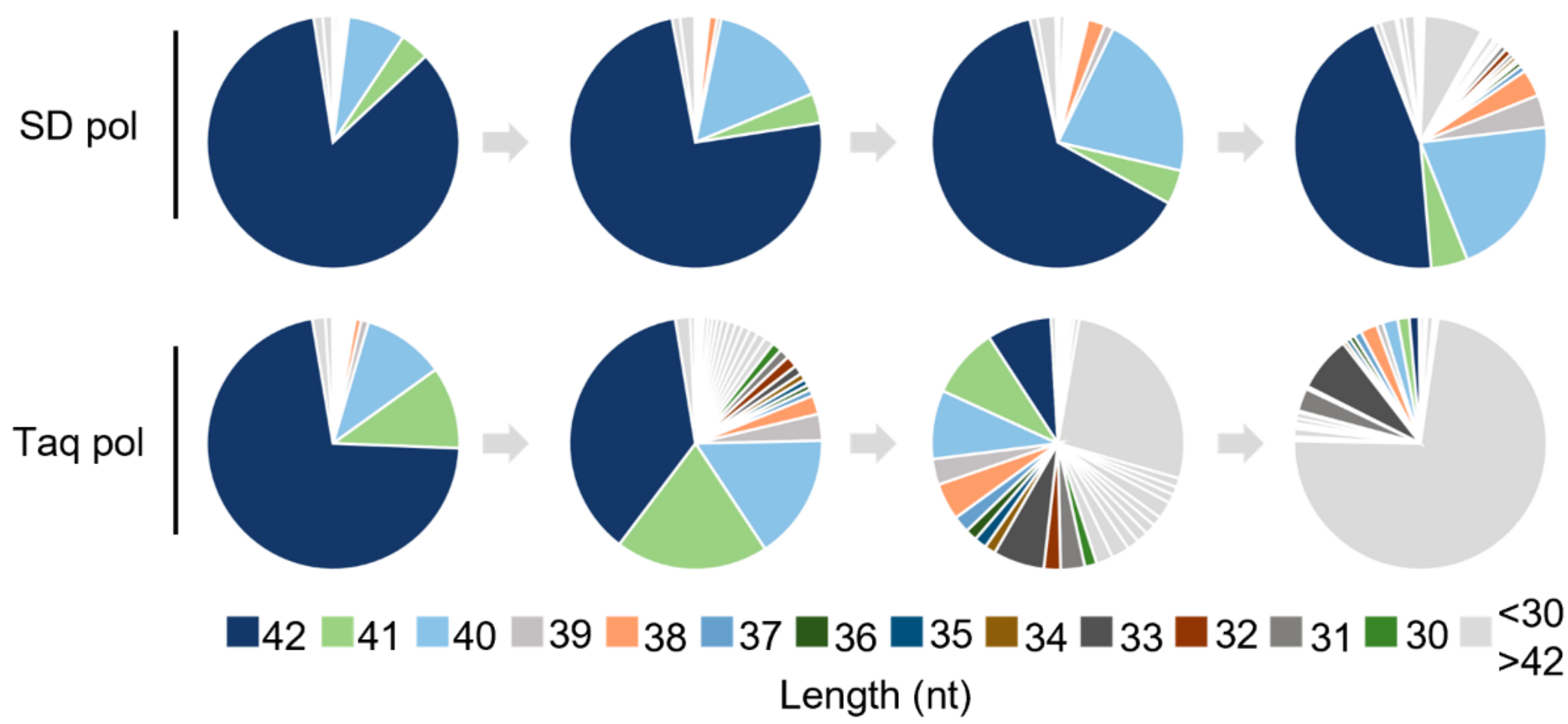

Supplementary Figure S23. Sequence recovery and flanking-region length distribution across VEGF-165 SELEX rounds using SD or Taq polymerase. (**A**) Recovery of filtered sequences, expressed as a percentage of raw reads, at successive sequence-processing steps across SELEX rounds. (**B**) Length distribution of the flanking regions across SELEX rounds. The designed 42-nt species was predominantly retained in SD-SELEX, whereas aberrant-length species increased in Taq-SELEX from round 2 onward.

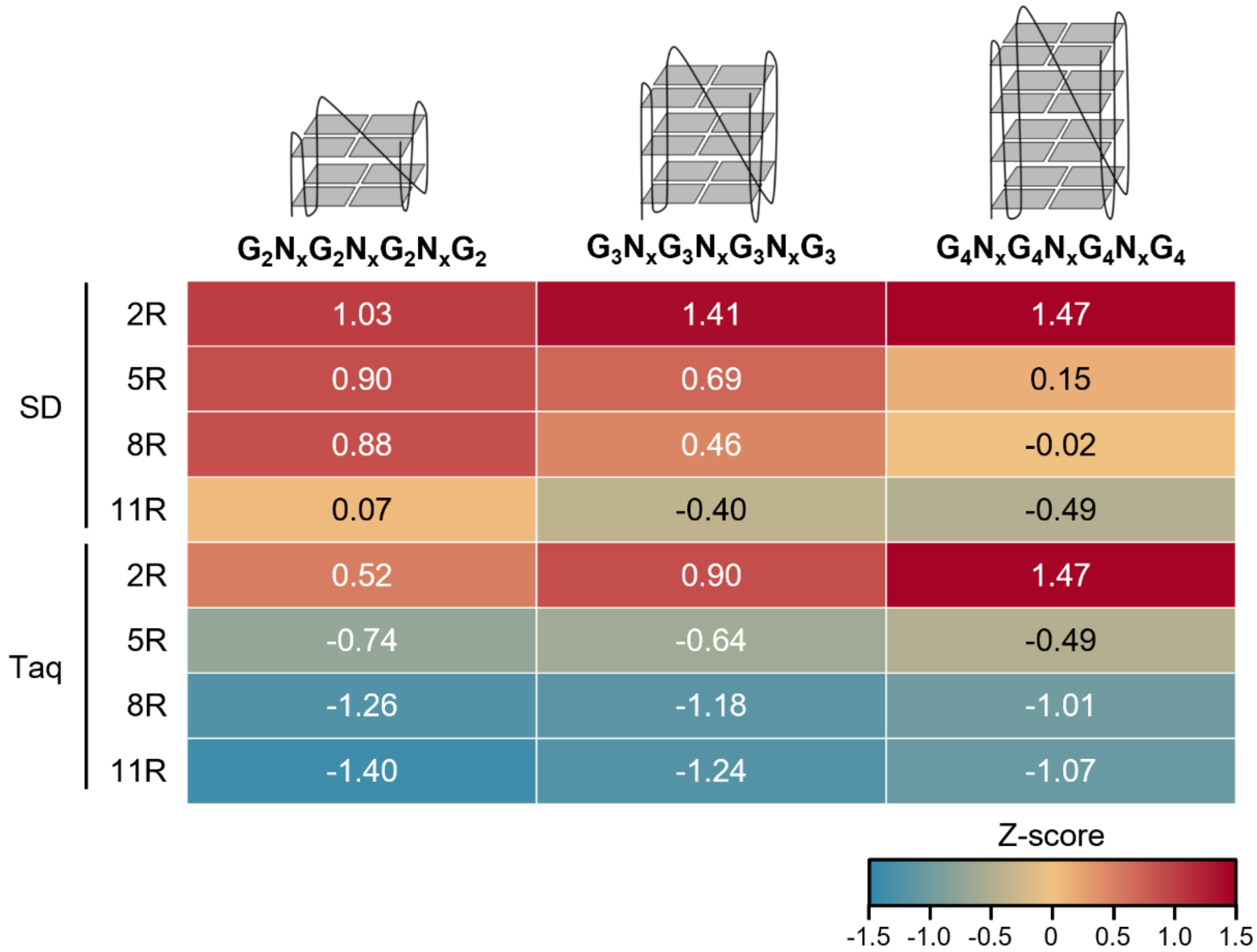

Supplementary Figure S24. G-quadruplex enrichment across rounds for VEGF-165. Proportion of reads containing 2-, 3- and 4-tetrad G-quadruplex motifs, divided by total raw reads and normalized as Z-scores within each motif type. Retained under SD, depleted under Taq after round 2.

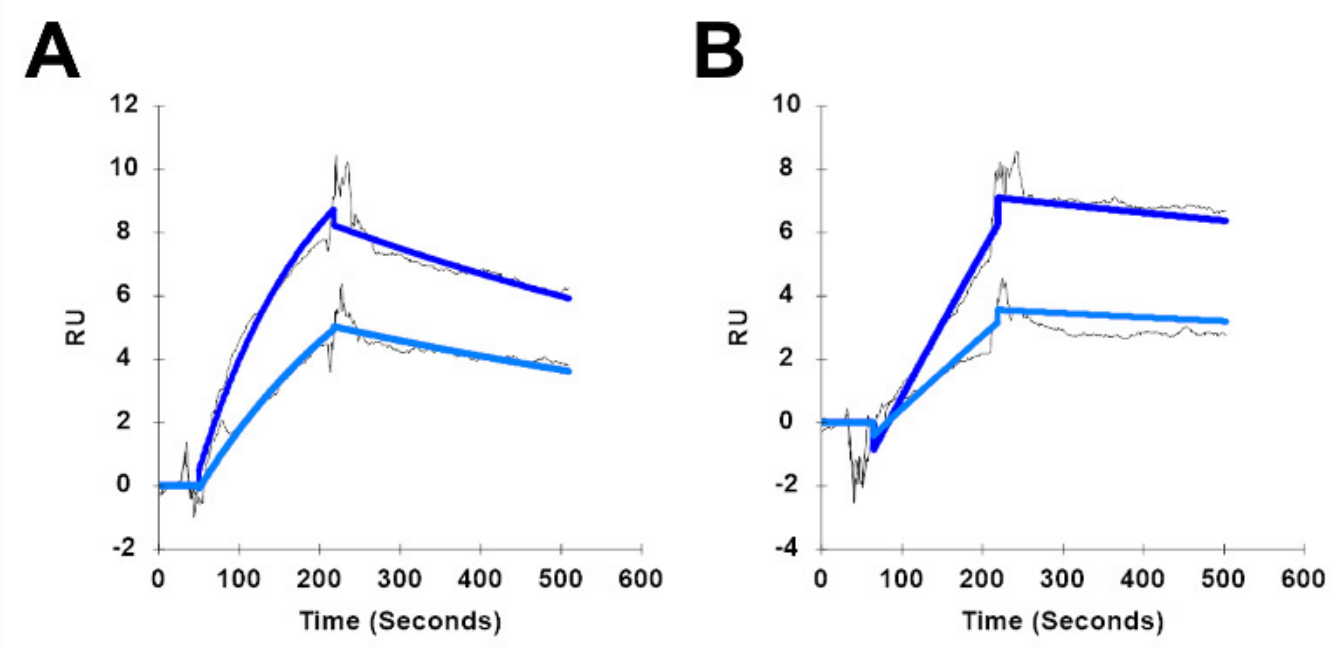

Aptamer	Replicate	K_D	K_{on} ($M^{-1} \cdot s^{-1}$)	K_{off} (s^{-1})
Vap7_1	1	5.45E-09	7.34E+04	4.00E-04
	2	4.02E-08	2.81E+04	1.13E-03
	3	8.30E-08	1.32E+05	1.09E-02
Vap7_2	1	2.86E-06	9.83E+02	2.81E-03
	2	5.00E-06	4.32E+02	2.16E-03
	3	1.61E-06	2.37E+02	3.82E-04

Supplementary Figure S25. SPR sensorgrams for two motif-containing sequences (A, Vap7_1; B, Vap7_2) recovered from the SD-SELEX VEGF-165 pool. Both bind VEGF-165 in a concentration-dependent manner. The table lists the constants obtained from each of three independent measurements (n = 3); K_D values were calculated individually from k_{off}/k_{on} for each measurement. Responses were small, and the values varied by up to 15-fold between replicates; they are therefore given per replicate, without a mean.

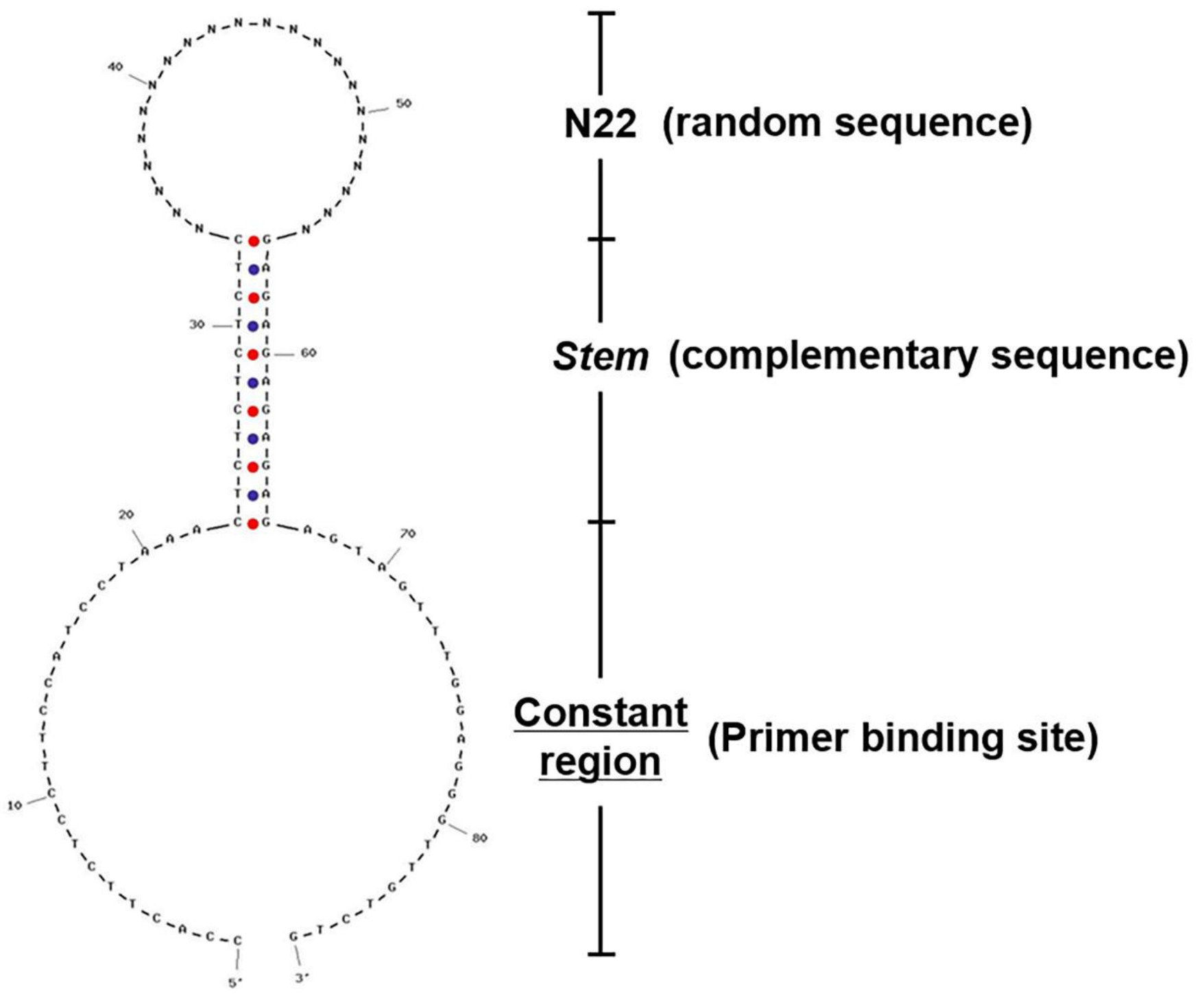

5′- CCA CTT CTC CTT CCA TCC TAA AC*T CTC TCT CTC*
NNN NNN NNN NNN NNN NNN NNN N*GA GAG AGA GA*G AGT
AGT TTG GAG GGT TGT CTG -3′

Supplementary Figure S26. Structure of the aptamer library used in this study. The aptamer library used in this study is divided into three sections. First, there is a constant region for priming described as underline. A 10-base pair of stem portions (described as italic) can increase the stability of the N22 portion and form various structures for the targets of the aptamer library. Lastly, N22 portion contains 22-mer random sequences directly involved in binding to the target and has a sequence diversity of as much as 4^{22}.

Supplementary Table S1. DNA oligo sequences used in this study.

Name	5'-Sequence-3'	Note
TEST_FW	GGC TGC CCA GCA AGT ATG ATT TGT CCT T	5' phosphorylation
TEST_RE	CAG ACT CTT CCA GCT GTT GCT CCT CCA C	5' phosphorylation
#1	GGC TGC CCA GCA AGT ATG ATT TGT CCT TGC ACC GAA GAG CGC GCA CCG ACG AGA ACA GGT GGA GGA GCA ACA GCT GGA AGA GTC TG	-
#2	GGC TGC CCA GCA AGT ATG ATT TGT CCT TGG CGG CGG CCA AAA AAA AAA GGC CGC CGC CGT GGA GGA GCA ACA GCT GGA AGA GTC TG	Stem structure
#3	GGC TGC CCA GCA AGT ATG ATT TGT CCT TGG CGG CGG CCC CGT **GGT TGG TGT GGT TGG** GGT GGG CCG CCG CCG TGG AGG AGC AAC AGC TGG AAG AGT CTG	Stem and G-quadruplex structure
#4	GGC TGC CCA GCA AGT ATG ATT TGT CCT TGC GTG GTC GCG GCC GAG GTA CAT CAC CTC GGC CGC GAC CAC GCG TGG AGG AGC AAC AGC TGG AAG AGT CTG	Stem structure
#5	GGC TGC CCA GCA AGT ATG ATT TGT CCT T**GG TCA CAC GCA GGC GGC GG**C CAA AAA AAA AAG GCC GCC GCC TTA ATC AAC TAG TGG AGG AGC AAC AGC TGG AAG AGT CTG	Stem and G-quadruplex structure
#6	GGC TGC CCA GCA AGT ATG ATT TGT CCT TGG TCA CAC GCA GCG TGG TCG CGG CCG AGG TAC ATC ACC TCG GCC GCG ACC ACG CTT AAT CAA CTA GTG GAG GAG CAA CAG CTG GAA GAG TCT G	Stem structure
#7	GGC TGC CCA GCA AGT ATG ATT TGT CCT TAG CAA GCG AGC CGC G**GG TTG GTG TGG TTG G**GT GGA GGA GCA ACA GCT GGA AGA GTC TG	G-quadruplex structure
#8	GGC TGC CCA GCA AGT ATG ATT TGT CCT TCT CTC TCT CTC CCC CCC CCC AGA GAG AGA GGT GGA GGA GCA ACA GCT GGA AGA GTC TG	Stem structure
#9	GGC TGC CCA GCA AGT ATG ATT TGT CCT TCT CTC TCT CTC CGT **GGT TGG TGT GGT TGG** GGT GAG AGA GAG AGG TGG AGG AGC AAC AGC TGG AAG AGT CTG	Stem and G-quadruplex structure
Stem_Lib_N22	CCA CTT CTC CTT CCA TCC TAA ACT CTC TCT CTC NNN NNN NNN NNN NNN NNN NNN NGA GAG AGA GAG AGT AGT TTG GAG GGT TGT CTG	3' phosphorylation and stem structure
SEL_FP	CCA CTT CTC CTT CCA TCC TAA AC	5' phosphorylation
SEL_RP	CAG ACA ACC CTC CAA ACT ACT C	5' phosphorylation
B_SEL_RP	CAG ACA ACC CTC CAA ACT ACT C	5' Biotinylation
TGT_T	**GGT TGG TGT GGT TGG**	15-mer TBA control
TGT_8F	TCT CTC TCT CCG T**GG TTG GTG TGG TTG G**GG TGG AGA GAG AGA	8 Round top tiers

AG_8F	TCT CTC TCT CTG TT**G GTT GGA GGG TTG G**TT TTG AGA GAG AGA	8 Round top tiers
AG_8F_R	TCT CTC TCT CTG AGT **GGT TGG AGG GTT GG**G TTG AGA GAG AGA	Reproducible 8 Round top tiers
AGG_8F	TCT CTC TCT CAG TA**G GTT GGA GGG GTT GG**A GCG AGA GAG AGA	8 Round top tiers
AGG_8F2	TCT CTC TCT CAT GTA **GGT TGG AGG GGT TGG** TCG AGA GAG AGA	8 Round top tiers with analytic position
AGG_8F_R	TCT CTC TCT CTT AGT A**GG TTG GAG GGG TTG G**GG AGA GAG AGA	Reproducible 8 Round top tiers
AGT_8F	TCT CTC TCT CAG TA**G GTT GGA GTG GTT GG**T TAG AGA GAG AGA	8 Round top tiers
AGT_8F_R	TCT CTC TCT CCT G**GG TTG GAG TGG TTG G**CG TAG AGA GAG AGA	Reproducible 8 Round top tiers
GAG_8F	TCT CTC TCT CAA AGT **GGT TGG GAG GGT TGG** CGG AGA GAG AGA	8 Round top tiers
GAG_5F	TCT CTC TCT CGT T**GG TTG GGA GGG TTG G**AT TTG AGA GAG AGA	5 Round top tiers with analytic position
GAG_8F_R	TCT CTC TCT CTA AG**G GTT GGG AGG GTT GG**T AAG AGA GAG AGA	Reproducible 8 Round top tiers
GG_8F	TCT CTC TCT CAT GTA **GGT TGG GGG GTT GG**G TCG AGA GAG AGA	8 Round top tiers
GG_8F_R	TCT CTC TCT CGG **GGT TGG GGG GTT GG**A GTA GAG AGA GAG AGA	Reproducible 8 Round top tiers
SD1-8R1(dG)	TCT CTC TCT CCA CTC GCG ATA CGA GAG TGA ACG AGA GAG AGA	Non-G-quadruplex structure
SD1-8R34(dG)	TCT CTC TCT CAT TCG TTG AAA TGA ACG AAG AAG AGA GAG AGA	
SD2-8R1(dG)	TCT CTC TCT CTT GCG AGC GCG CTG AAC GCG CAG AGA GAG AGA	
SD2-8R4(dG)	TCT CTC TCT CGC TGA CTG CAT AAC CGA TGC AGG AGA GAG AGA	
SD21-8R1(dG)	TCT CTC TCT CAC ATA AGT GTC TTT TAG TTA CCG AGA GAG AGA	
SD21-8R4(dG)	TCT CTC TCT CAC AGA CCG TCG ACA TTG CGA AGG AGA GAG AGA	
HY_Taq_8T_R	**GTT TGG GGG GTT GG**	Selected candidate in Taq groups
HY_Taq_8F_R2	TCT CTC TCT CGT T**GT TTG GGG GGT TGG** GTT TGG AGA GAG AGA	
HY_Taq_8F_R3	TCT CTC TCT CAA TA**G TTT GGG GGG TTG G**TC GGG AGA GAG AGA	

Note: Bold and underlined nucleotides indicate regions involved in G-quadruplex and stem formation, respectively.

Supplementary Table S2. SELEX conditions employed in various papers using target-immobilized SELEX.

Paper	Target	NO. of Random sequences	NO. of selection Round	Polymerase	NO. of PCR cycles	Aptamer K_D
Nature 355.6360 (1992): 564-566.	Thrombin	60	5	-	-	
Bioorganic & medicinal chemistry 9.10 (2001): 2543-2548.	L-Tyrosinamide	60	15	Taq DNA polymerase	Under 12	pE35: 45 (μM)
Biotechniques 32.1 (2002): 178-183.	Cholera whole toxin staphylococcal enterotoxin B	20	5	Taq DNA polymerase	40	-
Analytical and bioanalytical chemistry 383 (2005): 83-91.	Streptavidin	60	13	Taq DNA polymerase	30	26: 85.2<br>30: 65.5<br>31: 56.7<br>33: 59.0<br>(nM)
Biochemical and biophysical research communications 338. 4 (2005): 1928-1934.	Ethanolamine	60	9	Taq DNA polymerase	30	#14.3: 6<br>#11.4: 9<br>#2.3: 17<br>#9.6: 15<br>#9.4: 10<br>#9.10<br>(nM)
Bioorganic & medicinal chemistry 16.15 (2008): 7245-7253.	Tetracyclin	40	4	Taq DNA polymerase	30	No.4: 9.6<br>No.5: 12.0<br>No.20: 56.8<br>(nM)
Acta Biochim Biophys Sin 41.4 (2009): 335-340.	Streptavidin	25	12	Taq DNA polymerase	5	
Analyst 136.3 (2011): 577-585.	L-tryptophan	45	12	Taq DNA polymerase	10 ~ 20	Trp3a-1: 1.757
Analytical Chemistry 84.3 (2012): 1669-1676.	Polychlorinated biphenyls	40	9	Taq DNA polymerase	-	9.1: 128<br>9.2: 119<br>9.3: 99<br>(nM)
Analytica chimica acta 748 (2012): 67-72.	digoxin	50	10	Taq DNA polymerase	30	D1: 0.05 (nM)
PloS one 7.4 (2012): e35253.	ß-conglutin	49	14	Tfi DNA polymerase	18	2: 515 (nM)<br>40: 360 (nM)
PLoS One 7.2 (2012): e31970.	MUC1	40	-	Taq DNA polymerase	25	MA3: 38.3 (nM)

Nucleic acid therapeutics 23.5 (2013): 322-331.	Abscisic acid	40	7	-	25	Clone 2: 0.98<br>Clone 9: 0.80<br>(μM)
Analytical Methods 6.3 (2014): 690-697.	Staphylococcus aureus enterotoxin A	40	12	Taq DNA polymerase	22	A1: 92.17<br>A15: 48.57<br>A23.2: 235.00<br>(nM)
Microchimica Acta 181.11 (2014): 1317-1324.	Fumonisin B1	40	13	-	16-25	F1: 132<br>F8: 89<br>F10: 62<br>(nM)
Applied biochemistry and biotechnology 180.8 (2016): 1644-1656.	Chloramphenicol	40	10	Taq DNA polymerase	30	NO.4: 0.102<br>NO.5: 0.032<br>(μM)
PLoS One 12.12 (2017): e0189558.	EpCAM	45	11	Taq DNA polymerase	-	Ep1: 118<br>(nM)
ACS omega 3.10 (2018): 13576-13583.	Atrazine	34	12	GoTaq DNA polymerase	-	R12.45 Truncated: 3.7<br>(nM)
Journal of agricultural and food chemistry 66.37 (2018): 9801-9809.	Domoic acid (DA)<br>Saxitoxin (STX)<br>Tetrodotoxin (TTX)	40	16	Taq Plus DNA polymerase	20	DA-01: 134.70<br>DA-06: 62.07<br>TTX-07: 44.12<br>TTX-27: 90.73<br>STX-41: 61.44<br>(nM)
Photochemical & Photobiological Sciences 17.6 (2018): 800-806.	Crystal violet	30	11	rTaq DNA polymerase	14	CV30S: 0.49<br>(μM)
Microchemical Journal 159 (2020): 105354.	Diethylstilbestrol	40	11	Taq DNA polymerase	19	DES-03: 126.3<br>(nM)
Nature Biotechnology (2022): 1-9.	thrombin	30	9	FastPfu Fly DNA polymerase	D-DNA: 15~30<br>L-DNA: 10~30	L-13: 22<br>L-13t: 34<br>(nM)
Analytical Chemistry 95.44 (2023): 16366-16373.	SDM-3, MIX-1, SMZ	30	12 or 15	Taq DNA polymerase		SDM-3: 219-630<br>MIX-1: 89-740

						SQX-3: 320 (nM)
Scientific Reports 13.1 (2023): 14560.	SARS-CoV-2 spike receptor binding domain (RBD)	40	10	Taq DNA polymerase	30	125.26 (nM)
ACS Applied Materials & Interfaces 15.9 (2023): 11599-11608.	TNFα-TNF receptor 1	40	12	Taq DNA polymerase	30	Apt1: 244.2<br>Apt1-67nt: 276.4<br>Apt1-49nt: 173.2<br>Apt2: 169.4<br>Apt2-55nt: 133.5<br>(nM)
Nature Chemistry 15.12 (2023): 1705-1714.	Sulfur (VI) fluoride exchange (SuFEx)	28 random B bases	13	Hot start Taq DNA polymerase	20	Seq1: 2.05<br>Seq2: 27.8<br>(nM)
Journal of the American Chemical Society 145.13 (2023): 7540-7547.	ATP adenosine	36	ATP: 10<br>Adenosine: 13	Taq DNA polymerase	-	Adenosine-<br>Ade1301b: 0.2<br>Ade1304b: 0.4<br>ATP-<br>Ade1301b: 2.5<br>Ade1304b: 2.3<br>(μM)
Nucleic Acids Research 51.20 (2023): 11375-11385.	Kanamycin A<br>Kanamycin B	40 with 10	13	Taq DNA polymerase	5	T1-U42C: 1.1<br>T1_BS1: 2.1<br>T1_BS2: 123<br>(nM)

Supplementary Table S3. SELEX conditions per each round.

Round	Pool size (fmole / N22 library number)	Target size (fmole / thrombin number)
1	1.46×10^{6} (8.8×10^{14})	1.46×10^{4} (8.8×10^{12})
2	90 (5.4×10^{10})	9 (6.0×10^{9})
3	100 (6.0×10^{10})	10 (6.0×10^{9})
4	80 (4.8×10^{10})	8 (4.8×10^{9})
5	100 (6.0×10^{10})	10 (6.0×10^{9})
6	100 (6.0×10^{10})	10 (6.0×10^{9})
7	200 (1.2×10^{11})	10 (6.0×10^{9})
8	200 (1.2×10^{11})	10 (6.0×10^{9})
9	160 (9.6×10^{10})	8 (4.8×10^{9})
10	200 (1.2×10^{11})	10 (6.0×10^{9})
11	100 (6.0×10^{10})	5 (3.0×10^{9})

Supplementary Table S4. Summary of qPCR Ct values and PCR cycles (1st to 11th rounds).

Experimental group		SELEX round										
		**1**	**2**	**3**	**4**	**5**	**6**	**7**	**8**	**9**	**10**	**11**
**SD 1st batch**	Ct value	12.2	15.9	16.9	16.1	15.9	17.9	16.6	15.9	15.9	12.8	15.1
	*PCR cycle	6	10	11	10	10	12	11	10	10	7	-
**SD 2nd batch**	Ct value	11.6	15.9	17.9	13.8	15.9	16.8	15.9	16.9	13.9	15.0	15.6
	*PCR cycle	6	10	12	8	10	11	10	11	8	9	-
**Taq 1st batch**	Ct value	12.8	15.2	18.9	14.0	15.9	15.8	17.9	14.1	13.9	13.7	13.8
	*PCR cycle	7	9	13	8	10	10	12	8	8	8	-
**Taq 2nd batch**	Ct value	12.8	13.9	16.8	12.0	15.0	16.8	16.9	14.8	12.9	13.8	13.9
	*PCR cycle	7	8	11	6	9	11	11	9	7	8	-
**Control 1st batch**	Ct value	12.8	11.2	10.9	10.8	10.1	9.8	10.9	9.8	10.0	10.8	11.8
	**PCR cycle	13	11	11	11	11	11	12	11	11	12	-
**Control 2nd batch**	Ct value	13.4	12.8	10.8	10.8	10.8	10.3	10.8	10.2	9.8	9.9	11.9
	**PCR cycle	13	13	11	11	12	11	12	11	11	11	-

Supplementary Table S5. Source and biochemical properties of the two DNA polymerases used in this study - the strand-displacement (SD) polymerase used for SD-SELEX and the Taq polymerase used for conventional SELEX.

Property	SD polymerase (SD-SELEX)	Taq polymerase (conventional SELEX)
**Commercial source**	SD HotStart DNA polymerase, Bioron GmbH (Römerberg, Germany)	i-StarTaq DNA polymerase, Intron Biotechnology (Seongnam, Republic of Korea)
**Enzyme class**	Strand-displacing thermostable DNA polymerase	Conventional thermostable DNA polymerase
**Strand-displacement activity**	Strong — synthesizes continuously while displacing downstream duplex regions	Limited / essentially absent — stalls at stable secondary structures
**Amplification of structured templates**	Traverses structured (e.g. G4-forming) templates without accessory helicase	Biased against highly structured templates
**5′→3′ exonuclease activity**	Absent	Present
**3′→5′ proofreading**	Absent	Absent
**Thermostability**	Thermostable; hot-start format (up to ~94–95 °C)	Thermostable (up to ~95 °C)
**Components**	400 mM Tricine KOH (pH 8.8), 150 mM KOAc, 35 mM $Mg(OAc)_2$, 37.5 mg/ml BSA, 0.05% Tween 20, 0.05% IGEPALCA 630 (10X)	300 mM Tris-HCl (pH9.0), 300 mM Salts containing of K^+ & NH_4^+, 20 mM Mg^{2+}, Stabilizer and enhancer (10X)

Supplementary Table S6. Information on the number of N22 sequences.

			Total N22		Top10% N22			Top10% reproducible N22			
			Unique	Weighted	Unique	Weighted	%total N22	Unique	Weighted	%total N22	Mean GC%
SD	1st batch	2R	23,307,527	73,690,673	2,330,753	13,475,668	18.3	2,262	18,268	0.0	58.0
		5R	16,812,998	65,744,301	1,681,300	16,115,826	24.5	16,971	339,104	0.5	57.4
		8R	5,519,516	49,240,902	551,952	33,423,535	67.9	47,730	6,390,129	13.0	55.5
		11R	1,066,328	29,070,730	106,633	25,167,473	86.6	21,853	8,102,195	27.9	53.4
	2nd batch	2R	18,964,611	57,955,671	18,964,461	10,072,999	17.4	2,262	15,000	0.0	57.5
		5R	17,572,213	57,965,862	1,757,221	11,704,616	20.2	16,971	166,950	0.3	57.2
		8R	9,341,166	64,262,182	934,117	33,376,885	51.9	47,730	6,867,567	10.7	55.3
		11R	3,427,351	55,853,035	342,735	45,343,017	81.2	21,853	10,016,541	17.9	53.8
Taq	1st batch	2R	12,760,105	43,502,902	1,276,011	8,896,466	20.5	6,029	47,924	0.1	55.8
		5R	2,071,302	9,245,170	207,130	2,648,434	28.6	3,094	69,302	0.8	53.9
		8R	134,159	1,814,285	13,416	1,319,943	72.8	2,090	346,293	19.1	52.6
		11R	29,035	644,340	2,904	497,663	77.2	563	168,053	26.1	51.2
	2nd batch	2R	14,467,340	40,140,787	1,446,734	6,503,444	16.2	6,029	30,853	0.1	55.7
		5R	2,100,929	7,709,821	210,093	1,605,908	20.8	3,094	30,189	0.4	54.2
		8R	244,662	2,677,677	24,466	1,778,794	66.4	2,090	269,493	10.1	53.0
		11R	72,580	1,106,700	7,258	796,487	72.0	563	112,255	10.1	52.1
